# Genetically proxied low-density lipoprotein cholesterol lowering via PCSK9-inhibitor drug targets and risk of congenital malformations

**DOI:** 10.1093/eurjpc/zwad402

**Published:** 2024-06-03

**Authors:** Maddalena Ardissino, Eric A.W. Slob, Rohin K. Reddy, Alec P. Morley, Art Schuermans, Phoebe Hill, Catherine Williamson, Michael C. Honigberg, Antonio de Marvao, Fu Siong Ng

**Affiliations:** 1 National Heart and Lung Institute, Imperial College London, Hammersmith Campus, London, UK; 2 Department of Medicine, School of Clinical Medicine, University of Cambridge, London, UK; 3 MRC Biostatistics Unit, School of Clinical Medicine, University of Cambridge, Cambridge, UK; 4 Department of Applied Economics, Erasmus School of Economics, Erasmus University Rotterdam, Rotterdam, The Netherlands; 5 Erasmus University Rotterdam Institute for Behavior and Biology, Erasmus University Rotterdam, Rotterdam, The Netherlands; 6 Program in Medical and Population Genetics and Cardiovascular Disease Initiative, Broad Institute of Harvard and MIT, Cambridge, MA, USA; 7 Cardiovascular Research Center and Center for Genomic Medicine, Massachusetts General Hospital, Harvard Medical School, Boston, MA, USA; 8 Department of Cardiovascular Sciences, KU Leuven, Leuven, Belgium; 9 Royal Oldham Hospital, Northern Care Alliance NHS Foundation Trust, Manchester, UK; 10 Institute of Reproductive and Developmental Biology, Imperial college London, London, UK; 11 Cardiology Division, Department of Medicine, Massachusetts General Hospital, Boston, MA, USA; 12 British Heart Foundation Centre of Research Excellence, School of Cardiovascular Medicine and Sciences, King’s College London, London, UK; 13 Medical Research Council, London Institute of Medical Sciences, Imperial College London, London, UK

**Keywords:** Low-density lipoprotein, *PCSK9*-inhibitors, congenital malformations, pregnancy, Mendelian randomization

## Abstract

**Aims:**

Current guidelines advise against the use of lipid-lowering drugs during pregnancy. This is based only on previous observational evidence demonstrating an association between statin use and congenital malformations, which is increasingly controversial. In the absence of clinical trial data, we aimed to use drug-target Mendelian randomization to model the potential impact of fetal LDL-lowering, overall and through *PCSK9* drug targets, on congenital malformations.

**Methods and results:**

Instrumental variants influencing LDL levels overall and through *PCSK9*-inhibitor drug targets were extracted from genome-wide association study (GWAS) summary data for LDL on 1 320 016 individuals. Instrumental variants influencing circulating PCSK9 levels (pQTLs) and liver *PCSK9* gene expression levels (eQTLs) were extracted, respectively, from a GWAS on 10 186 individuals and from the genotype-tissue expression project. Gene-outcome association data was extracted from the 7th release of GWAS summary data on the FinnGen cohort (*n* = 342 499) for eight categories of congenital malformations affecting multiple systems. Genetically proxied LDL-lowering through *PCSK9* was associated with higher odds of malformations affecting multiple systems [OR 2.70, 95% confidence interval (CI) 1.30–5.63, *P* = 0.018], the skin (OR 2.23, 95% CI 1.33–3.75, *P* = 0.007), and the vertebral, anorectal, cardiovascular, tracheo-esophageal, renal, and limb association (VACTERL) (OR 1.51, 95% CI 1.16–1.96, *P* = 0.007). An association was also found with obstructive defects of the renal pelvis and ureter, but this association was suggestive of horizontal pleiotropy. Lower *PCSK9* pQTLs were associated with the same congenital malformations.

**Conclusion:**

These data provide genetic evidence supporting current manufacturer advice to avoid the use of *PCSK9* inhibitors during pregnancy.

## Lay summary

Using genetic techniques to mimic the effects of PCSK9-inhibitors, a group of lipid-lowering medications, this study provides evidence to support recommendations to avoid the use of these medications in pregnancy due to potential risk of multiple malformations in the newborn.

• This study provides genetic evidence to support potential associations of PCSK9-inhibitor medications with newborn malformations affecting multiple organ systems, the skin, and a cluster of structural defects simultaneously affecting the spine, anus/rectum, heart, throat, kidneys, arms and legs.

• There was also weaker evidence of an association of PCSK9-inhibitor medications with newborn malformations resulting in blockages of the kidneys and urine system, though the evidence was less certain for these than for the other malformations.

## Introduction

Elevated low-density lipoprotein (LDL) is a cardinal risk factor for cardiovascular disease.^1^ Proprotein convertase subtilisin–kexin type 9 (*PCSK9*)-inhibiting therapies, including monoclonal antibodies (mAb) and silencing RNA therapies (siRNA), can achieve profound, long-lasting reductions in LDL. Based on current guidelines, as many as 4% of the adult population are eligible for PCSK9 mAb therapies,^2^ and this is set to increase with progressively lower treatment targets. Recently, the Food and Drug Administration expanded their remit by approving their use for familial hypercholesterolaemia down to age 10.^3^

The use of LDL-lowering therapies during pregnancy is currently avoided except in very severe cases. This is partly due to concerns regarding a previously reported association of statin use with congenital malformations,^4^ though opinions regarding the true causal nature of this association are conflicted, as subsequent studies have not replicated it.^5,6^ Overall, however, the concern appears biologically justified: LDL metabolism is central to the development of the fetus, playing key roles in cell proliferation as well as sonic hedgehog signalling, both of which are important during human development. Additionally, *PCSK9* is known to play an important role in regulating fetal LDL levels, both through its direct role in regulating fetal LDL metabolism, and through modulation of LDL-receptor expression on the placenta regulating maternal-to-fetal LDL transport.^7^ Indeed, it is known that oligogenic conditions perturbing LDL synthesis, such as Smith-Lemli-Opitz syndrome (SLOS), are associated with high risk of congenital abnormalities.^8^ In addition to this, previous evidence has suggested an association of the loss-of-funotion R46L mutation in the PCSK9 gene with risk of neural tube defects,^9^ though this association was only nominally statistically significant in the setting of a phenome-wide association study.

Given these biologically important safety concerns, clinical trials to test the safety of PCSK9 inhibitors are not ethically justified. This implies that there is currently no randomized evidence to either support or refute the hypothetical risks of congenital malformations associated with PCSK9 inhibitor use in pregnancy. In clinical practice, despite the lack of clinical data, the medication is advised against by manufacturers.^10,11^ The ongoing contraindication of these therapies in the setting of limited evidence-based data does disservice to women who rely on these therapies for LDL-lowering. Upon discontinuation of this drug during pregnancy, the exposure to high LDL levels lasting the pregnancy, which will be further aggravated by the physiological increase in LDL occurring secondary to the pregnancy itself, will contribute to an increase in lifetime maternal and offspring atherosclerotic disease risk. Further investigation is needed to provide additional evidence to either corroborate or question the current recommendation against use of these agents in pregnancy.

In the absence of clinical trial data, drug-target Mendelian randomization (MR) can be used to inform potential efficacy and safety^12^ of medications. Drug-target MR leverages the natural variability in genetic variants encoding drug targets to explore potential effects of their perturbation. Since allocation of genetic variants occurs randomly through the process of mating and allele assortment at conception, this is akin to randomization in a clinical trial. Importantly, when modelling the potential fetal effects of administration of a drug in pregnancy, the framework behind drug-target MR can only hold when modelling direct fetal effects of drug-target perturbation in the fetus directly. Inthiscase, this assumption can be assumed to hold, because it is established that both mAb and siRNA molecules cross the placenta.^10,11^ However, it must be highlighted that the framework is only strictly applicable to agents that cross the placenta, and that it models the potential effects of LDL metabolism perturbation in the fetus rather than in the mother. In this study, we aim to leverage drug-target MR to model the potential impact of LDL-lowering, overall and through *PCSK9*-inhibition, on risk of congenital malformations.

## Methods

### Ethical approval, data availability, and reporting

Data used in this study is publicly available and all relevant sources are cited. Ethical approval and participant consent were obtained in the original studies that generated the data. Statistical analysis was carried out using R version 4.2.2 (2022-10-31).^13^

### Instrumental variable selection

Genetic association estimates for LDL were acquired from the most recent genome-wide association study (GWAS) on 1 320 016 European ancestry individuals included in the global lipids genetic consortium (GLGC).^14^ Uncorrelated (r^2^ < 0.1) single-nucleotide polymorphisms associated with LDL (*P* < 5 × 10^−8^) overall and in the *PCSK9* gene region ±10kB (Table 1, derived from DrugBank^15^) were selected as instrumental variants. The final instrumental variants utilized in the analysis and respective association estimates with LDL are reported in Supplementary material online, Tables S1 and S2.

In addition to the drug-target MR, we tested the associations of circulating PCSK9 protein levels and *PCSK9* gene expression in the liver with congenital malformations. For these analyses, genome-wide significant (*P* < 5 × 10^−8^) uncorrelated (*r*^2^ < 0.1) instrumental variants acting in *cis* (±100kB from *PCSK9* gene region) were extracted for PCSK9 protein levels in whole blood [PCSK9 protein quantitative trait loci (pQTLs)] from a GWAS on 10 186 individuals,^16^ and for *PCSK9* gene expression levels in the liver [*PCSK9* expression quantitative trait loci (eQTLs)] from the genotype-tissue expression (GTEx) Project summary data (Version 8, *n* = 266).^17,18^ Because pQTLs and eQTLs were extracted from association studies with limited sample sizes, the *cis* region for instrument selection was expanded to ±100 kb to increase power. The final instrumental variants utilized are reported in Supplementary material online, Tables S3 and S4.

A validation analysis for the primary drug-target MR was carried out by repliating the entire workflow utilizing data from Neale Lab’s R2 data including 469 897 UK Biobank participants (http://www.nealelab.is/uk-biobank/). Similar to the main analysis, uncorrelated (*r*^2^ < 0.1) single-nucleotide polymorphisms associated with LDL (*P* < 5 × 10^−8^) overall, and in the *PCSK9* gene region ±10 kB were extracted as instrumental variants for this analysis.

### Study outcomes

**Figure 1:**
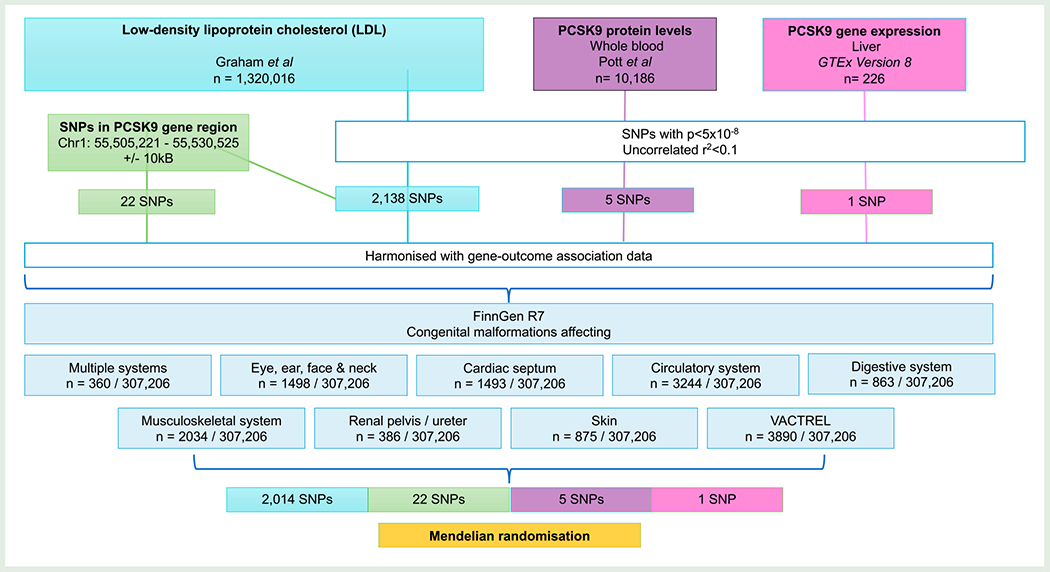
Study flowchart outlining study design. SNP, single-nucleotide polymorphism, PCSK9, proprotein convertase subtilisin–kexin type 9.

Genetic association estimates for congenital malformations were extracted from FinnGen Round 7,^19^ for the outcomes of congenital malformations affecting multiple systems (*n* cases = 360, *n* controls = 307 206), of the eye, ear, face and neck (*n* cases = 1498, *n* controls = 307 206), of the cardiac septum (*n* cases = 1493, *n* controls = 307 206), of the circulatory system (*n* case = 3244, *n* controls = 307 206), of the digestive system (*n* case = 833, *n* controls = 307 206), of the musculoskeletal system (*n* case = 2034, *n* controls = 307 206), of the renal pelvis and ureter (*n* case = 386, *n* controls = 307 206), of the skin (*n* case = 876, *n* controls = 307 206), and the vertebral, anorectal, cardiovascular, tracheo-esophageal, renal, and limb (VACTERL) association (*n* case = 3890, *n* controls = 307 206). Cohort numbers and International Classification of Disease (ICD) codes utilized for outcome definitions are reported in Table 1 and the study design is summarized in Figure 1.

### Statistical analysis

Inverse-variance weighted models were used for primary analysis^20^ using the Mendelianrandomization^21^ package in R. Bayesian tests for genetic colocalization^22^ were performed to investigate the posterior probability that exposure-outcome pairs share causal variants for LDL and congenital malformation risk within the *PCSK9* gene region.

For the primary analyses, an expected 5% false discovery rate (FDR) was controlled for using Benjamini-Hochberg correction of *P*-values. Results for the primary analyses are presented as odds ratio (OR) and 95% confidence intervals (95%CI) for every 1-standard deviation (SD) lower genetically predicted LDL, and FDR-adjusted *P*-values, with FDR-adjusted *P* < 0.05 considered statistically significant in the primary analysis, and nominal *P* < 0.05 considered statistically significant in the replication analyses and the further analyses using PCSK9 pQTLs and eQTLs. Results for PCSK9 pQTLs are presented as OR and 95%CI per unit lower normalized PCSK9 protein level, and results for *PCSK9* eQTLs per transcript per million (TPM) lower *PCSK9* gene expression.

### Instrumental variable assumptions

A number of additional sensitivity analyses were carried out to evaluate the instrumental variable assumptions. The instrumental variable assumptions state that for the results of MR analysis to be valid, the genetic variants must satisfy three key conditions:

1. The variants are able to predict the exposure.
2. There are no common causes of the genetic variant and the outcome.
3. The variant only influences the outcome through the exposure, and not directly or through alternative phenotypes.

The first assumption can be formally evaluated through the calculation of combined instrument F-statistics. In this study, these were calculated using the following formula:

$$F=\frac{\left(n-k-1\right)}{k}\frac{\left(R^{2}\right)}{\left(1-R^{2}\right)}$$

where *R*^2^ is the variance explained by the SNPs, *n* is the number of participants in the study, and *k* is the number of SNPs. The *R*^2^ was calculated as the sum of single-nucleotide polymorphism (SNP)-wise *R*^2^ of instruments, which is calculated as follows:

$$R^{2}=\frac{F}{\left(N-2+F\right)}\;\text{with}\;F={\left(\frac{\beta }{SE(\beta )}\right)}^{2}$$

where β represents the effect size of the genetic variant per additional effect allele, and SE(β) represents the standard error of β.

The second assumption cannot be formally tested, but was mitigated through the use of data sources for gene-exposure and gene-outcome association data from studies that included only European ancestry populations, to limit the potential for confounding from population stratification.

The third assumption was tested through sensitivity analyses using weighted median MR^23^ and MR-Egger.^24^ The weighted median method can provide consistent estimates assuming at least half the weight is derived from valid SNPs.^23^ The MR-Egger method can be used to identify the presence of directional pleiotropy under a weaker assumption that the instrument strength is independent of direct effects (InSIDE assumption).^24^

The third assumption was tested further to confirm that the genetic instruments used for MR analysis were valid by performing a phenome-wide scan. Phenome-wide scanning was performed for all traits associated with the SNPs that were used as instrumental variables within this study to proxy the effects of modifying the following:

- LDL cholesterol levels via PCSK9
- PCSK9 protein levels in whole blood (pQTL)
- PCSK9 gene expression in whole blood (eQTL)
- PCSK9 gene expression in liver (eQTL)
- LDL cholesterol levels overall

## Results

**Figure 2:**
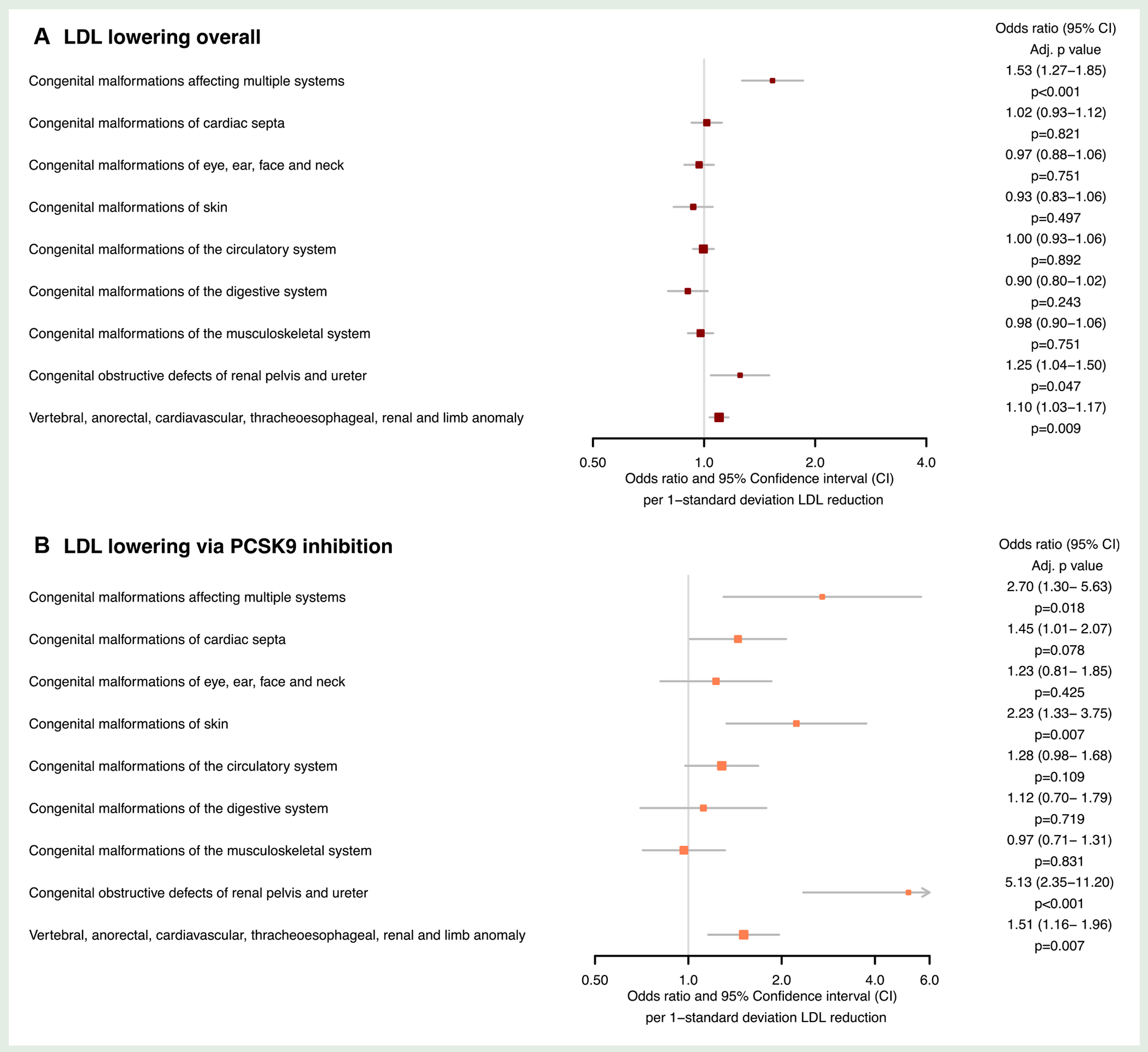
Forest plots displaying the Mendelian randomization estimates for the association between genetically predicted low-density lipoprotein-lowering: (*A*) by any means (*B*) via the proprotein convertase subtilisin–kexin type 9 (*PCSK9*) drug-target, with congenital malformations.

Genetically proxied fetal LDL-lowering overall was associated with higher odds of the VACTREL association [OR 1.10 (1.03–1.17) FDR-adjusted *P* = 0.009], malformations affecting multiple systems [OR 1.53 (1.27–1.85), FDR-adjusted *P* = 9.88 × 10^−5^] and obstructive defects of the renal pelvis and ureter [OR 1.25 (1.04–1.50), FDR-adjusted *P* = 0.047], as shown in Figure 2A. Sensitivity analyses did not identify potential directional pleiotropy (all MR-Egger intercept *P* > 0.05; Table 2). The combined F-statistic for LDL instruments was 294.24, as reported in Supplementary material online, Table S5.

Genetically proxied fetal LDL-lowering via *PCSK9* was associated with malformations affecting multiple systems [OR 2.70 (1.30–5.63), FDR-adjusted *P* = 0.018], malformations of the skin [OR 2.23 (1.33–3.75), FDR-adjusted *P* = 0.007], obstructive defects of the renal pelvis and ureter [OR 5.13 (2.35–11.20), FDR-adjusted *P* = 3.64 × 10^−4^], and the VACTERL association [OR 1.51 (1.16– 1.96), FDR-adjusted *P* = 0.007], as shown in Figure 2B. Sensitivity analyses identified directional pleiotropy in the association with malformations of the renal pelvis and ureter (MR-Egger intercept *P* = 0.001). No evidence of directional pleiotropy was identified for the other outcomes (all MR-Egger intercept *P* > 0.05) as reported in Table 2. The combined F-statistic for LDL via PCSK9 was 750.33, as reported in Supplementary material online, Table S5.

Colocalization analyses revealed weak evidence of shared causal variants for LDL in the *PCSK9* region with malformations of the skin (H4 = 55.75%; H3 = 1.32%). The results were inconclusive for malformations affecting multiple systems (H1= 79.71%, H3 = 2.54%, H4 = 17.74%), obstructive defects of the renal pelvis and ureter (H1 = 84.17%, H3 = 4.60%, H4 = 11.23%) and the VACTERL association (H2 = 79.32%, H3 = 5.97%, H4 = 14.71%). Full results of *PCSK9* colocalization analyses are presented in Supplementary material online, Table S6.

**Figure 3:**
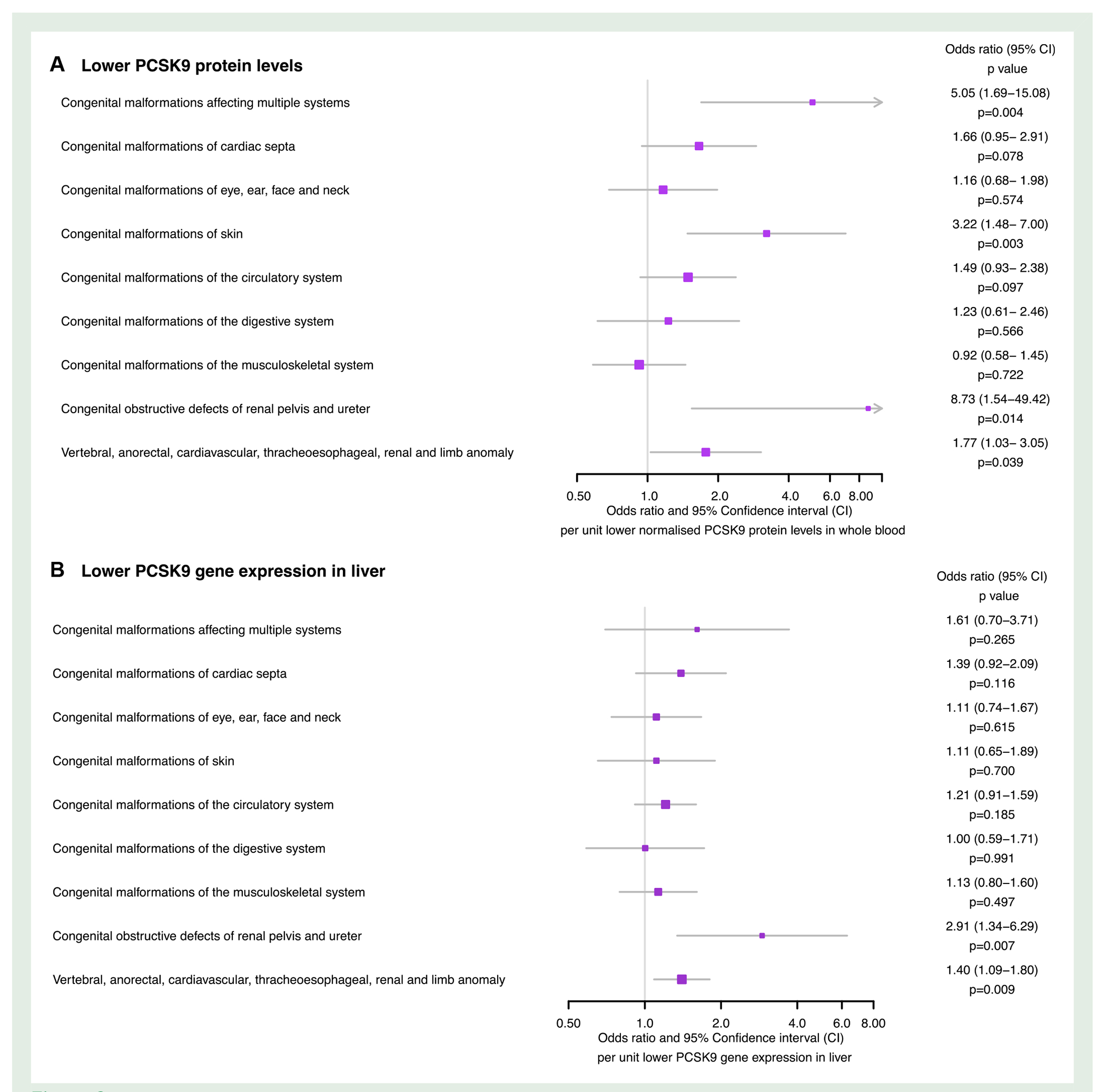
Forest plots displaying the Mendelian randomization estimates for the association of (*A*) lower genetically predicted circulating proprotein convertase subtilisin–kexin type 9 (PCSK9) levels (*B*) lower genetically predicted *PCSK9* gene expression in the liver.

Consistent with the PCSK9 drug-target MR findings, a unit lower normalized genetically predicted PCSK9 protein level was associated with a greater risk of congenital malformations affecting multiple systems [OR 5.05 (1.69–15.08), *P* = 0.004], the skin [OR 3.22 (1.48–7.00), *P* = 0.003], the renal pelvis and ureter [OR 8.73 (1.54—49.42), *P* = 0.014] and the VACTERL association [OR 1.77 (1.03–3.05), *P* = 0.039], as displayed in Figure 3A. Sensitivity analyses did not identify evidence of directional pleiotropy (all MR-Egger intercept *P* > 0.05), as reported in Table 2. The combined F-statistic for PCSK9 pQTL instruments was 18.90, as reported in Supplementary material online, Table S5.

Lower genetically predicted PCSK9 gene expression in the liver was associated with malformations of the renal pelvis and ureter [OR 2.91 (1.34–6.29), *P* = 0.007] as well as the VACTREL association [OR 1.40 (1.09–1.80), *P* = 0.009], as reported in Figure 3B. These results might be biased toward the observational estimate due to weak instruments, as the combined F-statistic for *PCSK9* liver eQTL instruments was 4.91, as reported in Supplementary material online, Table S5.

UK Biobank replication analyses yielded findings broadly consistent with the main analyses, as presented in Table 3, though statistical significance was lost for the association between LDL-lowering overall and congenital malformations of the renal pelvis and ureter [OR 1.25 (0.99–1.58), *P* = 0.059] as well as the VACTREL association [OR 1.08 (1.00–1.16), *P* = 0.066] despite association estimates consistent in magnitude and direction.

Phenome-wide scanning for SNPs instrumenting LDL levels via PCSK9, PCSK9 protein levels in whole blood (pQTL), PCSK9 gene expression in whole blood (eQTL), PCSK9 gene expression in liver (eQTL), and SNPs instrumenting LDL cholesterol levels overall identified 7119 phenotypic traits in total, reported in Supplementary material online, Table S7.

## Discussion

This study leverages genetic variants associated with LDL levels in the *PCSK9* region, as well as variants associated with actual PCSK9 protein levels and gene expression in the liver, to explore potential fetal effects of administration of a *PCSK9* inhibitor capable of crossing the placenta during pregnancy. Within this framework, the results support an association between fetal LDL-lowering via *PCSK9*-inhibition and multiple types of congenital malformations. The results, therefore, corroborate current manufacturer recommendations against the use of *PCSK9*-inhibition during pregnancy.

The results of this study extend current knowledge regarding the importance of LDL in pregnancy. Fetal cholesterol metabolism is vital in placentation and early embryogenesis,^25^ and *PCSK9* plays a key role in its regulation.^7^ In syndromes characterized by extremely low LDL levels, such as SLOS, a panoply of malformations occur.^8^ Additionally, previous studies have described an association of lower *PCSK9* levels with neural tube defects.^26^ There are many potential mechanisms underlying these associations supported by the results of our study. Of central importance, cholesterol plays a major role in the normal maturation and signalling of hedgehog (Hh) proteins, a family of proteins that are critical for pattern formation during embryonic development.^27^ Impaired Hh signalling due to low cholesterol levels has been suggested to underlie at least some of the malformations that are typical of SLOS, including holoprosencephaly, agenesis of the corpus callosum, and postaxial polydactyly.^28^ Supporting this, Cooper et al,^28^ previously demonstrated significant compromise in Hh signal in cells from mouse models of SLOS and lathosterolosis, but also in normal cells that were pharmacologically depleted of cholesterol. Thus, previous studies investigating the pathophysiology of SLOS support the notion that cholesterol deficiency plays a role in altering membrane properties and promoting congenital malformations, in addition to the increased levels of dehydrocholesterol, a sterol precursor that is elevated in SLOS. While the latter mechanism is expected to be unique to the inborn error of metabolism that characterizes SLOS and bears no relevance to PCSK9 signalling, the cholesterol deficiency mechanism is likely relevant in the associations of low PCSK9 activity, be due to the R46L PCSK9 mutation or administration of a PCSK9 inhibitor that crosses the placenta, with congenital malformations.

An investigational *in vivo* clustered regularly interspaced short palindromic repeats (CRISPR) base editing therapy, VERVE-101 is currently under development^29^ with the aim to permanently hinder hepatic *PCSK9* production by altering a single DNA base in the *PCSK9* gene. If this approach proves effective, this might be a theoretically safer option for women planning to conceive, as long as pregnancy occurs after the ‘active’ delivery phase. Unfortunately, this cannot be inferred with confidence, as this study does not exclude that potential ‘indirect’ effects of lowering maternal LDL might occur on the fetus. However, this therapy would not be expected to interfere with fetal LDL metabolism, given that pre-clinical data in primates has promisingly suggested that *PCSK9* gene editing in liver (i.e. non-germline) cells is not heritable, and would, therefore, not be expected to exhibit the associations described in this study.^29^ Considering the results of this study, we highlight the importance of thorough investigation of the potential reproductive safety of this novel therapy, to avoid its contraindication due to insufficient data which contributes to inequity of care for women during reproductive years.

This study has important clinical implications. First, in practical terms, the results do not suggest that any change should be made to the current recommendations to avoid these drugs in pregnancy. Second, it follows that reproductive wishes should be discussed with women of reproductive age taking *PCSK9* inhibitors. Contraception advice and appropriate pre-conception planning are warranted. At present, no guidelines or consensus statements exist to provide advice on the appropriate timing to stop *PCSK9*-inhibitors relative to attempts to conceive. Despite the recognition that limited data is available, these would be useful to guide advice for the pre-conception stage and to guide conversations with women who conceive accidentally during therapy. From a research perspective, the findings call for curation of a registry to monitor outcomes among individuals with inadvertent PCSK9i exposure in pregnancy to see if any signals are recapitulated. Finally, clinicians must be aware of the importance of meticulous care for women of reproductive age on *PCSK9* inhibitors, because these safety concerns risk widening existing sex-based disparities in cardiovascular care.^30^

## Limitations

There are a number of limitations to discuss. First, we unfortunately cannot ascertain in which trimester LDL-lowering was highest risk for malformations, and it is plausible that risk may differ throughout different stages of pregnancy. Second, as mentioned, we cannot exclude additional effects that might relate to indirect influence of maternal LDL-lowering. Third, the instruments for the analysis of PCSK9 gene expression in both the liver were weak (F-statistics <10) and might therefore be biased towards observational estimates. Once larger data sources are available when further releases of GTEx data are available, the analyses should be repeated to ensure the findings for these exposures are not influenced by weak instrument bias. However, it is important to note that weak instrument bias in a two-sample MR setting using nonoverlapping cohorts typically results in estimates that are biased toward the null, it would therefore not be expected to exaggerate inferences made in this study for these instruments. Finally, horizontal pleiotropy can limit MR investigations and if present, may result in violation of the third instrumental variable assumption whereby genetic variants must only influence the outcome through the exposure, and not directly or through alternative phenotypes. In order to test this, we performed sensitivity analyses using robust methods including weighted median and MR Egger approaches which did not identify the presence of directional pleiotropy. To further maintain confidence in our results, we performed phenome-wide scanning to identify phenotypic traits associated with the genetic variants selected as instrumental variables to identify any alternative pathways between the exposure and outcome unrelated to the biomarker of interest. These demonstrated that the SNPs instrumenting LDLviaPCSK9 and all PCSK9 pQTL and eQTLs were mostly associated with cholesterol and cholesterol-related traits, further supporting the absence of horizontal pleiotropy in these MR analyses. The range of associations with SNPs instrumenting LDL overall was much broader, so the existence of pleiotropy cannot be fully excluded from these data. However, evidence to refute this possibility is provided by the sensitivity analyses using robust methods failing to detect directional pleiotropy. Taken together, this suggests that if pleiotropy exists, it is balanced and would not be expected to influence the direction or magnitude of the association of genetically predicted LDL with the outcomes.

## Conclusion

In conclusion, the results of this study support current manufacturer recommendations to avoid the use of *PCSK9*-inhibition during pregnancy. By extension, it is prudent for physicians looking after women of reproductive age on PCSK9 inhibitors to counsel patients regarding contraception and to encourage planned pregnancy with appropriate pre-conception care.

## Supplementary Material

Supplementary material, Tables S1-S6

Supplementary material, Table S7

## Figures and Tables

**Table 1:** Data sources for exposures and outcomes with international statistical classification of diseases and related health problems 10th revision (ICD-10) codes

Exposures					

Drug target	Data source	Gene	Chromosome: base pair position (excluding 10 kB window)	*n*_uncorrelated SNPs_ (*r*^2^ < 0.1)	

LDL	Graham *et al*	—	—	2014	
LDL via PCSK9	Graham *et al*	*PCSK9*	Chr1: 55 505 221–55 530 525	22	
PCSK9 protein levels in whole blood	Graham *et al*	*PCSK9*	Chr1: 55 505 221–55 530 525	5	
PCSK9 gene expression in liver	GTEx 8	*PCSK9*	Chr1: 55 505 221–55 530 525	1	
PCSK9 gene expression in whole blood	GTEx 8	*PCSK9*	Chr1: 55 505 221–55 530 525	2	

*Outcomes*					

Phenotype		Data source	ICD-10 codes	Cases	Control

Congenital malformations affecting multiple systems		FinnGen R7	Q87	360	307 206
Congenital malformations of eye, ear, face, and neck		FinnGen R7	Q10-Q18	1498	307 656
Congenital malformations of the cardiac septum		FinnGen R7	Q21	1493	305 910
Congenital malformations of the circulatory system		FinnGen R7	Q20-Q28	3244	305 910
Congenital malformations of the digestive system		FinnGen R7	Q38-Q45	863	308 291
Congenital malformations of the musculoskeletal system		FinnGen R7	Q65-Q79	2034	307 120
Congenital malformations of the skin		FinnGen R7	Q82	875	307 206
Congenital obstructive defects of renal pelvis and ureter		FinnGen R7	Q62	386	307 923
Vertebral, anorectal, cardiovascular, tracheo-esophageal, renal, and limb anomaly		FinnGen R7	Q8726|Q76| Q675| Q42|Q43[5–9]| Q20|Q21|Q22| Q23|Q24|Q25| Q26[1–4]|Q39| Q6[0–4]| Q69[0–1]| Q70[014]|Q71	3890	305 264

*Sensitivity analysis: Exposures*					

Drug target	Data source	Gene	Chromosome and base pair position (excludes 10 kB window)	#Uncorrelated SNPs, *r*^2^ < 0.1	

LDL	Neale lab	—	—	941	
LDL via PCSK9	Neale lab	*PCSK9*	Chr1: 55 505 221–55 530 525	16	

SNP, single-nucleotide polymorphism; LDL, low-density lipoprotein; PCSK9, proprotein convertase subtilisin–kexin type 9.

**Table 2:** Mendelian randomization sensitivity analyses for the genetically predicted LDL-c lowering overall, through PCSK9 drug targets, and for PCSK9 protein levels in whole blood

Exposure	Outcome	Method	Beta	Standard error	*P*-value
LDL-lowering overall (1-SD)	Congenital malformations affecting multiple systems	Weighted median	0.582	0.177	0.001
		MR Egger	0.119	0.044	0.007
		intercept	−0.003	0.004	0.370
	Congenital malformations of cardiac septa	Weighted median	−0.054	0.085	0.522
		MR Egger	−0.004	0.070	0.960
		intercept	0.001	0.002	0.694
	Congenital malformations of eye, ear, face, and neck	Weighted median	−0.058	0.082	0.475
		MR Egger	−0.028	0.068	0.677
		intercept	0.000	0.002	0.960
	Congenital malformations of skin	Weighted median	0.069	0.115	0.548
		MR Egger	0.520	0.142	0.000
		intercept	−0.001	0.002	0.683
	Congenital malformations of the circulatory system	Weighted median	−0.048	0.060	0.422
		MR Egger	0.006	0.048	0.900
		intercept	0.000	0.001	0.765
	Congenital malformations of the digestive system	Weighted median	−0.079	0.114	0.486
		MR Egger	−0.110	0.092	0.232
		intercept	0.000	0.002	0.895
	Congenital malformations of the musculoskeletal system	Weighted median	0.030	0.073	0.683
		MR Egger	−0.075	0.059	0.205
		intercept	0.002	0.001	0.221
	Congenital obstructive defects of renal pelvis and ureter	Weighted median	0.038	0.159	0.810
		MR Egger	0.409	0.136	0.003
		intercept	−0.006	0.003	0.062
	Vertebral, anorectal, cardiovascular, tracheo-esophageal, renal, and limb anomaly	Weighted median	0.058	0.053	0.275
		MR-Egger	−0.041	0.091	0.654
		intercept	−0.001	0.001	0.432
LDL-lowering via PCSK9-inhibition (1-SD)	Congenital malformations affecting multiple systems	Weighted median	1.017	0.450	0.024
		MR Egger	0.238	0.182	0.204
		intercept	0.001	0.044	0.986
	Congenital malformations of cardiac septa	Weighted median	0.250	0.221	0.258
		MR Egger	0.191	0.251	0.456
		intercept	0.022	0.021	0.315
	Congenital malformations of eye, ear, face, and neck	Weighted median	0.036	0.208	0.862
		MR Egger	−0.122	0.278	0.664
		intercept	0.040	0.024	0.102
	Congenital malformations of skin	Weighted median	0.772	0.254	0.002
		MR Egger	0.988	0.518	0.070
		intercept	0.003	0.033	0.919
	Congenital malformations of the circulatory system	Weighted median	0.219	0.142	0.123
		MR Egger	0.215	0.195	0.284
		intercept	0.004	0.017	0.805
	Congenital malformations of the digestive system	Weighted median	0.018	0.278	0.948
		MR Egger	−0.115	0.329	0.730
		intercept	0.028	0.028	0.327
	Congenital malformations of the musculoskeletal system	Weighted median	−0.073	0.180	0.686
		MR Egger	−0.060	0.214	0.782
		intercept	0.003	0.018	0.856
	Congenital obstructive defects of renal pelvis and ureter	Weighted median	0.912	0.444	0.040
		MR Egger	0.490	0.495	0.333
		intercept	0.138	0.042	0.003
	Vertebral, anorectal, cardiovascular, tracheo-esophageal, renal, and limb anomaly	Weighted median	0.285	0.137	0.037
		MR-Egger	0.778	0.363	0.044
		intercept	0.021	0.015	0.183
Lower PCSK9 protein levels in whole blood (normalized protein expression units)	Congenital malformations affecting multiple systems	Weighted median	1.544	0.613	0.012
		MR Egger	1.359	0.846	0.207
		intercept	0.027	0.066	0.709
	Congenital malformations of cardiac septa	Weighted median	0.368	0.312	0.238
		MR Egger	0.359	0.487	0.515
		intercept	0.015	0.038	0.716
	Congenital malformations of eye, ear, face, and neck	Weighted median	0.057	0.313	0.855
		MR Egger	−0.094	0.410	0.833
		intercept	0.026	0.032	0.481
	Congenital malformations of skin	Weighted median	1.124	0.380	0.003
		MR Egger	1.128	0.670	0.191
		intercept	0.005	0.057	0.939
	Congenital malformations of the circulatory system	Weighted median	0.307	0.222	0.168
		MR Egger	0.395	0.419	0.415
		intercept	0.000	0.033	0.993
	Congenital malformations of the digestive system	Weighted median	0.029	0.407	0.944
		MR Egger	−0.111	0.537	0.849
		intercept	0.033	0.042	0.491
	Congenital malformations of the musculoskeletal system	Weighted median	−0.101	0.260	0.698
		MR Egger	−0.157	0.350	0.684
		intercept	0.008	0.028	0.795
	Congenital obstructive defects of renal pelvis and ureter	Weighted median	1.669	0.634	0.008
		MR Egger	0.548	0.963	0.609
		intercept	0.165	0.074	0.113
	Vertebral, anorectal, cardiovascular, tracheo-esophageal, renal, and limb anomaly	Weighted median	0.440	0.191	0.021
		MR-Egger	0.407	0.468	0.448
		intercept	0.017	0.037	0.671

Sensitivity analyses could not be carried out for analyses with exposures of PCSK9 gene expression (in liver and whole blood) as <3 SNPs were available.

**Table 3:** Results of the UK biobank replication analyses: tabulated Mendelian randomization summary estimates for the genetic association between low-density lipoprotein-lowering generally, and via the PCSK9 drug-target

Exposure	n_SNP_	Outcome	Odds ratio	Lower 95% CI	Upper 95% CI	*P*-value
LDL-lowering overall (1-SD)	607	Congenital malformations of cardiac septa	0.96	0.85	1.08	0.478
	607	Congenital malformations of the circulatory system	0.99	0.91	1.07	0.737
	607	Congenital malformations of the musculoskeletal system	1.00	0.91	1.10	0.966
	607	Congenital malformations of eye, ear, face, and neck	0.99	0.88	1.11	0.846
	607	Congenital obstructive defects of renal pelvis and ureter	1.25	0.99	1.58	0.059
	607	Congenital malformations of the digestive system	0.94	0.81	1.11	0.478
	607	Congenital malformations of skin	0.91	0.78	1.07	0.256
	607	Congenital malformations affecting multiple systems	1.58	1.25	2.01	0.000
	607	Vertebral, anorectal, cardiovascular, tracheo-esophageal, renal, and limb anomaly	1.08	1.00	1.16	0.066
LDL-lowering via PCSK9-inhibition (1-SD)	11	Congenital malformations of cardiac septa	1.44	0.89	2.32	0.133
	11	Congenital malformations of the circulatory system	1.23	0.89	1.69	0.217
	11	Congenital malformations of the musculoskeletal system	0.97	0.65	1.46	0.882
	11	Congenital malformations of eye, ear, face, and neck	1.24	0.77	1.99	0.374
	11	Congenital obstructive defects of renal pelvis and ureter	4.52	1.78	11.51	0.002
	11	Congenital malformations of the digestive system	1.11	0.59	2.06	0.747
	11	Congenital malformations of skin	3.00	1.49	6.03	0.002
	11	Congenital malformations affecting multiple systems	4.48	1.68	11.89	0.003
	11	Vertebral, anorectal, cardiovascular, tracheo-esophageal, renal, and limb anomaly	1.46	1.08	1.96	0.012

All data is from Neale Lab’s second release within UK Biobank, *n* = 469 897 (http://www.nealelab.is/uk-biobank/).

## Data Availability

The data underlying this article were derived from sources in the public domain and these data sources have been appropriately cited within the manuscript.
