# Supplementary material for: Genetically proxied low-density lipoprotein cholesterol lowering via PCSK9-inhibitor drug targets and risk of congenital malformations
Source: Eur J Prev Cardiol. Author manuscript; Available in PMC 2024 Jun 3. (PMC11144467; doi:10.1093/eurjpc/zwad402)
Supplement: Supplementary material, Tables S1-S6 [file NIHMS1982876-supplement-Supplementary_material__Tables_S1-S6.docx]

# Supplemental Material

**Genetically-proxied low-density lipoprotein cholesterol lowering via PCSK9-inhibitor drug targets and risk of congenital malformations**

Maddalena Ardissino MBBS^1-3^, Eric A. W. Slob PhD^4-6^, Rohin K. Reddy MBBS^1^, Alec P. Morley BSc^3^, Art Schuermans BSc^6-8^, Phoebe Hill MBBS^9^ Catherine Williamson MD^10^, Michael C Honigberg MD MPP^6,7,13^, Antonio de Marvao PhD^11,12^, Fu Siong Ng PhD^1^

^1^National Heart and Lung Institute, Imperial College London, UK

^2^Department of Medicine, School of Clinical Medicine, University of Cambridge, UK

^3^MRC Biostatistics Unit, School of Clinical Medicine, University of Cambridge, UK

^4^Department of Applied Economics, Erasmus School of Economics, Erasmus University Rotterdam, The Netherlands

^5^Erasmus University Rotterdam Institute for Behavior and Biology, Erasmus University Rotterdam, The Netherlands

^6^Program in Medical and Population Genetics and Cardiovascular Disease Initiative, Broad Institute of Harvard and MIT, Cambridge, MA, USA

^7^Cardiovascular Research Center and Center for Genomic Medicine, Massachusetts General Hospital, Harvard Medical School, Boston, MA, USA

^8^Department of Cardiovascular Sciences, KU Leuven, Leuven, Belgium

^9^Manchester Royal Infirmary, Manchester University NHS Foundation Trust, Manchester, UK

^10^Department of Women and Children's Health, King's College London, London, United Kingdom

^11^British Heart Foundation Centre of Research Excellence, School of Cardiovascular Medicine and Sciences, King's College London, London, United Kingdom.

^12^Medical Research Council, London Institute of Medical Sciences, Imperial College London UK

^13^Cardiology Division, Department of Medicine, Massachusetts General Hospital, Boston, MA, USA

**Contents**

**Table S1.** Uncorrelated (r2<0.1) instrumental variants at p<5x10^-8 for 1-SD lower LDL.

**Table S2.** Uncorrelated (r2<0.1) instrumental variants at p<5x10^-8 for 1-SD lower LDL in the PCSK9 gene region.

**Table S3.** Uncorrelated (r2<0.1) instrumental variants at p<5x10^-8 for lower normalized circulating PCSK9 protein levels.

**Table S4.** Uncorrelated (r2<0.1) instrumental variants at p<5x10^-8 for lower PCSK9 gene expression in the liver.

**Table S5.** Proportion of variance in phenotype explained by instrumental variants, number of instrumental variants, number of participants in genome-wide association study, and combined F-statistics for instruments.

**Table S6.** Posterior probabilities for the PCSK9 gene region obtained via Bayesian tests for genetic colocalization.

**Table S1.** Uncorrelated (r2<0.1) instrumental variants at p<5x10^-8 for 1-SD lower LDL. EAF = Effect allele frequency; Beta = Coefficient of effect allele; SNP = single-nucleotide polymorphism; SE = Standard error

| SNP | Beta | SE | P-value | EAF | Effect allele | Other allele |
| --- | --- | --- | --- | --- | --- | --- |
| rs10000446 | 0.0164443 | 0.00286705 | 9.72E-09 | 0.0644 | T | C |
| rs1001935 | -0.0247673 | 0.00254383 | 2.11E-22 | 0.0798 | A | G |
| rs10025454 | 0.00922286 | 0.0014832 | 5.03E-10 | 0.33 | A | T |
| rs10044005 | 0.0120001 | 0.00140457 | 1.30E-17 | 0.437 | G | A |
| rs10069709 | -0.0385243 | 0.00166446 | 1.62E-118 | 0.235 | T | A |
| rs10069831 | 0.00948576 | 0.00145025 | 6.12E-11 | 0.379 | A | T |
| rs1007938 | -0.0082863 | 0.00142068 | 5.46E-09 | 0.406 | G | A |
| rs10079961 | 0.019674 | 0.00242251 | 4.61E-16 | 0.0941 | T | G |
| rs1010810 | 0.0131259 | 0.00217345 | 1.55E-09 | 0.116 | A | G |
| rs1011158 | 0.0102533 | 0.00141527 | 4.33E-13 | 0.491 | A | G |
| rs10111761 | 0.0144647 | 0.0021251 | 9.99E-12 | 0.12 | C | T |
| rs1011390 | 0.00874828 | 0.00144751 | 1.51E-09 | 0.387 | T | C |
| rs1014283 | 0.0136301 | 0.00179818 | 3.46E-14 | 0.183 | A | C |
| rs10146358 | 0.0186299 | 0.00259049 | 6.40E-13 | 0.0784 | C | G |
| rs10152515 | -0.0099147 | 0.00155842 | 1.99E-10 | 0.296 | A | C |
| rs10159261 | -0.0086843 | 0.00149625 | 6.47E-09 | 0.313 | T | G |
| rs10163491 | -0.010289 | 0.00149499 | 5.89E-12 | 0.339 | A | G |
| rs10171868 | -0.013042 | 0.00176003 | 1.26E-13 | 0.2 | C | G |
| rs10181342 | 0.0209537 | 0.00171055 | 1.69E-34 | 0.212 | G | A |
| rs10184004 | 0.0104874 | 0.00140997 | 1.02E-13 | 0.413 | T | C |
| rs10184673 | -0.0220445 | 0.00142603 | 6.60E-54 | 0.405 | G | A |
| rs10185855 | 0.0129858 | 0.0014352 | 1.46E-19 | 0.361 | G | A |
| rs10186086 | 0.0215884 | 0.00176773 | 2.67E-34 | 0.194 | A | T |
| rs10198258 | 0.00883949 | 0.00150948 | 4.74E-09 | 0.305 | C | T |
| rs10206764 | -0.010666 | 0.00143142 | 9.24E-14 | 0.407 | C | T |
| rs10214652 | -0.0230663 | 0.00328101 | 2.06E-12 | 0.0545 | A | G |
| rs10221914 | -0.0295996 | 0.00448166 | 3.99E-11 | 0.0279 | T | C |
| rs10248717 | -0.0125877 | 0.00153365 | 2.26E-16 | 0.309 | G | A |
| rs10252404 | -0.0168293 | 0.00196088 | 9.28E-18 | 0.149 | A | C |
| rs10272002 | 0.0223057 | 0.00171399 | 1.02E-38 | 0.21 | G | A |
| rs1031101 | -0.0120767 | 0.00197776 | 1.02E-09 | 0.142 | G | A |
| rs1037117 | 0.0099077 | 0.00162522 | 1.09E-09 | 0.253 | A | G |
| rs10402752 | -0.0101423 | 0.00172089 | 3.78E-09 | 0.264 | C | T |
| rs10404677 | 0.0452465 | 0.00679769 | 2.81E-11 | 0.0117 | A | G |
| rs10406304 | -0.0124536 | 0.0015745 | 2.58E-15 | 0.28 | T | G |
| rs10410651 | 0.0337365 | 0.00158393 | 1.15E-100 | 0.262 | C | T |
| rs10414919 | 0.00877174 | 0.00155548 | 1.71E-08 | 0.28 | A | G |
| rs10415392 | -0.0254578 | 0.00234693 | 2.05E-27 | 0.102 | T | C |
| rs10419086 | 0.0514735 | 0.00341477 | 2.41E-51 | 0.0451 | G | A |
| rs10419906 | -0.0157871 | 0.00280899 | 1.91E-08 | 0.0717 | G | C |
| rs1042023 | -0.135922 | 0.00675017 | 3.56E-90 | 0.0116 | C | G |
| rs10420325 | -0.0156809 | 0.00225538 | 3.58E-12 | 0.35 | A | T |
| rs10420562 | -0.039244 | 0.00254521 | 1.22E-53 | 0.083 | T | C |
| rs10421830 | -0.0227787 | 0.00230334 | 4.63E-23 | 0.102 | C | T |
| rs10422122 | 0.0141161 | 0.00157093 | 2.57E-19 | 0.304 | T | C |
| rs10422253 | -0.0137209 | 0.00172993 | 2.17E-15 | 0.221 | A | G |
| rs10423802 | -0.0118284 | 0.00170574 | 4.08E-12 | 0.209 | T | C |
| rs10426801 | 0.0348531 | 0.00315867 | 2.62E-28 | 0.0553 | T | A |
| rs10438978 | 0.0177685 | 0.0018146 | 1.22E-22 | 0.18 | T | C |
| rs1044573 | -0.0102945 | 0.00141172 | 3.05E-13 | 0.495 | G | A |
| rs10456852 | 0.0159823 | 0.00215527 | 1.21E-13 | 0.124 | T | C |
| rs1045873 | -0.0079624 | 0.00143734 | 3.03E-08 | 0.385 | C | A |
| rs10462958 | -0.0127651 | 0.00148285 | 7.40E-18 | 0.38 | T | C |
| rs10483285 | -0.0168788 | 0.00295658 | 1.14E-08 | 0.0594 | A | C |
| rs10484494 | 0.0189165 | 0.00284141 | 2.79E-11 | 0.068 | A | G |
| rs10485678 | -0.0204027 | 0.00300953 | 1.21E-11 | 0.0588 | T | C |
| rs10495716 | -0.0148864 | 0.00159272 | 9.06E-21 | 0.31 | A | C |
| rs1050541 | -0.0086772 | 0.00146874 | 3.46E-09 | 0.45 | T | G |
| rs1052248 | -0.0149492 | 0.00162462 | 3.52E-20 | 0.262 | A | T |
| rs10757273 | 0.0140171 | 0.00154632 | 1.25E-19 | 0.443 | A | C |
| rs10761750 | -0.0141123 | 0.00139415 | 4.39E-24 | 0.483 | A | G |
| rs10790519 | -0.015594 | 0.00142746 | 8.82E-28 | 0.385 | C | T |
| rs10807832 | -0.0112923 | 0.00203439 | 2.85E-08 | 0.134 | T | A |
| rs1081106 | -0.0538241 | 0.00264362 | 3.79E-92 | 0.0837 | C | T |
| rs10832956 | 0.0208132 | 0.00155842 | 1.10E-40 | 0.272 | T | C |
| rs10843390 | 0.0097591 | 0.00154733 | 2.84E-10 | 0.28 | T | C |
| rs10850078 | -0.0085926 | 0.00142151 | 1.50E-09 | 0.428 | G | A |
| rs10852932 | 0.00880558 | 0.00144556 | 1.12E-09 | 0.358 | T | G |
| rs10858093 | -0.0443277 | 0.00473215 | 7.44E-21 | 0.0265 | T | C |
| rs10883447 | -0.0106417 | 0.0014254 | 8.28E-14 | 0.407 | G | T |
| rs10888908 | 0.0509531 | 0.00256759 | 1.22E-87 | 0.0801 | T | C |
| rs10893483 | -0.0188146 | 0.0022122 | 1.82E-17 | 0.11 | G | A |
| rs10899485 | 0.0117891 | 0.00188189 | 3.74E-10 | 0.163 | C | T |
| rs10901256 | 0.02414 | 0.00159505 | 9.62E-52 | 0.26 | C | T |
| rs10903129 | 0.0258481 | 0.00138909 | 2.77E-77 | 0.452 | A | G |
| rs10907228 | 0.00906728 | 0.00150292 | 1.61E-09 | 0.41 | T | C |
| rs10910471 | 0.0180558 | 0.00295201 | 9.57E-10 | 0.0587 | T | C |
| rs10910481 | 0.0136491 | 0.00199107 | 7.12E-12 | 0.148 | C | T |
| rs10910489 | 0.0257745 | 0.00431638 | 2.35E-09 | 0.0316 | G | A |
| rs10910521 | -0.0164355 | 0.00144601 | 6.17E-30 | 0.415 | T | C |
| rs10910535 | -0.0098806 | 0.00173534 | 1.24E-08 | 0.265 | T | A |
| rs10912842 | -0.0087114 | 0.00158773 | 4.10E-08 | 0.342 | G | C |
| rs10928521 | -0.0107086 | 0.00169761 | 2.83E-10 | 0.227 | C | G |
| rs10946807 | 0.0177688 | 0.00145983 | 4.39E-34 | 0.352 | C | T |
| rs10946904 | -0.0116851 | 0.00157665 | 1.25E-13 | 0.268 | T | C |
| rs10953298 | 0.0159823 | 0.00162966 | 1.05E-22 | 0.243 | T | C |
| rs10956245 | -0.018835 | 0.00318308 | 3.27E-09 | 0.0541 | T | C |
| rs10957057 | 0.0141579 | 0.0022029 | 1.30E-10 | 0.116 | T | C |
| rs1095974 | 0.0144033 | 0.00240205 | 2.02E-09 | 0.0956 | G | C |
| rs11000443 | 0.0214412 | 0.00339256 | 2.61E-10 | 0.0434 | A | C |
| rs11022130 | -0.0102331 | 0.00165355 | 6.07E-10 | 0.251 | G | A |
| rs11023881 | 0.0085617 | 0.00141813 | 1.57E-09 | 0.395 | A | T |
| rs11038635 | 0.0278492 | 0.00288026 | 4.08E-22 | 0.0623 | A | G |
| rs11057840 | -0.0218994 | 0.00201288 | 1.44E-27 | 0.138 | C | A |
| rs11064842 | 0.00792855 | 0.00139768 | 1.41E-08 | 0.448 | T | C |
| rs1107894 | 0.00869001 | 0.001482 | 4.53E-09 | 0.346 | A | G |
| rs11080061 | 0.010114 | 0.00175208 | 7.81E-09 | 0.213 | A | C |
| rs11088186 | -0.0132969 | 0.00230806 | 8.36E-09 | 0.105 | C | T |
| rs11096689 | -0.049547 | 0.00152922 | 1.00E-200 | 0.292 | C | T |
| rs11101981 | -0.0123862 | 0.00150489 | 1.86E-16 | 0.35 | C | T |
| rs11102002 | 0.0327312 | 0.0030675 | 1.40E-26 | 0.0537 | T | C |
| rs11102709 | 0.00916926 | 0.00163375 | 2.00E-08 | 0.237 | C | G |
| rs11114053 | -0.0133322 | 0.0015206 | 1.82E-18 | 0.302 | T | C |
| rs11118606 | -0.0122744 | 0.00158708 | 1.04E-14 | 0.264 | G | A |
| rs111278137 | 0.109218 | 0.00524629 | 2.96E-96 | 0.0192 | A | G |
| rs111353455 | -0.0185571 | 0.00250322 | 1.23E-13 | 0.0834 | A | G |
| rs111386723 | 0.0301943 | 0.00488019 | 6.13E-10 | 0.0225 | G | C |
| rs111435864 | -0.042377 | 0.0034566 | 1.49E-34 | 0.0448 | C | T |
| rs111442610 | 0.0229154 | 0.00350113 | 5.94E-11 | 0.0516 | A | G |
| rs11144750 | 0.0115311 | 0.00188265 | 9.07E-10 | 0.166 | C | A |
| rs11153143 | 0.0144056 | 0.00212572 | 1.23E-11 | 0.126 | T | C |
| rs1115866 | -0.0097097 | 0.00144057 | 1.58E-11 | 0.412 | C | T |
| rs111622501 | 0.0307125 | 0.00464269 | 3.71E-11 | 0.0241 | T | C |
| rs11164788 | -0.014623 | 0.00183443 | 1.57E-15 | 0.175 | G | A |
| rs11164858 | -0.0080213 | 0.00143501 | 2.27E-08 | 0.402 | A | G |
| rs11166574 | -0.0106076 | 0.00166125 | 1.71E-10 | 0.234 | A | G |
| rs11167778 | -0.0218031 | 0.00225008 | 3.33E-22 | 0.107 | T | C |
| rs111686227 | -0.0453514 | 0.00711742 | 1.87E-10 | 0.0108 | A | G |
| rs11172134 | 0.0147456 | 0.00172701 | 1.36E-17 | 0.208 | A | T |
| rs111724091 | -0.0190172 | 0.00334046 | 1.25E-08 | 0.0537 | A | C |
| rs11175518 | -0.0254522 | 0.00282385 | 2.00E-19 | 0.0654 | C | T |
| rs111780744 | -0.0791719 | 0.00576809 | 7.11E-43 | 0.017 | T | C |
| rs111855557 | 0.0144546 | 0.00191637 | 4.60E-14 | 0.167 | A | G |
| rs111859423 | -0.0333129 | 0.00425439 | 4.87E-15 | 0.0316 | C | T |
| rs111865370 | 0.045647 | 0.00591869 | 1.24E-14 | 0.0154 | G | A |
| rs11187298 | -0.0106849 | 0.00144155 | 1.24E-13 | 0.382 | T | C |
| rs11187838 | 0.00827971 | 0.00139972 | 3.31E-09 | 0.433 | A | G |
| rs111918389 | -0.0367293 | 0.00398166 | 2.85E-20 | 0.0317 | C | T |
| rs111938177 | 0.043375 | 0.00653668 | 3.23E-11 | 0.0124 | T | C |
| rs111995966 | -0.0288672 | 0.00504542 | 1.06E-08 | 0.021 | G | T |
| rs112027066 | 0.11885 | 0.00538666 | 7.05E-108 | 0.0177 | T | C |
| rs11206464 | -0.0086663 | 0.0015462 | 2.08E-08 | 0.492 | C | T |
| rs11206498 | -0.010318 | 0.00146968 | 2.21E-12 | 0.342 | G | A |
| rs11206517 | -0.0888589 | 0.00371072 | 1.00E-126 | 0.0371 | G | T |
| rs112121639 | 0.041352 | 0.00724469 | 1.14E-08 | 0.0101 | A | G |
| rs112126568 | 0.0264865 | 0.00270973 | 1.45E-22 | 0.0748 | T | C |
| rs112129014 | 0.022129 | 0.00320064 | 4.71E-12 | 0.0509 | T | C |
| rs112136706 | -0.0383581 | 0.00686834 | 2.34E-08 | 0.0131 | A | G |
| rs112159161 | 0.200048 | 0.00590967 | 1.00E-200 | 0.0146 | T | C |
| rs112170089 | -0.0521114 | 0.00593457 | 1.62E-18 | 0.0181 | A | G |
| rs1121985 | 0.0107366 | 0.00145963 | 1.90E-13 | 0.37 | A | C |
| rs112236452 | -0.0478801 | 0.0087775 | 4.90E-08 | 0.00764 | T | C |
| rs112260009 | 0.03384 | 0.00424468 | 1.56E-15 | 0.0302 | A | G |
| rs11226108 | 0.0142732 | 0.00180053 | 2.24E-15 | 0.191 | C | G |
| rs112291945 | -0.0603784 | 0.00406749 | 7.59E-50 | 0.0306 | C | G |
| rs11230285 | 0.00905748 | 0.00152552 | 2.90E-09 | 0.306 | G | A |
| rs11230741 | -0.0132975 | 0.00196818 | 1.42E-11 | 0.182 | A | G |
| rs112346882 | -0.0196339 | 0.00253681 | 9.97E-15 | 0.0825 | A | T |
| rs112354933 | 0.0371226 | 0.00423088 | 1.72E-18 | 0.0319 | C | G |
| rs1123571 | 0.0109643 | 0.00142323 | 1.32E-14 | 0.469 | A | G |
| rs112374398 | -0.0301657 | 0.00494285 | 1.04E-09 | 0.026 | C | G |
| rs112375036 | 0.0137046 | 0.00244848 | 2.18E-08 | 0.0931 | C | T |
| rs11238570 | 0.00957954 | 0.00172714 | 2.91E-08 | 0.205 | T | G |
| rs11244006 | 0.026827 | 0.00347292 | 1.12E-14 | 0.042 | A | G |
| rs11246279 | -0.0082551 | 0.00142593 | 7.07E-09 | 0.477 | A | C |
| rs112468001 | 0.0368703 | 0.00487798 | 4.08E-14 | 0.0252 | A | G |
| rs112481437 | -0.093841 | 0.00497178 | 1.84E-79 | 0.0237 | A | G |
| rs1124835 | 0.0140479 | 0.00155865 | 2.01E-19 | 0.429 | G | A |
| rs11250170 | -0.0085256 | 0.0015606 | 4.68E-08 | 0.396 | T | C |
| rs112507407 | -0.0891121 | 0.00400092 | 6.77E-110 | 0.0334 | G | C |
| rs112542690 | 0.017512 | 0.00244616 | 8.13E-13 | 0.107 | A | C |
| rs112565165 | 0.0276467 | 0.00481429 | 9.32E-09 | 0.0215 | T | C |
| rs112575086 | 0.0139398 | 0.0021344 | 6.53E-11 | 0.12 | T | C |
| rs112585543 | -0.0483326 | 0.00609863 | 2.28E-15 | 0.0132 | T | C |
| rs11259842 | 0.00859046 | 0.00148479 | 7.22E-09 | 0.406 | T | C |
| rs11260029 | -0.0099826 | 0.00155468 | 1.35E-10 | 0.288 | C | T |
| rs11260703 | -0.0114606 | 0.00203443 | 1.77E-08 | 0.137 | C | T |
| rs112625069 | -0.0394813 | 0.00645484 | 9.56E-10 | 0.0155 | T | C |
| rs112660499 | -0.0347932 | 0.00353551 | 7.49E-23 | 0.0412 | A | G |
| rs112672253 | -0.0575641 | 0.00827246 | 3.44E-12 | 0.00782 | T | A |
| rs112705243 | 0.0559534 | 0.00681213 | 2.14E-16 | 0.0116 | A | G |
| rs112771035 | -0.0626229 | 0.00271343 | 7.55E-118 | 0.069 | G | C |
| rs112811239 | 0.0113251 | 0.00169801 | 2.56E-11 | 0.216 | C | T |
| rs112838464 | -0.0135742 | 0.00221831 | 9.41E-10 | 0.11 | A | G |
| rs112868502 | -0.0228765 | 0.00396747 | 8.12E-09 | 0.0332 | A | G |
| rs113003506 | 0.0226011 | 0.00214017 | 4.55E-26 | 0.12 | T | C |
| rs113053770 | 0.010197 | 0.00171704 | 2.87E-09 | 0.213 | T | C |
| rs113105517 | 0.0402117 | 0.00482957 | 8.35E-17 | 0.0218 | G | A |
| rs113342378 | 0.0315751 | 0.00460224 | 6.85E-12 | 0.0244 | A | G |
| rs113345881 | 0.150313 | 0.00241784 | 1.00E-200 | 0.0931 | G | A |
| rs113437942 | -0.022823 | 0.00364054 | 3.63E-10 | 0.0376 | T | C |
| rs1134848 | 0.0116195 | 0.00207238 | 2.06E-08 | 0.133 | T | C |
| rs113580328 | 0.025951 | 0.00302686 | 1.00E-17 | 0.0564 | C | T |
| rs1135908 | -0.0130657 | 0.00200727 | 7.56E-11 | 0.146 | T | G |
| rs113651836 | -0.0104891 | 0.0016241 | 1.06E-10 | 0.284 | A | C |
| rs113654127 | 0.0418186 | 0.00649898 | 1.24E-10 | 0.0126 | A | G |
| rs113760175 | 0.0200291 | 0.00284608 | 1.96E-12 | 0.0652 | A | G |
| rs113770214 | -0.0180782 | 0.00262338 | 5.53E-12 | 0.0905 | T | G |
| rs113784559 | -0.0186524 | 0.00230569 | 5.98E-16 | 0.101 | G | C |
| rs113976905 | -0.0283971 | 0.00319818 | 6.74E-19 | 0.0489 | C | T |
| rs114040256 | 0.0437646 | 0.00791879 | 3.26E-08 | 0.00959 | G | A |
| rs114165349 | -0.0835384 | 0.00462313 | 5.53E-73 | 0.0233 | C | G |
| rs114166723 | 0.0321673 | 0.00505809 | 2.02E-10 | 0.0197 | A | G |
| rs114202167 | -0.0416304 | 0.00708172 | 4.14E-09 | 0.00998 | T | C |
| rs114221654 | 0.0275902 | 0.00295358 | 9.52E-21 | 0.0591 | T | C |
| rs114254196 | 0.0448114 | 0.00818473 | 4.38E-08 | 0.00848 | T | C |
| rs114303452 | -0.0557765 | 0.00699651 | 1.56E-15 | 0.0109 | G | A |
| rs114491880 | -0.0334713 | 0.00533103 | 3.42E-10 | 0.0178 | T | A |
| rs1145210 | 0.00975866 | 0.00150377 | 8.61E-11 | 0.314 | T | C |
| rs114539349 | 0.0248043 | 0.00402118 | 6.90E-10 | 0.0326 | A | G |
| rs114739858 | 0.0339001 | 0.00426239 | 1.82E-15 | 0.0294 | A | G |
| rs114742337 | -0.0202156 | 0.00358526 | 1.72E-08 | 0.0417 | A | G |
| rs114751021 | -0.0442922 | 0.00455688 | 2.48E-22 | 0.0263 | G | A |
| rs114768998 | 0.039198 | 0.00534913 | 2.34E-13 | 0.0184 | A | C |
| rs114796104 | 0.0344081 | 0.00432119 | 1.68E-15 | 0.0316 | T | G |
| rs114960062 | -0.0427377 | 0.00727093 | 4.16E-09 | 0.0115 | A | C |
| rs115114236 | -0.0595224 | 0.00428778 | 8.16E-44 | 0.0284 | C | T |
| rs1151624 | -0.0133161 | 0.00163365 | 3.61E-16 | 0.243 | A | G |
| rs1152002 | 0.0137312 | 0.0014355 | 1.12E-21 | 0.48 | T | C |
| rs115316355 | 0.0297865 | 0.00470024 | 2.34E-10 | 0.023 | C | T |
| rs115383270 | -0.0193347 | 0.00274973 | 2.04E-12 | 0.0734 | A | G |
| rs115394954 | 0.0471471 | 0.00480107 | 9.22E-23 | 0.0219 | C | T |
| rs115430123 | -0.0354494 | 0.00509471 | 3.45E-12 | 0.0206 | C | T |
| rs115478735 | -0.0729888 | 0.00190448 | 1.00E-200 | 0.184 | T | A |
| rs115667974 | -0.0274651 | 0.0043069 | 1.81E-10 | 0.0273 | G | A |
| rs11571788 | 0.0263041 | 0.00367098 | 7.76E-13 | 0.0396 | T | C |
| rs115730757 | -0.0316666 | 0.00497155 | 1.90E-10 | 0.0207 | A | G |
| rs11580642 | -0.0150776 | 0.00140943 | 1.04E-26 | 0.412 | A | T |
| rs115813338 | 0.0310374 | 0.00516645 | 1.88E-09 | 0.0203 | G | A |
| rs11581697 | 0.00955809 | 0.00169567 | 1.73E-08 | 0.212 | T | C |
| rs11584945 | 0.0104188 | 0.00158868 | 5.45E-11 | 0.272 | T | C |
| rs11587316 | -0.0266126 | 0.00432912 | 7.88E-10 | 0.0296 | A | G |
| rs11589982 | -0.0121038 | 0.00143727 | 3.72E-17 | 0.404 | T | C |
| rs11590371 | -0.0097254 | 0.0017192 | 1.54E-08 | 0.207 | C | T |
| rs115931575 | 0.0216168 | 0.00387948 | 2.52E-08 | 0.0357 | C | T |
| rs116001967 | 0.0368941 | 0.00644746 | 1.05E-08 | 0.013 | A | G |
| rs11601507 | -0.0404129 | 0.00317615 | 4.36E-37 | 0.07 | A | C |
| rs116018036 | -0.0208216 | 0.00269386 | 1.08E-14 | 0.0751 | T | C |
| rs116034906 | 0.04899 | 0.00558462 | 1.75E-18 | 0.0169 | T | C |
| rs11605884 | 0.0455774 | 0.00451856 | 6.33E-24 | 0.0237 | C | T |
| rs116066418 | -0.0745214 | 0.00474586 | 1.46E-55 | 0.024 | A | T |
| rs11610264 | -0.0100524 | 0.00159695 | 3.08E-10 | 0.285 | C | T |
| rs11612787 | -0.0403415 | 0.00618525 | 6.93E-11 | 0.0146 | T | C |
| rs116144102 | -0.0415469 | 0.00488236 | 1.75E-17 | 0.021 | A | G |
| rs116183795 | 0.0276478 | 0.00401147 | 5.49E-12 | 0.0306 | A | C |
| rs11621792 | -0.0219774 | 0.00142294 | 8.14E-54 | 0.449 | T | C |
| rs116222550 | 0.0210053 | 0.00378376 | 2.83E-08 | 0.0379 | G | A |
| rs116284390 | 0.0446673 | 0.00516721 | 5.41E-18 | 0.0196 | T | C |
| rs116293108 | -0.0430367 | 0.00464914 | 2.10E-20 | 0.0263 | A | G |
| rs11636087 | -0.0144221 | 0.00154909 | 1.28E-20 | 0.298 | C | T |
| rs116363925 | -0.0303328 | 0.00318075 | 1.48E-21 | 0.0565 | T | G |
| rs116389032 | -0.0283171 | 0.00513061 | 3.40E-08 | 0.0185 | A | T |
| rs116400972 | 0.0132167 | 0.00242072 | 4.77E-08 | 0.0943 | T | C |
| rs11643216 | -0.0104432 | 0.00152107 | 6.62E-12 | 0.293 | T | C |
| rs11648003 | -0.0515795 | 0.00166854 | 1.00E-200 | 0.225 | G | A |
| rs11648622 | -0.0307018 | 0.00157884 | 3.16E-84 | 0.261 | A | G |
| rs116487096 | 0.0287525 | 0.00369307 | 6.94E-15 | 0.039 | A | T |
| rs11650379 | 0.0374921 | 0.00630085 | 2.68E-09 | 0.0135 | G | A |
| rs116517871 | -0.0773142 | 0.00616369 | 4.32E-36 | 0.0134 | A | T |
| rs11655507 | 0.0117003 | 0.00170823 | 7.42E-12 | 0.237 | G | A |
| rs11657987 | -0.0177879 | 0.00140537 | 1.02E-36 | 0.498 | T | G |
| rs116585342 | -0.0502704 | 0.00716576 | 2.29E-12 | 0.0112 | A | T |
| rs116613712 | -0.0316623 | 0.00305687 | 3.86E-25 | 0.0632 | A | G |
| rs11662691 | -0.015538 | 0.00245676 | 2.54E-10 | 0.105 | C | T |
| rs11667653 | 0.010702 | 0.00184627 | 6.77E-09 | 0.19 | T | C |
| rs11667752 | -0.0269614 | 0.00400345 | 1.64E-11 | 0.0348 | A | G |
| rs11668554 | -0.0103758 | 0.00170583 | 1.18E-09 | 0.272 | G | A |
| rs11668861 | -0.0426391 | 0.00140571 | 1.00E-200 | 0.459 | T | G |
| rs11668883 | -0.0132765 | 0.00181626 | 2.68E-13 | 0.189 | G | A |
| rs11668886 | 0.0107433 | 0.00165765 | 9.11E-11 | 0.233 | T | C |
| rs116692022 | -0.0455428 | 0.0064595 | 1.78E-12 | 0.013 | G | A |
| rs116710665 | -0.0528482 | 0.0078185 | 1.39E-11 | 0.0082 | C | T |
| rs11671705 | -0.0136237 | 0.0021236 | 1.40E-10 | 0.161 | T | C |
| rs116734477 | 0.0511426 | 0.00366166 | 2.48E-44 | 0.0377 | T | C |
| rs11673631 | -0.0434571 | 0.00366703 | 2.13E-32 | 0.0399 | C | G |
| rs11675168 | 0.0227666 | 0.00409468 | 2.70E-08 | 0.0291 | A | G |
| rs11676088 | -0.0125762 | 0.00142171 | 9.09E-19 | 0.456 | A | G |
| rs116851371 | -0.120923 | 0.0148447 | 3.77E-16 | 0.00349 | C | A |
| rs11686966 | -0.0308616 | 0.0050067 | 7.09E-10 | 0.0217 | G | C |
| rs116902363 | -0.0660375 | 0.00571998 | 7.82E-31 | 0.0164 | A | G |
| rs116910573 | -0.0279143 | 0.0036055 | 9.78E-15 | 0.0411 | A | G |
| rs1169288 | -0.0358725 | 0.0014912 | 7.20E-128 | 0.322 | C | A |
| rs1169298 | -0.0286066 | 0.00444265 | 1.20E-10 | 0.026 | G | A |
| rs116962591 | 0.0255776 | 0.00440811 | 6.54E-09 | 0.027 | T | C |
| rs116967764 | -0.0574235 | 0.00692573 | 1.12E-16 | 0.0137 | A | G |
| rs116970667 | 0.0295949 | 0.00401996 | 1.81E-13 | 0.0335 | T | C |
| rs116992380 | 0.037105 | 0.00478913 | 9.35E-15 | 0.0232 | C | T |
| rs116996466 | 0.0316459 | 0.00523187 | 1.46E-09 | 0.0195 | G | A |
| rs11699903 | -0.0118316 | 0.00210505 | 1.90E-08 | 0.127 | A | G |
| rs11700304 | -0.0092225 | 0.00145156 | 2.10E-10 | 0.38 | C | T |
| rs117068684 | -0.0243886 | 0.00436646 | 2.33E-08 | 0.0326 | T | C |
| rs117114368 | -0.0273973 | 0.00482636 | 1.37E-08 | 0.0233 | C | G |
| rs117119759 | 0.0287034 | 0.00493703 | 6.10E-09 | 0.0229 | A | G |
| rs11712614 | 0.00904557 | 0.00153412 | 3.72E-09 | 0.303 | A | C |
| rs117139027 | 0.0782787 | 0.00642262 | 3.60E-34 | 0.0131 | A | G |
| rs117142879 | -0.0560386 | 0.00558428 | 1.07E-23 | 0.0178 | T | C |
| rs117147052 | -0.0197158 | 0.00323342 | 1.08E-09 | 0.0528 | C | T |
| rs117210556 | -0.0370836 | 0.00281398 | 1.17E-39 | 0.0654 | C | T |
| rs117339792 | 0.0845834 | 0.00613503 | 3.05E-43 | 0.0155 | A | G |
| rs11736427 | 0.00798323 | 0.00143929 | 2.91E-08 | 0.361 | T | A |
| rs117394684 | 0.0791336 | 0.00837198 | 3.32E-21 | 0.0085 | T | C |
| rs117414940 | 0.0464929 | 0.00473402 | 9.14E-23 | 0.0243 | C | A |
| rs117415600 | -0.0332063 | 0.00587505 | 1.59E-08 | 0.0171 | A | C |
| rs11741997 | 0.038645 | 0.00565145 | 8.03E-12 | 0.0195 | G | A |
| rs11742626 | 0.0233544 | 0.00382482 | 1.02E-09 | 0.0366 | A | G |
| rs117436012 | 0.0198175 | 0.00347449 | 1.17E-08 | 0.0501 | C | T |
| rs11745587 | -0.0162122 | 0.00145221 | 6.13E-29 | 0.355 | A | G |
| rs11751495 | -0.0318865 | 0.00436022 | 2.61E-13 | 0.0357 | G | C |
| rs11759543 | 0.0189606 | 0.00286279 | 3.52E-11 | 0.0661 | A | G |
| rs11759627 | -0.0097159 | 0.00148791 | 6.58E-11 | 0.32 | T | C |
| rs117612826 | -0.060114 | 0.00751743 | 1.28E-15 | 0.0101 | C | T |
| rs11761517 | 0.00818549 | 0.00143473 | 1.16E-08 | 0.461 | T | C |
| rs117623941 | 0.0315372 | 0.00565705 | 2.48E-08 | 0.0173 | T | C |
| rs117655998 | 0.0328219 | 0.00553746 | 3.08E-09 | 0.017 | T | C |
| rs117673551 | 0.0552317 | 0.0046636 | 2.34E-32 | 0.027 | A | G |
| rs117753658 | 0.0572019 | 0.00550396 | 2.67E-25 | 0.0186 | G | A |
| rs117777720 | 0.0147267 | 0.001625 | 1.27E-19 | 0.241 | T | C |
| rs117798643 | 0.0327723 | 0.00377587 | 3.98E-18 | 0.0346 | T | C |
| rs117812504 | -0.0199129 | 0.0035588 | 2.20E-08 | 0.0441 | A | G |
| rs11781960 | 0.0358687 | 0.00499305 | 6.78E-13 | 0.0222 | C | G |
| rs117834294 | 0.149531 | 0.00570255 | 1.50E-151 | 0.0164 | T | C |
| rs11787335 | -0.0231214 | 0.00146651 | 5.31E-56 | 0.355 | T | C |
| rs11791119 | 0.0111509 | 0.00151304 | 1.71E-13 | 0.331 | C | T |
| rs117983270 | 0.0295449 | 0.00372593 | 2.20E-15 | 0.0362 | C | T |
| rs1179992 | 0.012087 | 0.00153147 | 2.96E-15 | 0.314 | A | G |
| rs118014788 | -0.0282179 | 0.00433925 | 7.88E-11 | 0.0293 | T | G |
| rs118024629 | -0.0135705 | 0.0019244 | 1.77E-12 | 0.183 | A | C |
| rs118039278 | -0.113646 | 0.00277251 | 1.00E-200 | 0.0691 | A | G |
| rs118054030 | -0.033001 | 0.00337661 | 1.46E-22 | 0.0457 | A | C |
| rs11807 | 0.00978699 | 0.00173165 | 1.59E-08 | 0.197 | C | T |
| rs118073511 | 0.035128 | 0.00632348 | 2.77E-08 | 0.0177 | A | G |
| rs118097433 | 0.0155064 | 0.00273392 | 1.41E-08 | 0.0715 | T | C |
| rs118104581 | -0.0630255 | 0.00559675 | 2.04E-29 | 0.0191 | A | G |
| rs118115977 | -0.0393844 | 0.00619626 | 2.07E-10 | 0.0143 | T | C |
| rs118127759 | 0.0415551 | 0.00604371 | 6.17E-12 | 0.0139 | T | C |
| rs11824446 | -0.0219943 | 0.00370092 | 2.80E-09 | 0.037 | C | G |
| rs11837065 | 0.0111241 | 0.00147305 | 4.29E-14 | 0.366 | T | C |
| rs11845606 | 0.0239472 | 0.00296893 | 7.27E-16 | 0.0602 | G | C |
| rs11846704 | 0.0137613 | 0.00156628 | 1.55E-18 | 0.267 | T | C |
| rs1186380 | 0.0156254 | 0.00166213 | 5.41E-21 | 0.242 | T | C |
| rs11870307 | -0.0099525 | 0.00170088 | 4.88E-09 | 0.22 | A | G |
| rs11881404 | -0.0089373 | 0.00144268 | 5.83E-10 | 0.371 | A | G |
| rs11881955 | -0.0098632 | 0.00155716 | 2.39E-10 | 0.391 | G | A |
| rs11887443 | 0.0124671 | 0.00175824 | 1.33E-12 | 0.263 | T | G |
| rs1188827 | -0.0109059 | 0.00179047 | 1.12E-09 | 0.189 | C | G |
| rs11897825 | -0.0179958 | 0.00153415 | 8.93E-32 | 0.433 | G | A |
| rs11899537 | 0.00943472 | 0.00148664 | 2.21E-10 | 0.34 | T | C |
| rs11911615 | 0.00812262 | 0.00148967 | 4.96E-08 | 0.324 | G | T |
| rs11957820 | -0.0216057 | 0.00282986 | 2.26E-14 | 0.0624 | T | C |
| rs1196760 | -0.0202432 | 0.00245351 | 1.57E-16 | 0.0922 | G | C |
| rs11977408 | -0.0176553 | 0.00323356 | 4.76E-08 | 0.0559 | T | C |
| rs11993666 | -0.0287777 | 0.00274723 | 1.12E-25 | 0.0705 | A | T |
| rs11993679 | 0.0122008 | 0.00171534 | 1.14E-12 | 0.221 | C | T |
| rs12016920 | 0.0171653 | 0.00180179 | 1.62E-21 | 0.193 | C | T |
| rs12022410 | 0.00774701 | 0.00141111 | 4.02E-08 | 0.45 | A | G |
| rs12024044 | 0.0169062 | 0.00180826 | 8.81E-21 | 0.181 | A | G |
| rs1203110 | -0.0125024 | 0.0022933 | 4.99E-08 | 0.104 | G | C |
| rs12031153 | -0.0331403 | 0.00346303 | 1.07E-21 | 0.0531 | A | G |
| rs12043403 | 0.024476 | 0.00246425 | 3.01E-23 | 0.0975 | C | T |
| rs12045893 | -0.0108397 | 0.00160036 | 1.26E-11 | 0.248 | T | C |
| rs12055389 | 0.0277593 | 0.003005 | 2.52E-20 | 0.0574 | T | C |
| rs12081530 | -0.0424727 | 0.00140367 | 1.00E-200 | 0.448 | A | G |
| rs12094989 | 0.0129269 | 0.00169858 | 2.73E-14 | 0.215 | T | C |
| rs12104440 | 0.00960284 | 0.00141324 | 1.08E-11 | 0.404 | T | C |
| rs12106385 | 0.0364153 | 0.00551245 | 3.95E-11 | 0.0185 | A | T |
| rs12114418 | -0.0215482 | 0.00165601 | 1.04E-38 | 0.229 | G | A |
| rs12117480 | 0.0248106 | 0.00373416 | 3.05E-11 | 0.0371 | A | C |
| rs12117661 | 0.0659791 | 0.00164207 | 1.00E-200 | 0.242 | G | C |
| rs12119154 | -0.0298826 | 0.00287815 | 2.98E-25 | 0.0644 | A | G |
| rs12121214 | -0.0133813 | 0.00241999 | 3.21E-08 | 0.0901 | C | T |
| rs12123143 | -0.0131509 | 0.00225602 | 5.57E-09 | 0.229 | C | T |
| rs12136217 | -0.0348439 | 0.00293636 | 1.77E-32 | 0.0607 | A | G |
| rs12141871 | -0.0089702 | 0.00143045 | 3.59E-10 | 0.487 | A | G |
| rs12143028 | -0.0289795 | 0.0022628 | 1.50E-37 | 0.128 | C | G |
| rs12150495 | 0.0161159 | 0.00264098 | 1.05E-09 | 0.0767 | A | G |
| rs12150984 | -0.0137684 | 0.00168015 | 2.51E-16 | 0.287 | A | G |
| rs12208357 | -0.0644647 | 0.00269768 | 3.34E-126 | 0.0696 | T | C |
| rs12214384 | -0.0132045 | 0.00208689 | 2.49E-10 | 0.126 | T | C |
| rs12214416 | 0.0269762 | 0.00336552 | 1.10E-15 | 0.0477 | A | T |
| rs12221682 | -0.0287155 | 0.00248422 | 6.64E-31 | 0.0856 | G | C |
| rs12243832 | -0.0249228 | 0.00238143 | 1.24E-25 | 0.0933 | T | G |
| rs12289703 | 0.0212652 | 0.00364148 | 5.23E-09 | 0.0378 | A | G |
| rs12295353 | 0.00784086 | 0.00139424 | 1.87E-08 | 0.48 | C | T |
| rs1229984 | 0.0403261 | 0.00434858 | 1.80E-20 | 0.0283 | T | C |
| rs12306780 | -0.0115655 | 0.00146541 | 2.97E-15 | 0.342 | T | A |
| rs12342395 | -0.0091902 | 0.00147027 | 4.09E-10 | 0.365 | T | C |
| rs1235381 | -0.0147709 | 0.00140163 | 5.75E-26 | 0.483 | C | T |
| rs12355391 | -0.0107881 | 0.00144595 | 8.59E-14 | 0.428 | C | A |
| rs12360261 | 0.0162165 | 0.0026429 | 8.47E-10 | 0.0768 | G | A |
| rs12369156 | -0.0347313 | 0.004883 | 1.14E-12 | 0.0215 | A | G |
| rs123698 | 0.00888028 | 0.00161818 | 4.07E-08 | 0.384 | G | C |
| rs12409233 | 0.0225537 | 0.00244129 | 2.50E-20 | 0.0882 | C | G |
| rs12418845 | 0.0175061 | 0.00276334 | 2.37E-10 | 0.071 | A | G |
| rs12420014 | 0.019783 | 0.0035397 | 2.29E-08 | 0.0417 | C | T |
| rs12423190 | -0.0256148 | 0.00253916 | 6.25E-24 | 0.0835 | C | T |
| rs12425848 | 0.00904063 | 0.00153482 | 3.85E-09 | 0.306 | A | G |
| rs12443646 | 0.0081252 | 0.00144703 | 1.96E-08 | 0.367 | C | T |
| rs12445804 | -0.0325513 | 0.00267884 | 5.65E-34 | 0.0763 | A | G |
| rs12453354 | 0.020968 | 0.00307497 | 9.17E-12 | 0.0566 | A | G |
| rs12454507 | -0.009929 | 0.00159069 | 4.32E-10 | 0.284 | G | A |
| rs12459686 | -0.0195215 | 0.00147654 | 6.63E-40 | 0.42 | A | C |
| rs12460033 | -0.0124982 | 0.00138905 | 2.31E-19 | 0.475 | A | G |
| rs12460535 | 0.010905 | 0.00149473 | 2.97E-13 | 0.332 | A | G |
| rs12461144 | -0.0201276 | 0.00212575 | 2.84E-21 | 0.135 | T | C |
| rs12461923 | -0.0118075 | 0.00194701 | 1.32E-09 | 0.17 | T | C |
| rs12462616 | 0.0152731 | 0.0017319 | 1.16E-18 | 0.206 | C | A |
| rs1247295 | 0.0140022 | 0.00140461 | 2.09E-23 | 0.453 | G | T |
| rs12474279 | 0.00784932 | 0.00140551 | 2.34E-08 | 0.431 | A | G |
| rs1247572 | 0.00983281 | 0.00178791 | 3.81E-08 | 0.186 | G | A |
| rs12480826 | -0.0111828 | 0.00192052 | 5.79E-09 | 0.154 | A | C |
| rs12500824 | -0.0092536 | 0.00145454 | 1.99E-10 | 0.346 | A | G |
| rs1250259 | 0.0176512 | 0.00159409 | 1.70E-28 | 0.264 | T | A |
| rs12526480 | 0.0084351 | 0.00147877 | 1.17E-08 | 0.332 | G | T |
| rs12533280 | -0.0180348 | 0.00175998 | 1.22E-24 | 0.195 | T | C |
| rs12551960 | -0.0349877 | 0.00264871 | 7.75E-40 | 0.0786 | T | C |
| rs1257219 | 0.0107392 | 0.00165026 | 7.64E-11 | 0.23 | A | G |
| rs12597024 | 0.0122149 | 0.00150763 | 5.40E-16 | 0.338 | C | T |
| rs1260326 | -0.0306622 | 0.00141948 | 1.76E-103 | 0.39 | T | C |
| rs12603290 | -0.0267572 | 0.00143656 | 1.98E-77 | 0.495 | T | C |
| rs12608665 | 0.0131702 | 0.00184662 | 9.89E-13 | 0.194 | T | C |
| rs12609589 | -0.0445432 | 0.00200132 | 9.67E-110 | 0.157 | T | C |
| rs12610374 | 0.0556813 | 0.00160974 | 1.00E-200 | 0.249 | T | C |
| rs12614487 | 0.026633 | 0.0026376 | 5.67E-24 | 0.074 | T | C |
| rs12646327 | -0.0089758 | 0.00139516 | 1.25E-10 | 0.483 | T | G |
| rs12646808 | -0.0095052 | 0.00151931 | 3.94E-10 | 0.335 | C | T |
| rs12657266 | 0.0353193 | 0.00143731 | 2.44E-133 | 0.365 | C | T |
| rs12662589 | -0.015299 | 0.00158398 | 4.52E-22 | 0.259 | C | G |
| rs12670950 | -0.0210788 | 0.00330422 | 1.78E-10 | 0.0457 | A | C |
| rs12681194 | 0.0082448 | 0.00150652 | 4.43E-08 | 0.307 | A | C |
| rs12693968 | -0.0212882 | 0.00159951 | 2.05E-40 | 0.258 | A | G |
| rs12693975 | 0.022401 | 0.0018313 | 2.09E-34 | 0.18 | G | A |
| rs12708983 | 0.0245352 | 0.00385557 | 1.97E-10 | 0.0362 | C | T |
| rs12712955 | -0.0114588 | 0.00139377 | 2.01E-16 | 0.497 | A | G |
| rs12713758 | -0.0099741 | 0.00178768 | 2.41E-08 | 0.185 | T | C |
| rs12720358 | 0.0543406 | 0.00495544 | 5.58E-28 | 0.0201 | T | C |
| rs12720820 | 0.0779623 | 0.00249535 | 1.00E-200 | 0.0835 | C | T |
| rs12720917 | 0.0117445 | 0.00206293 | 1.25E-08 | 0.147 | C | T |
| rs12721041 | -0.0440182 | 0.00573365 | 1.63E-14 | 0.0149 | T | C |
| rs12725027 | -0.0116518 | 0.00169807 | 6.80E-12 | 0.22 | A | G |
| rs12732125 | 0.173907 | 0.00524615 | 1.00E-200 | 0.0188 | T | C |
| rs12736858 | -0.014049 | 0.00234794 | 2.18E-09 | 0.115 | T | C |
| rs12739979 | 0.0270434 | 0.00181243 | 2.40E-50 | 0.238 | T | C |
| rs12750160 | 0.305012 | 0.00786547 | 1.00E-200 | 0.00884 | T | C |
| rs12758665 | -0.0135085 | 0.00172114 | 4.21E-15 | 0.21 | T | G |
| rs1275923 | 0.00980839 | 0.0014348 | 8.14E-12 | 0.4 | C | T |
| rs12811045 | -0.0101332 | 0.00174496 | 6.36E-09 | 0.211 | G | A |
| rs12897637 | -0.015518 | 0.00190214 | 3.40E-16 | 0.16 | C | T |
| rs12917376 | 0.0125937 | 0.00142309 | 8.79E-19 | 0.423 | C | T |
| rs1292061 | 0.00994871 | 0.0013993 | 1.16E-12 | 0.451 | A | G |
| rs12930656 | 0.0333314 | 0.00537184 | 5.48E-10 | 0.0191 | A | G |
| rs12943633 | 0.0177676 | 0.00255433 | 3.50E-12 | 0.0812 | T | C |
| rs12944954 | -0.0275996 | 0.00472073 | 5.02E-09 | 0.0219 | G | A |
| rs12945088 | 0.0181711 | 0.00160909 | 1.42E-29 | 0.307 | G | A |
| rs12950377 | -0.0127952 | 0.00195346 | 5.75E-11 | 0.151 | C | T |
| rs12963082 | -0.0105962 | 0.00154604 | 7.19E-12 | 0.279 | C | T |
| rs12968116 | -0.0142034 | 0.00209432 | 1.19E-11 | 0.126 | T | C |
| rs12972156 | -0.160779 | 0.00199196 | 1.00E-200 | 0.146 | G | C |
| rs12972282 | 0.0195169 | 0.00166315 | 8.44E-32 | 0.234 | C | G |
| rs12975458 | -0.0213636 | 0.00305638 | 2.75E-12 | 0.0573 | A | G |
| rs12976203 | -0.0583046 | 0.00609858 | 1.17E-21 | 0.0201 | A | T |
| rs12982294 | -0.0114915 | 0.00185797 | 6.21E-10 | 0.204 | T | C |
| rs12983990 | 0.0142707 | 0.00186528 | 2.00E-14 | 0.167 | G | A |
| rs12984134 | -0.014361 | 0.00263125 | 4.82E-08 | 0.078 | G | A |
| rs12984266 | 0.0186703 | 0.0015546 | 3.16E-33 | 0.278 | A | G |
| rs12987350 | 0.0128332 | 0.00157608 | 3.87E-16 | 0.268 | G | A |
| rs13005230 | 0.0179656 | 0.00263828 | 9.79E-12 | 0.0999 | A | C |
| rs13008424 | -0.0086951 | 0.00155788 | 2.39E-08 | 0.414 | G | A |
| rs13013895 | 0.0116532 | 0.00209058 | 2.49E-08 | 0.158 | G | C |
| rs13014455 | -0.0099082 | 0.00165317 | 2.05E-09 | 0.239 | C | T |
| rs13015576 | -0.0119816 | 0.00161647 | 1.24E-13 | 0.245 | C | T |
| rs13023530 | -0.0190712 | 0.0025721 | 1.22E-13 | 0.0781 | A | G |
| rs13037749 | 0.0151349 | 0.00271412 | 2.46E-08 | 0.0722 | G | A |
| rs13076933 | 0.0245071 | 0.00159894 | 5.04E-53 | 0.257 | G | T |
| rs13107325 | 0.0251333 | 0.00278802 | 1.97E-19 | 0.0643 | T | C |
| rs13108218 | -0.0182714 | 0.00146485 | 1.05E-35 | 0.385 | A | G |
| rs13168671 | 0.0124253 | 0.00168539 | 1.68E-13 | 0.214 | G | A |
| rs13173241 | -0.0156087 | 0.00173918 | 2.84E-19 | 0.195 | A | G |
| rs13179861 | 0.0122792 | 0.00182108 | 1.55E-11 | 0.178 | A | G |
| rs13190798 | -0.0121895 | 0.00176714 | 5.28E-12 | 0.194 | A | G |
| rs13202307 | 0.0173643 | 0.00310905 | 2.34E-08 | 0.0544 | A | G |
| rs13207901 | 0.0322721 | 0.00207364 | 1.30E-54 | 0.13 | T | A |
| rs13213129 | 0.0283925 | 0.00346314 | 2.43E-16 | 0.0443 | T | G |
| rs13220896 | -0.0247808 | 0.00372167 | 2.77E-11 | 0.0385 | A | G |
| rs13232198 | -0.0090716 | 0.00151115 | 1.94E-09 | 0.44 | G | A |
| rs13233422 | 0.0102623 | 0.00168868 | 1.22E-09 | 0.221 | T | C |
| rs13254216 | -0.0212673 | 0.00294511 | 5.15E-13 | 0.0586 | C | T |
| rs13256216 | -0.0125804 | 0.00161505 | 6.73E-15 | 0.25 | T | C |
| rs1326122 | 0.0262708 | 0.0047048 | 2.35E-08 | 0.0232 | A | C |
| rs132646 | -0.0133892 | 0.00186524 | 7.06E-13 | 0.166 | T | C |
| rs13268 | 0.0379802 | 0.00452552 | 4.76E-17 | 0.0242 | G | A |
| rs13289095 | 0.0288711 | 0.002062 | 1.53E-44 | 0.137 | T | G |
| rs1329125 | -0.0220302 | 0.00149651 | 4.72E-49 | 0.321 | T | C |
| rs13301187 | -0.0166499 | 0.00187841 | 7.73E-19 | 0.172 | A | C |
| rs13301660 | 0.0156411 | 0.00156988 | 2.21E-23 | 0.27 | T | C |
| rs1333744 | 0.0323173 | 0.00483208 | 2.26E-11 | 0.0235 | C | T |
| rs13344768 | 0.0222966 | 0.00285373 | 5.58E-15 | 0.0623 | A | G |
| rs13379043 | 0.0165377 | 0.00156818 | 5.31E-26 | 0.271 | C | T |
| rs13397898 | 0.0698146 | 0.00656719 | 2.14E-26 | 0.0113 | A | G |
| rs13403394 | 0.0262537 | 0.00397503 | 3.98E-11 | 0.0313 | T | C |
| rs13412 | 0.00899343 | 0.00146098 | 7.47E-10 | 0.356 | C | T |
| rs13438525 | -0.0151205 | 0.00139827 | 2.96E-27 | 0.492 | C | T |
| rs13621 | -0.010972 | 0.00139783 | 4.18E-15 | 0.462 | C | T |
| rs1375131 | -0.0195868 | 0.00172675 | 8.02E-30 | 0.313 | C | T |
| rs137872213 | -0.041304 | 0.00516451 | 1.27E-15 | 0.0219 | G | C |
| rs137928795 | -0.118711 | 0.0126614 | 6.86E-21 | 0.00378 | T | C |
| rs137932185 | -0.0249476 | 0.00369268 | 1.42E-11 | 0.0375 | T | C |
| rs137982819 | -0.0824129 | 0.00528791 | 9.18E-55 | 0.0204 | A | G |
| rs138192339 | -0.0382097 | 0.00607998 | 3.29E-10 | 0.0155 | A | C |
| rs138204164 | 0.0145038 | 0.00206177 | 2.00E-12 | 0.132 | G | C |
| rs138352 | -0.0137988 | 0.00147717 | 9.51E-21 | 0.346 | T | G |
| rs138361368 | 0.0942756 | 0.00518715 | 8.17E-74 | 0.0184 | A | C |
| rs138377780 | -0.038831 | 0.0055385 | 2.36E-12 | 0.0185 | T | C |
| rs138549028 | -0.0365891 | 0.00609388 | 1.92E-09 | 0.0149 | T | C |
| rs138557630 | 0.0571654 | 0.00852892 | 2.05E-11 | 0.00764 | T | C |
| rs138607350 | -0.150302 | 0.00778907 | 5.74E-83 | 0.00856 | G | T |
| rs138683771 | -0.0542863 | 0.00594597 | 6.85E-20 | 0.015 | C | A |
| rs138762595 | 0.0378868 | 0.0058722 | 1.10E-10 | 0.0181 | A | G |
| rs139020749 | -0.0327911 | 0.00467616 | 2.34E-12 | 0.0225 | A | G |
| rs139029940 | -0.0348276 | 0.00199021 | 1.45E-68 | 0.152 | A | C |
| rs139066750 | -0.0281099 | 0.00491514 | 1.07E-08 | 0.0239 | A | G |
| rs139134971 | 0.0423345 | 0.00674923 | 3.55E-10 | 0.0115 | T | C |
| rs139140823 | -0.0282566 | 0.00478992 | 3.65E-09 | 0.0249 | T | C |
| rs139198665 | -0.0613961 | 0.00479989 | 1.84E-37 | 0.0257 | T | C |
| rs139262716 | 0.0386898 | 0.00533428 | 4.07E-13 | 0.019 | A | G |
| rs139273281 | 0.0448308 | 0.00561628 | 1.44E-15 | 0.0169 | A | G |
| rs139294635 | 0.0619098 | 0.0102102 | 1.33E-09 | 0.0054 | C | T |
| rs139305029 | -0.030229 | 0.00394481 | 1.82E-14 | 0.0364 | T | C |
| rs139311191 | -0.0408135 | 0.0037248 | 6.13E-28 | 0.0392 | A | G |
| rs139338974 | -0.0307707 | 0.00554605 | 2.89E-08 | 0.0185 | T | G |
| rs139425456 | -0.10207 | 0.00718558 | 8.55E-46 | 0.0121 | A | T |
| rs139524394 | -0.0639692 | 0.00732382 | 2.45E-18 | 0.0101 | T | C |
| rs139529904 | 0.0581918 | 0.00673493 | 5.61E-18 | 0.0114 | C | A |
| rs139580521 | -0.0589452 | 0.0101711 | 6.82E-09 | 0.00572 | C | T |
| rs139760949 | -0.106113 | 0.0079141 | 5.42E-41 | 0.00789 | A | C |
| rs139768754 | 0.0459783 | 0.00684715 | 1.88E-11 | 0.0127 | T | C |
| rs139907312 | -0.0549247 | 0.00777923 | 1.66E-12 | 0.00953 | A | G |
| rs139943078 | 0.0304358 | 0.00511529 | 2.68E-09 | 0.0253 | T | G |
| rs139953956 | 0.0407628 | 0.0062901 | 9.14E-11 | 0.0153 | G | A |
| rs139956529 | -0.0339617 | 0.00594502 | 1.11E-08 | 0.016 | T | C |
| rs139957766 | 0.0448267 | 0.00419296 | 1.12E-26 | 0.0292 | A | G |
| rs139995984 | 0.245706 | 0.00745234 | 1.00E-200 | 0.0116 | C | G |
| rs14000 | -0.0336003 | 0.00223987 | 7.23E-51 | 0.109 | C | T |
| rs140381712 | -0.0355686 | 0.00445377 | 1.39E-15 | 0.0267 | C | G |
| rs140490424 | -0.0204147 | 0.00330224 | 6.33E-10 | 0.0513 | T | C |
| rs1406964 | 0.010135 | 0.00156746 | 1.01E-10 | 0.28 | C | T |
| rs140787215 | 0.0278627 | 0.00474117 | 4.18E-09 | 0.0242 | T | G |
| rs1409385 | 0.0117957 | 0.0015392 | 1.81E-14 | 0.296 | A | G |
| rs140959139 | 0.0873551 | 0.00759539 | 1.30E-30 | 0.00973 | A | G |
| rs140965804 | -0.0613751 | 0.0111046 | 3.26E-08 | 0.00453 | A | G |
| rs141169907 | -0.0398246 | 0.00571416 | 3.18E-12 | 0.0176 | T | C |
| rs141321280 | -0.0370018 | 0.00626588 | 3.52E-09 | 0.0135 | A | G |
| rs141328036 | 0.0826089 | 0.00645534 | 1.70E-37 | 0.0122 | G | A |
| rs141414463 | -0.0476194 | 0.00453476 | 8.55E-26 | 0.0241 | T | C |
| rs141469619 | -0.0725318 | 0.00796181 | 8.24E-20 | 0.0092 | G | A |
| rs141481562 | -0.0739245 | 0.00961434 | 1.48E-14 | 0.00656 | G | A |
| rs141521383 | -0.0374433 | 0.00500837 | 7.65E-14 | 0.0225 | C | G |
| rs141530948 | 0.0558406 | 0.00941168 | 2.97E-09 | 0.00619 | A | G |
| rs141597891 | 0.101866 | 0.00809552 | 2.62E-36 | 0.00792 | G | A |
| rs141645791 | 0.10372 | 0.006754 | 3.19E-53 | 0.0119 | T | C |
| rs141739979 | 0.0541638 | 0.00751643 | 5.76E-13 | 0.01 | T | G |
| rs141778417 | -0.0545484 | 0.00718869 | 3.25E-14 | 0.00999 | T | C |
| rs141783576 | -0.0240255 | 0.0030723 | 5.28E-15 | 0.069 | C | G |
| rs141804284 | -0.051759 | 0.0058894 | 1.52E-18 | 0.016 | C | A |
| rs141832109 | -0.0394004 | 0.00553159 | 1.06E-12 | 0.0184 | C | A |
| rs141915547 | -0.0362767 | 0.00575592 | 2.93E-10 | 0.0173 | A | G |
| rs141964576 | -0.0722444 | 0.00913182 | 2.55E-15 | 0.00706 | G | C |
| rs141970801 | 0.127885 | 0.00356922 | 1.00E-200 | 0.0401 | A | G |
| rs141981219 | -0.0413771 | 0.00691828 | 2.22E-09 | 0.0116 | T | A |
| rs141989097 | -0.0352073 | 0.00243291 | 1.84E-47 | 0.0894 | T | C |
| rs142138315 | 0.0553078 | 0.00931336 | 2.88E-09 | 0.00691 | T | C |
| rs142288668 | 0.0318368 | 0.00500135 | 1.94E-10 | 0.0213 | A | G |
| rs142314783 | -0.0238183 | 0.00401098 | 2.88E-09 | 0.0349 | G | A |
| rs142380271 | -0.109073 | 0.00754758 | 2.46E-47 | 0.0104 | G | A |
| rs142385484 | 0.0186184 | 0.0020093 | 1.93E-20 | 0.153 | T | C |
| rs142620594 | -0.021922 | 0.00299512 | 2.49E-13 | 0.0567 | G | C |
| rs142710807 | 0.0344739 | 0.00531629 | 8.90E-11 | 0.0189 | T | C |
| rs142787485 | 0.0285729 | 0.00397962 | 6.98E-13 | 0.036 | G | A |
| rs142791622 | 0.0596233 | 0.00476907 | 7.27E-36 | 0.0224 | A | G |
| rs142972972 | -0.0367538 | 0.0058558 | 3.46E-10 | 0.0141 | C | A |
| rs143098225 | -0.0188165 | 0.00314472 | 2.18E-09 | 0.0574 | C | T |
| rs143116067 | 0.0200868 | 0.00293994 | 8.35E-12 | 0.0675 | A | G |
| rs143164503 | 0.0301661 | 0.00515866 | 4.99E-09 | 0.0199 | A | C |
| rs143303864 | 0.0267664 | 0.00490819 | 4.94E-08 | 0.0223 | A | G |
| rs143307443 | 0.0208289 | 0.00367299 | 1.42E-08 | 0.0398 | G | A |
| rs143368951 | 0.0750574 | 0.00732292 | 1.19E-24 | 0.0105 | G | A |
| rs143373095 | 0.0590168 | 0.00710871 | 1.02E-16 | 0.0105 | G | A |
| rs143392466 | 0.0513566 | 0.00932031 | 3.58E-08 | 0.0056 | C | G |
| rs1434282 | 0.0109297 | 0.00157384 | 3.80E-12 | 0.277 | C | T |
| rs143445653 | -0.0157411 | 0.0028679 | 4.05E-08 | 0.0635 | C | T |
| rs143587805 | -0.0630972 | 0.00668994 | 4.04E-21 | 0.0115 | T | A |
| rs143588012 | 0.0411444 | 0.00447586 | 3.84E-20 | 0.0258 | C | G |
| rs143706998 | 0.112884 | 0.00808534 | 2.67E-44 | 0.00809 | C | A |
| rs143710098 | 0.060699 | 0.00777943 | 6.07E-15 | 0.00912 | G | A |
| rs144006934 | 0.0291864 | 0.00517153 | 1.66E-08 | 0.0202 | A | G |
| rs144083983 | 0.0418179 | 0.00299336 | 2.37E-44 | 0.0702 | T | C |
| rs144177163 | 0.0382739 | 0.00442516 | 5.19E-18 | 0.0265 | A | C |
| rs144311893 | 0.49968 | 0.00510622 | 1.00E-200 | 0.0199 | T | C |
| rs144439590 | 0.130753 | 0.00437337 | 2.12E-196 | 0.0272 | T | C |
| rs144456114 | 0.0237394 | 0.00373668 | 2.11E-10 | 0.0378 | C | T |
| rs144750362 | 0.0608648 | 0.0101153 | 1.78E-09 | 0.00619 | C | T |
| rs144778381 | -0.0329224 | 0.00461466 | 9.73E-13 | 0.0263 | G | C |
| rs144833821 | 0.0498411 | 0.00760725 | 5.69E-11 | 0.00941 | C | T |
| rs145125135 | -0.0099896 | 0.00178942 | 2.37E-08 | 0.197 | T | C |
| rs145197288 | 0.02664 | 0.00390754 | 9.26E-12 | 0.038 | A | G |
| rs145208519 | -0.0600075 | 0.00456212 | 1.63E-39 | 0.0264 | T | C |
| rs145288624 | -0.0280735 | 0.00300125 | 8.44E-21 | 0.0655 | T | C |
| rs145400326 | 0.0259983 | 0.00359771 | 4.96E-13 | 0.0424 | C | T |
| rs145434473 | -0.0493288 | 0.00820433 | 1.83E-09 | 0.0084 | A | G |
| rs145467679 | -0.0608044 | 0.00783228 | 8.27E-15 | 0.00843 | C | T |
| rs145494710 | -0.0185269 | 0.0027512 | 1.65E-11 | 0.0709 | A | T |
| rs145639166 | -0.0344787 | 0.0042745 | 7.26E-16 | 0.0383 | G | A |
| rs145655010 | 0.0395954 | 0.00630171 | 3.32E-10 | 0.0138 | C | G |
| rs145713446 | -0.0970848 | 0.0095988 | 4.78E-24 | 0.00634 | A | T |
| rs145734283 | 0.0217532 | 0.00396585 | 4.13E-08 | 0.0355 | A | G |
| rs145770462 | 0.0354 | 0.00485713 | 3.14E-13 | 0.0245 | T | C |
| rs146006317 | 0.0301855 | 0.00422136 | 8.64E-13 | 0.0298 | C | T |
| rs146063554 | 0.0375328 | 0.0066363 | 1.55E-08 | 0.0115 | G | C |
| rs146229119 | -0.0631149 | 0.00995433 | 2.29E-10 | 0.00551 | T | C |
| rs146335137 | -0.0617877 | 0.00773829 | 1.41E-15 | 0.00937 | T | C |
| rs146383065 | 0.0355073 | 0.00468746 | 3.59E-14 | 0.0239 | A | G |
| rs146480899 | 0.0364734 | 0.005179 | 1.89E-12 | 0.0198 | G | A |
| rs146496844 | -0.0637803 | 0.00548416 | 2.90E-31 | 0.0173 | A | G |
| rs146521611 | -0.035423 | 0.00585302 | 1.43E-09 | 0.0158 | A | C |
| rs146534110 | -0.0760646 | 0.00664093 | 2.25E-30 | 0.0115 | T | G |
| rs146534767 | 0.0444546 | 0.00392261 | 9.02E-30 | 0.0414 | G | A |
| rs146568567 | 0.12254 | 0.0103803 | 3.68E-32 | 0.00512 | A | G |
| rs146576912 | 0.155528 | 0.00348456 | 1.00E-200 | 0.051 | T | C |
| rs146674238 | -0.047162 | 0.00654042 | 5.56E-13 | 0.0143 | T | A |
| rs146720479 | 0.0269016 | 0.00289471 | 1.49E-20 | 0.0619 | G | A |
| rs146765950 | 0.0635434 | 0.0049101 | 2.63E-38 | 0.0208 | G | A |
| rs146828936 | -0.0421689 | 0.00674568 | 4.07E-10 | 0.0148 | A | C |
| rs146837680 | 0.0334103 | 0.00512798 | 7.25E-11 | 0.0179 | T | C |
| rs146898772 | -0.0261499 | 0.00436678 | 2.12E-09 | 0.0262 | T | C |
| rs146934308 | 0.0564202 | 0.00872531 | 1.00E-10 | 0.00754 | T | C |
| rs147188206 | -0.0907519 | 0.00651312 | 3.95E-44 | 0.0128 | C | T |
| rs147223298 | 0.0369144 | 0.00543446 | 1.10E-11 | 0.0194 | T | C |
| rs147223423 | -0.0999099 | 0.0070951 | 4.93E-45 | 0.0101 | T | A |
| rs147275653 | 0.0454529 | 0.00646953 | 2.13E-12 | 0.0133 | C | T |
| rs1474582 | 0.00895107 | 0.00142856 | 3.71E-10 | 0.418 | A | G |
| rs147540853 | 0.0911257 | 0.00429151 | 4.64E-100 | 0.0262 | A | G |
| rs147591082 | 0.0308656 | 0.00512034 | 1.66E-09 | 0.0199 | G | A |
| rs147654565 | 0.02343 | 0.00392707 | 2.43E-09 | 0.033 | A | G |
| rs147667955 | 0.0933434 | 0.00730847 | 2.35E-37 | 0.00898 | T | C |
| rs147776329 | -0.0462497 | 0.00708761 | 6.78E-11 | 0.0114 | A | G |
| rs147791730 | 0.0290811 | 0.00417575 | 3.30E-12 | 0.028 | A | G |
| rs147804455 | 0.0353923 | 0.00455659 | 8.02E-15 | 0.0242 | C | A |
| rs148019457 | -0.0382538 | 0.00619378 | 6.57E-10 | 0.0143 | T | C |
| rs148203833 | 0.0379478 | 0.00513292 | 1.44E-13 | 0.0197 | T | A |
| rs148206901 | 0.0282403 | 0.00484572 | 5.61E-09 | 0.0227 | G | C |
| rs148356565 | 0.0739852 | 0.00335232 | 6.16E-108 | 0.0584 | C | T |
| rs148415561 | -0.018457 | 0.00297777 | 5.71E-10 | 0.0612 | T | C |
| rs148427314 | 0.0293948 | 0.00423837 | 4.05E-12 | 0.0295 | C | T |
| rs148430250 | 0.028172 | 0.00352681 | 1.37E-15 | 0.0446 | A | G |
| rs148432373 | -0.0447291 | 0.00759725 | 3.92E-09 | 0.00977 | T | C |
| rs148503795 | 0.0423267 | 0.0070977 | 2.47E-09 | 0.0105 | G | C |
| rs148539727 | 0.0284255 | 0.0048103 | 3.44E-09 | 0.0239 | A | T |
| rs148601586 | -0.161672 | 0.0066786 | 1.86E-129 | 0.0116 | G | C |
| rs148724334 | -0.0427816 | 0.00584071 | 2.39E-13 | 0.0171 | C | G |
| rs148742777 | -0.0622189 | 0.00743221 | 5.69E-17 | 0.0107 | C | T |
| rs148948901 | -0.0329529 | 0.00557379 | 3.38E-09 | 0.0168 | G | A |
| rs149036528 | -0.0519962 | 0.00712986 | 3.04E-13 | 0.0107 | C | T |
| rs149109487 | 0.0414005 | 0.00506306 | 2.91E-16 | 0.0198 | C | G |
| rs149314105 | 0.0338636 | 0.00500413 | 1.31E-11 | 0.0275 | T | C |
| rs149384995 | 0.0288689 | 0.00510949 | 1.60E-08 | 0.0232 | T | C |
| rs149397995 | -0.0762744 | 0.00635066 | 3.13E-33 | 0.0136 | G | T |
| rs149420193 | 0.036456 | 0.00550953 | 3.67E-11 | 0.0183 | T | C |
| rs149516707 | 0.0460811 | 0.00518182 | 5.96E-19 | 0.0201 | G | C |
| rs1495741 | -0.021999 | 0.0016595 | 4.14E-40 | 0.221 | G | A |
| rs149596464 | -0.0190973 | 0.00278072 | 6.52E-12 | 0.0896 | A | G |
| rs1497406 | 0.0136287 | 0.00141025 | 4.29E-22 | 0.416 | A | G |
| rs149801887 | 0.0516874 | 0.00593511 | 3.07E-18 | 0.0166 | T | A |
| rs149807892 | 0.0370021 | 0.00613799 | 1.66E-09 | 0.0138 | T | C |
| rs149809796 | 0.0284119 | 0.00490862 | 7.12E-09 | 0.023 | A | G |
| rs149841512 | -0.0408929 | 0.00501087 | 3.33E-16 | 0.0221 | A | G |
| rs149936105 | -0.0358673 | 0.0061173 | 4.54E-09 | 0.0323 | T | G |
| rs149980943 | 0.0710181 | 0.00616228 | 9.91E-31 | 0.0141 | T | C |
| rs150002928 | -0.0229451 | 0.0037336 | 7.97E-10 | 0.038 | T | G |
| rs150183551 | 0.0420942 | 0.00512767 | 2.23E-16 | 0.0215 | A | G |
| rs150272003 | 0.0459554 | 0.00638696 | 6.24E-13 | 0.0128 | G | T |
| rs150403348 | 0.0384153 | 0.00607372 | 2.53E-10 | 0.014 | A | G |
| rs150474434 | 0.0423421 | 0.00238341 | 1.31E-70 | 0.0949 | A | G |
| rs150595091 | 0.0338711 | 0.0054193 | 4.10E-10 | 0.0194 | C | T |
| rs150681312 | 0.0296881 | 0.00345049 | 7.70E-18 | 0.0427 | T | A |
| rs150688657 | -0.0215663 | 0.00231561 | 1.24E-20 | 0.103 | A | G |
| rs150750289 | 0.0383227 | 0.00530278 | 4.94E-13 | 0.0197 | T | C |
| rs150778385 | -0.0164754 | 0.00242202 | 1.03E-11 | 0.0915 | G | T |
| rs150837669 | -0.0400788 | 0.00572367 | 2.52E-12 | 0.0162 | C | T |
| rs150841483 | 0.0208631 | 0.00352152 | 3.13E-09 | 0.0418 | T | C |
| rs150888326 | -0.0285424 | 0.00382569 | 8.61E-14 | 0.0373 | T | C |
| rs150893163 | -0.0306169 | 0.0050804 | 1.68E-09 | 0.0203 | A | T |
| rs150958311 | 0.0349573 | 0.00511686 | 8.39E-12 | 0.0204 | T | G |
| rs150992660 | -0.0515195 | 0.00742941 | 4.08E-12 | 0.00912 | A | G |
| rs151006362 | -0.0127132 | 0.0020808 | 9.98E-10 | 0.128 | A | G |
| rs151009137 | 0.0426094 | 0.00774135 | 3.71E-08 | 0.00932 | A | G |
| rs151150389 | -0.0297667 | 0.00382611 | 7.26E-15 | 0.0337 | C | T |
| rs151191319 | 0.0308281 | 0.0038638 | 1.48E-15 | 0.0334 | A | G |
| rs151205730 | 0.0676985 | 0.00895132 | 3.94E-14 | 0.00641 | A | C |
| rs151218088 | -0.0443621 | 0.00787001 | 1.73E-08 | 0.0103 | T | C |
| rs151257922 | -0.0561556 | 0.00788355 | 1.05E-12 | 0.00839 | G | C |
| rs151264833 | -0.0339953 | 0.00458211 | 1.18E-13 | 0.0235 | G | C |
| rs151330717 | 0.30057 | 0.00586759 | 1.00E-200 | 0.0163 | A | G |
| rs1515565 | 0.00927654 | 0.00139218 | 2.68E-11 | 0.482 | A | G |
| rs151719 | 0.0109232 | 0.00169713 | 1.22E-10 | 0.232 | C | T |
| rs1525783 | -0.0120476 | 0.00154563 | 6.46E-15 | 0.294 | A | G |
| rs1526463 | -0.0132479 | 0.00190008 | 3.12E-12 | 0.193 | A | G |
| rs1530483 | 0.0132064 | 0.00197218 | 2.14E-11 | 0.161 | A | C |
| rs1531517 | 0.192545 | 0.00277244 | 1.00E-200 | 0.0659 | A | G |
| rs1532085 | -0.0106346 | 0.00141692 | 6.12E-14 | 0.386 | A | G |
| rs1534762 | 0.0104547 | 0.00172388 | 1.32E-09 | 0.37 | T | C |
| rs1552221 | 0.00992134 | 0.0015872 | 4.08E-10 | 0.267 | C | T |
| rs1556857 | 0.0168848 | 0.00140839 | 4.07E-33 | 0.407 | C | T |
| rs1560699 | -0.0607939 | 0.00798564 | 2.68E-14 | 0.00888 | T | C |
| rs1563177 | 0.0103647 | 0.00163821 | 2.50E-10 | 0.311 | A | T |
| rs1563293 | -0.0092169 | 0.00141333 | 6.96E-11 | 0.396 | A | G |
| rs1566451 | -0.0362638 | 0.00629872 | 8.55E-09 | 0.017 | T | C |
| rs1571791 | -0.0138569 | 0.00143531 | 4.71E-22 | 0.38 | T | C |
| rs157580 | 0.0975876 | 0.00143162 | 1.00E-200 | 0.381 | G | A |
| rs160838 | 0.0103639 | 0.00161732 | 1.47E-10 | 0.405 | G | A |
| rs1610095 | 0.0468548 | 0.00147262 | 1.00E-200 | 0.399 | C | G |
| rs1631174 | -0.0095622 | 0.00149661 | 1.67E-10 | 0.341 | A | C |
| rs163318 | -0.0145934 | 0.00140053 | 2.01E-25 | 0.475 | T | C |
| rs1634768 | 0.0106707 | 0.00164118 | 7.93E-11 | 0.449 | T | C |
| rs164205 | -0.0087371 | 0.0013913 | 3.39E-10 | 0.469 | T | C |
| rs165722 | 0.00910311 | 0.00140124 | 8.22E-11 | 0.471 | C | T |
| rs1664423 | 0.00867654 | 0.00151074 | 9.29E-09 | 0.394 | T | G |
| rs16844401 | -0.0237512 | 0.0028338 | 5.23E-17 | 0.0634 | A | G |
| rs16861497 | 0.00850125 | 0.00142443 | 2.40E-09 | 0.392 | T | G |
| rs1689801 | -0.0144266 | 0.00147894 | 1.76E-22 | 0.325 | A | G |
| rs1692119 | -0.0142554 | 0.00231555 | 7.44E-10 | 0.1 | T | C |
| rs16937506 | 0.0131043 | 0.00227956 | 9.00E-09 | 0.116 | C | G |
| rs16964261 | 0.0226321 | 0.00333371 | 1.13E-11 | 0.0503 | C | T |
| rs17035630 | -0.0434167 | 0.00220091 | 1.27E-86 | 0.121 | A | G |
| rs17036085 | 0.046122 | 0.00624447 | 1.51E-13 | 0.0123 | G | A |
| rs17050272 | 0.0228383 | 0.00144725 | 4.24E-56 | 0.421 | A | G |
| rs17053595 | -0.0141101 | 0.0025471 | 3.03E-08 | 0.0828 | A | G |
| rs17053830 | 0.0121421 | 0.001875 | 9.43E-11 | 0.165 | C | T |
| rs17054136 | -0.0333667 | 0.00314459 | 2.65E-26 | 0.0526 | A | G |
| rs17054348 | -0.0138422 | 0.002057 | 1.70E-11 | 0.139 | A | G |
| rs17111474 | -0.0113165 | 0.00163522 | 4.50E-12 | 0.296 | T | C |
| rs17111503 | -0.0487264 | 0.00160687 | 1.00E-200 | 0.256 | G | A |
| rs17111792 | -0.0320896 | 0.0031978 | 1.07E-23 | 0.0497 | G | A |
| rs17121798 | 0.0180307 | 0.00265553 | 1.12E-11 | 0.0745 | G | C |
| rs17144546 | 0.036213 | 0.00457041 | 2.31E-15 | 0.0259 | A | G |
| rs1718860 | -0.0095586 | 0.00157016 | 1.15E-09 | 0.267 | G | A |
| rs17206350 | -0.0181742 | 0.00145349 | 7.11E-36 | 0.454 | C | T |
| rs17242353 | -0.109071 | 0.00426378 | 2.49E-144 | 0.0324 | T | C |
| rs17242367 | -0.0282534 | 0.00289668 | 1.78E-22 | 0.0685 | T | C |
| rs17248748 | 0.0749455 | 0.00558649 | 4.91E-41 | 0.0177 | T | C |
| rs17249001 | -0.0714765 | 0.0029147 | 8.45E-133 | 0.0713 | A | G |
| rs1727743 | -0.0192919 | 0.00268873 | 7.23E-13 | 0.0806 | G | A |
| rs1729408 | 0.0178304 | 0.00149819 | 1.17E-32 | 0.338 | G | A |
| rs17305868 | -0.0138963 | 0.0013949 | 2.23E-23 | 0.477 | A | G |
| rs1736177 | -0.0155137 | 0.00152525 | 2.66E-24 | 0.307 | T | C |
| rs17381383 | -0.0228971 | 0.00191387 | 5.50E-33 | 0.17 | A | G |
| rs17395484 | -0.066936 | 0.00490063 | 1.79E-42 | 0.0215 | T | C |
| rs17406264 | 0.0325425 | 0.00532174 | 9.66E-10 | 0.0193 | T | C |
| rs17419403 | -0.0302545 | 0.0044898 | 1.60E-11 | 0.0259 | G | C |
| rs17427887 | -0.039177 | 0.00454117 | 6.29E-18 | 0.0278 | T | G |
| rs174481 | -0.0176468 | 0.00174316 | 4.35E-24 | 0.215 | G | C |
| rs174547 | 0.0429432 | 0.00145569 | 2.86E-191 | 0.346 | C | T |
| rs17476364 | 0.0268457 | 0.00233191 | 1.14E-30 | 0.0989 | C | T |
| rs17492613 | 0.02731 | 0.00446606 | 9.66E-10 | 0.0268 | C | T |
| rs17500468 | -0.0201899 | 0.00217588 | 1.71E-20 | 0.123 | G | A |
| rs17517088 | -0.0303203 | 0.00381894 | 2.03E-15 | 0.0416 | C | T |
| rs17528296 | -0.0379194 | 0.00258167 | 7.70E-49 | 0.0788 | T | C |
| rs17532371 | 0.0193589 | 0.00271228 | 9.50E-13 | 0.0706 | G | C |
| rs17554529 | 0.0231367 | 0.00384689 | 1.81E-09 | 0.036 | G | C |
| rs17562917 | 0.0187972 | 0.00288383 | 7.12E-11 | 0.0645 | A | G |
| rs17565182 | -0.0212692 | 0.00245145 | 4.09E-18 | 0.0912 | C | T |
| rs17580 | -0.0498131 | 0.00361468 | 3.33E-43 | 0.0387 | A | T |
| rs17617028 | -0.0132543 | 0.00167534 | 2.54E-15 | 0.217 | A | G |
| rs17636116 | -0.0217229 | 0.00382638 | 1.37E-08 | 0.0341 | T | C |
| rs17648121 | -0.0631082 | 0.00414886 | 2.99E-52 | 0.0307 | T | C |
| rs17652418 | 0.0119144 | 0.00176818 | 1.60E-11 | 0.199 | C | T |
| rs1766 | 0.0152369 | 0.00174211 | 2.21E-18 | 0.448 | G | A |
| rs17662231 | 0.0330186 | 0.00480201 | 6.16E-12 | 0.0228 | T | C |
| rs17665178 | 0.0158917 | 0.00156116 | 2.45E-24 | 0.302 | G | C |
| rs17666927 | 0.0719302 | 0.00477802 | 3.23E-51 | 0.0212 | A | G |
| rs17679693 | -0.0412401 | 0.00363915 | 9.07E-30 | 0.0421 | G | A |
| rs17681684 | -0.0092622 | 0.00148349 | 4.28E-10 | 0.32 | A | G |
| rs17690015 | -0.0142987 | 0.00178004 | 9.53E-16 | 0.223 | A | G |
| rs17699238 | -0.0264218 | 0.00174223 | 5.98E-52 | 0.198 | G | T |
| rs17703146 | -0.0112556 | 0.00195693 | 8.84E-09 | 0.162 | C | T |
| rs17710 | -0.0122866 | 0.00200822 | 9.47E-10 | 0.139 | T | A |
| rs17710008 | -0.0105495 | 0.00182995 | 8.17E-09 | 0.173 | A | G |
| rs17714718 | 0.00830382 | 0.00142912 | 6.23E-09 | 0.483 | C | T |
| rs17714761 | -0.0221421 | 0.0016413 | 1.78E-41 | 0.344 | T | A |
| rs17725246 | -0.0427777 | 0.00178857 | 2.03E-126 | 0.195 | C | T |
| rs17747311 | -0.0182384 | 0.00333283 | 4.44E-08 | 0.0451 | A | G |
| rs17793602 | -0.0157084 | 0.0019098 | 1.95E-16 | 0.158 | A | T |
| rs1782651 | -0.009192 | 0.00161529 | 1.27E-08 | 0.312 | T | C |
| rs17851257 | -0.0191881 | 0.00332668 | 8.02E-09 | 0.0508 | G | C |
| rs17853159 | 0.022428 | 0.00258562 | 4.17E-18 | 0.0778 | A | G |
| rs17875609 | 0.0656549 | 0.00506803 | 2.21E-38 | 0.0215 | T | C |
| rs17875621 | 0.0467274 | 0.00383323 | 3.51E-34 | 0.0475 | A | G |
| rs1800562 | 0.0571711 | 0.00276108 | 3.05E-95 | 0.0682 | A | G |
| rs1800777 | -0.0221128 | 0.00373621 | 3.25E-09 | 0.0356 | A | G |
| rs1800961 | 0.0549493 | 0.00385026 | 3.29E-46 | 0.0337 | T | C |
| rs1801689 | -0.0908315 | 0.00428983 | 1.67E-99 | 0.0261 | C | A |
| rs1807493 | -0.0089789 | 0.00163544 | 4.02E-08 | 0.236 | G | C |
| rs180755046 | 0.0421176 | 0.00623075 | 1.38E-11 | 0.0161 | A | C |
| rs1808919 | 0.0114425 | 0.00163071 | 2.27E-12 | 0.247 | G | A |
| rs1809165 | 0.0273787 | 0.00364837 | 6.17E-14 | 0.0402 | T | C |
| rs181331606 | -0.0286493 | 0.00297663 | 6.29E-22 | 0.0901 | G | C |
| rs181707894 | 0.0597213 | 0.00645686 | 2.26E-20 | 0.013 | T | C |
| rs181897168 | 0.0716585 | 0.00834309 | 8.78E-18 | 0.00852 | A | G |
| rs182201537 | -0.083522 | 0.00780687 | 1.03E-26 | 0.00937 | G | A |
| rs182491400 | -0.0444815 | 0.00555579 | 1.18E-15 | 0.0209 | T | C |
| rs182695896 | -0.0511225 | 0.0080956 | 2.70E-10 | 0.00809 | C | A |
| rs182824418 | -0.144427 | 0.0133126 | 2.02E-27 | 0.00296 | T | G |
| rs183191678 | -0.0470501 | 0.00733676 | 1.43E-10 | 0.00958 | A | G |
| rs1835346 | -0.0474328 | 0.00485997 | 1.67E-22 | 0.0211 | G | A |
| rs1836003 | -0.017067 | 0.00144232 | 2.63E-32 | 0.481 | C | T |
| rs183615662 | 0.036619 | 0.00669021 | 4.41E-08 | 0.0118 | A | G |
| rs1836278 | 0.0248226 | 0.00176726 | 8.17E-45 | 0.2 | C | T |
| rs1838931 | -0.0113437 | 0.00152716 | 1.10E-13 | 0.312 | T | C |
| rs1840144 | -0.0094833 | 0.00163563 | 6.71E-09 | 0.26 | A | T |
| rs184295022 | -0.033072 | 0.00604682 | 4.52E-08 | 0.0139 | T | G |
| rs1844754 | -0.0132381 | 0.00221531 | 2.29E-09 | 0.121 | G | A |
| rs184507838 | 0.125764 | 0.00686522 | 5.84E-75 | 0.0103 | T | C |
| rs184590748 | 0.0462955 | 0.00572627 | 6.23E-16 | 0.0157 | A | G |
| rs184829043 | -0.0674182 | 0.00818581 | 1.78E-16 | 0.0104 | T | C |
| rs184865012 | -0.0398664 | 0.00418399 | 1.60E-21 | 0.0437 | A | G |
| rs1850956 | 0.00823251 | 0.00141002 | 5.26E-09 | 0.494 | T | C |
| rs185161878 | -0.0177688 | 0.00320318 | 2.90E-08 | 0.0981 | C | G |
| rs185212438 | -0.0196831 | 0.00301739 | 6.88E-11 | 0.0619 | T | C |
| rs185630857 | 0.0267173 | 0.00463222 | 8.04E-09 | 0.0246 | A | C |
| rs1858879 | 0.0189636 | 0.00156428 | 7.99E-34 | 0.304 | C | G |
| rs185922357 | -0.0339639 | 0.00569051 | 2.39E-09 | 0.0166 | T | C |
| rs1862719 | -0.011164 | 0.00168573 | 3.53E-11 | 0.247 | A | G |
| rs186369086 | -0.0691973 | 0.00868126 | 1.58E-15 | 0.00687 | A | C |
| rs186696265 | -0.192744 | 0.00603933 | 1.00E-200 | 0.0135 | T | C |
| rs1867348 | 0.0184132 | 0.00247246 | 9.53E-14 | 0.0869 | T | C |
| rs1867727 | 0.0120836 | 0.00154121 | 4.49E-15 | 0.282 | C | G |
| rs186851194 | 0.046254 | 0.00404382 | 2.69E-30 | 0.0463 | G | C |
| rs1870140 | 0.0124748 | 0.00192407 | 8.96E-11 | 0.157 | A | G |
| rs187183066 | -0.0700427 | 0.00801637 | 2.38E-18 | 0.00964 | G | A |
| rs1871859 | -0.0184903 | 0.00206583 | 3.54E-19 | 0.13 | T | C |
| rs187356514 | -0.0507035 | 0.00633651 | 1.23E-15 | 0.0131 | C | T |
| rs187423 | 0.0139397 | 0.00193202 | 5.39E-13 | 0.154 | G | A |
| rs187429064 | 0.160458 | 0.00693095 | 1.42E-118 | 0.0117 | G | A |
| rs1877026 | -0.0191505 | 0.00196495 | 1.92E-22 | 0.156 | A | G |
| rs187870654 | 0.0637159 | 0.00718184 | 7.20E-19 | 0.011 | A | G |
| rs188031857 | 0.0488445 | 0.00715932 | 8.95E-12 | 0.011 | C | T |
| rs1881802 | 0.013786 | 0.00179815 | 1.76E-14 | 0.214 | G | A |
| rs1883225 | -0.0101348 | 0.00165316 | 8.76E-10 | 0.231 | G | A |
| rs1883931 | -0.0085915 | 0.00157517 | 4.92E-08 | 0.291 | T | C |
| rs188514851 | 0.0388018 | 0.0062379 | 4.96E-10 | 0.0133 | T | G |
| rs188778485 | -0.0339531 | 0.00463933 | 2.51E-13 | 0.024 | A | G |
| rs188993102 | -0.040006 | 0.00594706 | 1.73E-11 | 0.0159 | C | T |
| rs189143685 | -0.0494154 | 0.00688937 | 7.35E-13 | 0.0113 | T | C |
| rs1892252 | -0.0120486 | 0.00202121 | 2.51E-09 | 0.139 | G | C |
| rs1893351 | 0.0306531 | 0.00427511 | 7.49E-13 | 0.0287 | T | C |
| rs1905045 | -0.0103844 | 0.00180207 | 8.29E-09 | 0.202 | C | T |
| rs190564032 | -0.0793966 | 0.00896828 | 8.52E-19 | 0.0077 | A | G |
| rs191030430 | 0.0475978 | 0.00535835 | 6.51E-19 | 0.0187 | C | T |
| rs191320109 | -0.0445848 | 0.00688585 | 9.49E-11 | 0.0106 | T | C |
| rs191514719 | 0.0245716 | 0.00430073 | 1.11E-08 | 0.0306 | C | T |
| rs1917754 | 0.0279307 | 0.00191505 | 3.51E-48 | 0.201 | A | G |
| rs192542025 | 0.0333558 | 0.00544668 | 9.12E-10 | 0.0194 | A | G |
| rs193108398 | -0.050079 | 0.00805474 | 5.06E-10 | 0.00773 | A | G |
| rs193152187 | -0.0563374 | 0.00741278 | 2.96E-14 | 0.0128 | T | G |
| rs193171323 | 0.0442811 | 0.00683187 | 9.08E-11 | 0.0123 | G | A |
| rs1936789 | 0.0100201 | 0.00140418 | 9.61E-13 | 0.47 | G | A |
| rs1963676 | 0.0143783 | 0.00143027 | 8.92E-24 | 0.424 | C | T |
| rs1977658 | -0.013403 | 0.00160911 | 8.12E-17 | 0.333 | T | G |
| rs1980592 | -0.0144294 | 0.00219743 | 5.15E-11 | 0.145 | A | T |
| rs198464 | -0.0113907 | 0.00138959 | 2.46E-16 | 0.476 | A | G |
| rs1992172 | -0.019503 | 0.00175889 | 1.43E-28 | 0.194 | G | A |
| rs2011442 | 0.0120962 | 0.00148542 | 3.85E-16 | 0.339 | C | T |
| rs2015257 | 0.0299658 | 0.00172149 | 7.31E-68 | 0.25 | G | A |
| rs201763 | -0.0086694 | 0.00151665 | 1.09E-08 | 0.305 | G | T |
| rs2023222 | -0.0099792 | 0.00149573 | 2.53E-11 | 0.372 | G | T |
| rs2023472 | 0.00865778 | 0.00149451 | 6.91E-09 | 0.394 | A | G |
| rs2024628 | 0.013808 | 0.00165715 | 7.92E-17 | 0.367 | G | A |
| rs2039184 | -0.0147024 | 0.00139565 | 5.99E-26 | 0.435 | G | A |
| rs204480 | 0.0411663 | 0.00167205 | 7.67E-134 | 0.243 | T | C |
| rs204540 | 0.0235159 | 0.00168889 | 4.54E-44 | 0.223 | C | A |
| rs2049194 | 0.0135057 | 0.00165254 | 3.02E-16 | 0.228 | A | G |
| rs206014 | -0.0124176 | 0.0019382 | 1.49E-10 | 0.166 | G | A |
| rs2060658 | -0.0083996 | 0.00141416 | 2.86E-09 | 0.456 | T | C |
| rs206114 | -0.0076466 | 0.00140221 | 4.95E-08 | 0.427 | A | C |
| rs206321 | 0.0198717 | 0.00191821 | 3.79E-25 | 0.166 | C | A |
| rs2068888 | 0.0180327 | 0.00139568 | 3.45E-38 | 0.451 | A | G |
| rs207136 | 0.0246672 | 0.00203853 | 1.05E-33 | 0.136 | C | T |
| rs207139 | -0.0097215 | 0.00142945 | 1.04E-11 | 0.382 | G | A |
| rs2075801 | -0.0158027 | 0.00200685 | 3.42E-15 | 0.151 | A | G |
| rs208426 | -0.0113837 | 0.00150023 | 3.25E-14 | 0.326 | T | C |
| rs2085723 | 0.00980493 | 0.00145467 | 1.58E-11 | 0.351 | G | T |
| rs2088712 | -0.017104 | 0.00148708 | 1.29E-30 | 0.337 | T | C |
| rs2097775 | 0.0134012 | 0.00205248 | 6.61E-11 | 0.151 | A | T |
| rs2106115 | 0.00945139 | 0.00159667 | 3.23E-09 | 0.261 | T | C |
| rs212169 | -0.0262062 | 0.00414318 | 2.53E-10 | 0.031 | T | C |
| rs213499 | 0.0102293 | 0.00140202 | 2.96E-13 | 0.468 | C | A |
| rs214082 | 0.00790859 | 0.00141161 | 2.11E-08 | 0.415 | T | C |
| rs2158079 | 0.0201707 | 0.00234544 | 7.97E-18 | 0.108 | C | T |
| rs2165670 | -0.0169911 | 0.00223177 | 2.67E-14 | 0.112 | A | G |
| rs2168608 | 0.00896888 | 0.00142848 | 3.42E-10 | 0.493 | A | G |
| rs217370 | 0.0253687 | 0.00139315 | 4.33E-74 | 0.467 | G | A |
| rs2178464 | -0.0249314 | 0.00444702 | 2.07E-08 | 0.0247 | C | T |
| rs2186716 | -0.0162926 | 0.00265067 | 7.92E-10 | 0.076 | A | G |
| rs219562 | -0.0106309 | 0.00154019 | 5.12E-12 | 0.309 | G | A |
| rs2199403 | -0.0151952 | 0.00144401 | 6.77E-26 | 0.372 | C | T |
| rs2203474 | -0.0220758 | 0.00293389 | 5.30E-14 | 0.0708 | G | T |
| rs2205267 | 0.0301623 | 0.005021 | 1.89E-09 | 0.0202 | T | C |
| rs2206903 | -0.010575 | 0.00142492 | 1.16E-13 | 0.438 | A | G |
| rs2207132 | -0.124182 | 0.00410721 | 1.00E-200 | 0.0329 | A | G |
| rs2216502 | 0.0149431 | 0.00272318 | 4.08E-08 | 0.0785 | G | A |
| rs2228603 | 0.0883387 | 0.00263262 | 1.00E-200 | 0.0752 | T | C |
| rs223042 | -0.0116984 | 0.00201287 | 6.18E-09 | 0.136 | C | G |
| rs2235215 | 0.0318004 | 0.00148636 | 1.49E-101 | 0.321 | C | T |
| rs2235571 | -0.0186646 | 0.00269801 | 4.58E-12 | 0.0734 | G | T |
| rs2241233 | -0.0136297 | 0.00211486 | 1.16E-10 | 0.139 | C | T |
| rs2241476 | -0.0084446 | 0.00154076 | 4.23E-08 | 0.29 | C | A |
| rs2244458 | 0.0116405 | 0.00153515 | 3.39E-14 | 0.352 | T | C |
| rs2245094 | 0.0121932 | 0.00145425 | 5.09E-17 | 0.355 | T | G |
| rs2247139 | -0.0132285 | 0.00204579 | 1.00E-10 | 0.132 | A | G |
| rs2247213 | 0.0141452 | 0.00146523 | 4.73E-22 | 0.33 | A | G |
| rs2250751 | 0.0165291 | 0.00147712 | 4.56E-29 | 0.341 | A | G |
| rs2250781 | -0.0086542 | 0.00139816 | 6.03E-10 | 0.475 | C | A |
| rs2254968 | 0.00853064 | 0.00153669 | 2.84E-08 | 0.308 | G | A |
| rs2263637 | 0.0123172 | 0.00173744 | 1.35E-12 | 0.205 | C | T |
| rs226506 | 0.00931796 | 0.00144612 | 1.17E-10 | 0.391 | G | T |
| rs2267867 | 0.00934136 | 0.00164663 | 1.40E-08 | 0.236 | G | A |
| rs2270445 | -0.0141398 | 0.00140413 | 7.49E-24 | 0.483 | G | A |
| rs2274127 | 0.0105125 | 0.0014037 | 6.93E-14 | 0.474 | T | G |
| rs2287802 | -0.0097499 | 0.00150833 | 1.02E-10 | 0.371 | G | A |
| rs2288278 | 0.0144015 | 0.00146428 | 7.94E-23 | 0.35 | G | A |
| rs2290159 | 0.0213593 | 0.00167903 | 4.51E-37 | 0.216 | C | G |
| rs2295027 | -0.0115448 | 0.00152307 | 3.46E-14 | 0.322 | A | G |
| rs2296288 | 0.0113161 | 0.00140604 | 8.40E-16 | 0.437 | T | C |
| rs2297356 | 0.0183578 | 0.0020236 | 1.17E-19 | 0.135 | A | G |
| rs2300414 | -0.0227595 | 0.00266011 | 1.17E-17 | 0.0737 | A | G |
| rs2301179 | 0.0158997 | 0.00139539 | 4.46E-30 | 0.492 | A | G |
| rs2302434 | -0.0145292 | 0.00184432 | 3.33E-15 | 0.174 | T | C |
| rs2303152 | -0.0364765 | 0.00233186 | 3.73E-55 | 0.0971 | A | G |
| rs2304969 | 0.0111926 | 0.00197566 | 1.47E-08 | 0.151 | T | G |
| rs2305398 | -0.0083264 | 0.00142517 | 5.15E-09 | 0.387 | A | G |
| rs232925 | 0.0100393 | 0.00142598 | 1.92E-12 | 0.465 | T | C |
| rs2342307 | -0.0085904 | 0.00146879 | 4.96E-09 | 0.377 | A | G |
| rs2349805 | -0.0136103 | 0.00247233 | 3.69E-08 | 0.102 | C | G |
| rs2354155 | -0.0102054 | 0.00148656 | 6.64E-12 | 0.473 | G | A |
| rs2358957 | 0.0176618 | 0.00145199 | 4.84E-34 | 0.453 | C | A |
| rs236012 | -0.0155253 | 0.00165563 | 6.77E-21 | 0.229 | A | C |
| rs2374571 | 0.041801 | 0.00450701 | 1.78E-20 | 0.0278 | A | G |
| rs2384934 | -0.0086926 | 0.0015047 | 7.61E-09 | 0.342 | G | C |
| rs2385094 | 0.0152302 | 0.00212036 | 6.83E-13 | 0.124 | C | T |
| rs2390536 | -0.0219546 | 0.00145341 | 1.49E-51 | 0.36 | A | G |
| rs2391159 | 0.0234181 | 0.00171891 | 2.89E-42 | 0.207 | T | C |
| rs2394172 | -0.0235859 | 0.00286036 | 1.64E-16 | 0.0663 | T | C |
| rs2395471 | -0.0163239 | 0.00147495 | 1.81E-28 | 0.378 | A | G |
| rs2395943 | -0.0134641 | 0.00140162 | 7.54E-22 | 0.414 | A | G |
| rs2401637 | 0.00840697 | 0.00147223 | 1.13E-08 | 0.336 | T | C |
| rs240760 | 0.0123454 | 0.00158039 | 5.65E-15 | 0.344 | A | T |
| rs2413029 | -0.0096572 | 0.00162268 | 2.66E-09 | 0.244 | A | G |
| rs2413926 | 0.013495 | 0.00144702 | 1.10E-20 | 0.375 | A | T |
| rs2414759 | 0.00771995 | 0.00141258 | 4.63E-08 | 0.454 | C | T |
| rs2425412 | -0.0250608 | 0.00272465 | 3.65E-20 | 0.0817 | C | T |
| rs2425463 | 0.0175505 | 0.00211212 | 9.62E-17 | 0.141 | G | T |
| rs2428608 | 0.0118277 | 0.00140613 | 4.05E-17 | 0.435 | C | T |
| rs2429657 | -0.0111717 | 0.00174075 | 1.38E-10 | 0.228 | G | A |
| rs2434583 | -0.0081978 | 0.00145351 | 1.70E-08 | 0.469 | C | A |
| rs2442723 | 0.012661 | 0.00186538 | 1.14E-11 | 0.193 | A | G |
| rs2451322 | 0.0106423 | 0.00142294 | 7.48E-14 | 0.405 | G | A |
| rs2453464 | -0.0105687 | 0.00140998 | 6.60E-14 | 0.451 | A | G |
| rs2457574 | 0.014115 | 0.00140905 | 1.28E-23 | 0.414 | A | G |
| rs2458247 | 0.00792214 | 0.0014052 | 1.72E-08 | 0.443 | G | A |
| rs246184 | 0.0091036 | 0.00162425 | 2.08E-08 | 0.327 | C | G |
| rs2464895 | 0.0246155 | 0.00178295 | 2.34E-43 | 0.235 | C | A |
| rs247617 | 0.0366691 | 0.00148161 | 3.14E-135 | 0.321 | A | C |
| rs247791 | 0.0113296 | 0.00152318 | 1.02E-13 | 0.321 | C | G |
| rs2479408 | 0.0445077 | 0.00180457 | 2.61E-134 | 0.199 | G | C |
| rs2479420 | -0.0366939 | 0.00172956 | 6.83E-100 | 0.261 | C | T |
| rs2483719 | 0.00876841 | 0.00157541 | 2.61E-08 | 0.269 | T | C |
| rs2487039 | -0.0127139 | 0.0020593 | 6.66E-10 | 0.131 | T | C |
| rs2495477 | 0.0524078 | 0.00149171 | 1.00E-200 | 0.397 | G | A |
| rs2516079 | -0.0103971 | 0.00156568 | 3.12E-11 | 0.287 | G | T |
| rs2517671 | -0.0151548 | 0.00149689 | 4.32E-24 | 0.407 | G | A |
| rs2523659 | -0.0136435 | 0.00154689 | 1.15E-18 | 0.316 | G | T |
| rs2524292 | 0.0210265 | 0.00274309 | 1.78E-14 | 0.0686 | G | T |
| rs2526385 | 0.00995484 | 0.00178991 | 2.67E-08 | 0.188 | T | G |
| rs2543662 | -0.0086804 | 0.00154103 | 1.77E-08 | 0.282 | C | A |
| rs2547354 | -0.009013 | 0.00144396 | 4.32E-10 | 0.459 | T | C |
| rs257373 | 0.012746 | 0.00173903 | 2.31E-13 | 0.2 | A | G |
| rs2587538 | -0.0206314 | 0.00328296 | 3.29E-10 | 0.0577 | A | G |
| rs2590990 | 0.0131191 | 0.00162913 | 8.09E-16 | 0.248 | G | A |
| rs260422 | 0.00960964 | 0.001424 | 1.50E-11 | 0.384 | C | T |
| rs261342 | -0.0141447 | 0.00171247 | 1.46E-16 | 0.215 | G | C |
| rs2617447 | -0.0084492 | 0.00140646 | 1.88E-09 | 0.426 | G | A |
| rs2618566 | -0.040212 | 0.00151102 | 4.87E-156 | 0.339 | G | T |
| rs2621321 | -0.019023 | 0.00164464 | 6.08E-31 | 0.257 | G | A |
| rs2627646 | -0.0387057 | 0.00301396 | 9.52E-38 | 0.0682 | A | G |
| rs263423 | 0.0106027 | 0.00175555 | 1.55E-09 | 0.201 | A | G |
| rs2640516 | -0.0083763 | 0.00148167 | 1.57E-08 | 0.323 | A | G |
| rs2642422 | 0.00872127 | 0.00151908 | 9.40E-09 | 0.298 | T | C |
| rs2642438 | 0.0268921 | 0.00152599 | 1.65E-69 | 0.294 | A | G |
| rs2660373 | -0.0128321 | 0.00197066 | 7.44E-11 | 0.155 | C | G |
| rs2664593 | 0.00962307 | 0.00174615 | 3.57E-08 | 0.21 | G | C |
| rs2673960 | 0.0123808 | 0.00217928 | 1.34E-08 | 0.119 | T | C |
| rs267733 | 0.0187116 | 0.00190325 | 8.25E-23 | 0.156 | G | A |
| rs268132 | 0.00804468 | 0.00138913 | 6.99E-09 | 0.444 | C | T |
| rs2686344 | -0.0092384 | 0.00164089 | 1.80E-08 | 0.24 | T | C |
| rs2700299 | -0.0119092 | 0.00211389 | 1.76E-08 | 0.123 | C | T |
| rs2702515 | -0.0086792 | 0.00144838 | 2.07E-09 | 0.355 | T | C |
| rs2715825 | -0.0104572 | 0.00152754 | 7.61E-12 | 0.346 | T | G |
| rs2721948 | -0.0091891 | 0.001437 | 1.61E-10 | 0.383 | C | T |
| rs272441 | -0.0137103 | 0.00245056 | 2.21E-08 | 0.0899 | A | G |
| rs2727261 | 0.0196389 | 0.00231004 | 1.87E-17 | 0.103 | T | C |
| rs272895 | 0.0153506 | 0.00202059 | 3.03E-14 | 0.14 | G | A |
| rs2737245 | 0.0253718 | 0.00155136 | 4.04E-60 | 0.277 | T | G |
| rs2738456 | 0.0253519 | 0.00148364 | 1.83E-65 | 0.325 | C | T |
| rs2740488 | 0.0249217 | 0.00158221 | 6.74E-56 | 0.261 | C | A |
| rs2745803 | -0.0134893 | 0.00177616 | 3.09E-14 | 0.2 | G | A |
| rs2777798 | 0.0145462 | 0.002344 | 5.44E-10 | 0.0985 | A | G |
| rs278940 | 0.00917831 | 0.00145999 | 3.25E-10 | 0.35 | A | G |
| rs2791948 | -0.0124172 | 0.00186835 | 3.01E-11 | 0.175 | C | G |
| rs2792751 | -0.023866 | 0.00153005 | 7.49E-55 | 0.281 | T | C |
| rs2802861 | 0.019393 | 0.00177714 | 1.00E-27 | 0.197 | C | T |
| rs2802930 | 0.015167 | 0.00260206 | 5.58E-09 | 0.0843 | C | T |
| rs2802959 | -0.0154276 | 0.00230964 | 2.40E-11 | 0.116 | G | A |
| rs2807837 | 0.0163604 | 0.00228482 | 8.04E-13 | 0.118 | G | A |
| rs2820232 | 0.0106669 | 0.00140528 | 3.18E-14 | 0.445 | T | G |
| rs2833487 | -0.0314967 | 0.00321288 | 1.09E-22 | 0.0494 | G | A |
| rs28362819 | -0.0260622 | 0.00391071 | 2.66E-11 | 0.0443 | T | C |
| rs28362834 | 0.0213086 | 0.00254295 | 5.32E-17 | 0.0832 | G | C |
| rs283813 | 0.139884 | 0.00276289 | 1.00E-200 | 0.0686 | A | T |
| rs28381981 | -0.0220147 | 0.0028654 | 1.55E-14 | 0.0624 | A | G |
| rs28385715 | -0.0455686 | 0.00489729 | 1.34E-20 | 0.0229 | G | T |
| rs28391744 | -0.0095321 | 0.00171274 | 2.62E-08 | 0.226 | T | C |
| rs28393611 | 0.0126857 | 0.00163862 | 9.81E-15 | 0.286 | T | C |
| rs28399607 | -0.0935466 | 0.00334904 | 1.08E-171 | 0.0532 | C | T |
| rs28399637 | -0.0850305 | 0.00160968 | 1.00E-200 | 0.316 | A | G |
| rs28399664 | -0.16198 | 0.00526927 | 1.00E-200 | 0.0225 | G | C |
| rs28399665 | 0.449253 | 0.0101868 | 1.00E-200 | 0.00506 | T | C |
| rs2840354 | -0.011566 | 0.00177873 | 7.91E-11 | 0.191 | T | C |
| rs28461471 | -0.0221249 | 0.00256887 | 7.14E-18 | 0.0957 | G | A |
| rs28463601 | -0.0179616 | 0.00285156 | 3.00E-10 | 0.0791 | A | G |
| rs28471982 | 0.00915595 | 0.00144176 | 2.15E-10 | 0.372 | G | A |
| rs28483956 | 0.0465627 | 0.00314195 | 1.09E-49 | 0.052 | A | G |
| rs28497720 | 0.0175436 | 0.00159642 | 4.30E-28 | 0.251 | T | C |
| rs28498684 | -0.0131553 | 0.00141998 | 1.96E-20 | 0.4 | A | G |
| rs28515937 | -0.0186171 | 0.00227995 | 3.20E-16 | 0.102 | T | C |
| rs2854153 | 0.0093006 | 0.00159889 | 5.99E-09 | 0.27 | T | C |
| rs2854640 | -0.0094311 | 0.00170816 | 3.37E-08 | 0.223 | A | G |
| rs28573770 | -0.059188 | 0.00781011 | 3.50E-14 | 0.00856 | A | T |
| rs2858211 | -0.0111624 | 0.00178628 | 4.13E-10 | 0.21 | T | C |
| rs28594937 | -0.0216371 | 0.00358309 | 1.55E-09 | 0.0403 | A | G |
| rs28658260 | -0.0085311 | 0.00150408 | 1.41E-08 | 0.314 | G | A |
| rs28659908 | 0.00997026 | 0.0014626 | 9.31E-12 | 0.402 | C | T |
| rs28666039 | -0.0105714 | 0.00143555 | 1.78E-13 | 0.377 | C | G |
| rs28745907 | 0.0190209 | 0.00187041 | 2.72E-24 | 0.178 | G | T |
| rs28811342 | -0.0125833 | 0.0017499 | 6.44E-13 | 0.197 | C | T |
| rs28832306 | -0.0208096 | 0.00180543 | 9.74E-31 | 0.18 | T | A |
| rs28875187 | -0.0094661 | 0.00160574 | 3.74E-09 | 0.254 | T | C |
| rs28894982 | -0.0234809 | 0.0033244 | 1.63E-12 | 0.0504 | T | C |
| rs2891761 | 0.0117645 | 0.00212902 | 3.28E-08 | 0.12 | C | T |
| rs28929474 | -0.0527623 | 0.00518309 | 2.44E-24 | 0.018 | T | C |
| rs2893236 | -0.0128716 | 0.00168858 | 2.48E-14 | 0.218 | C | T |
| rs289718 | 0.0119231 | 0.00150865 | 2.72E-15 | 0.311 | C | T |
| rs2899687 | 0.00897673 | 0.00163819 | 4.26E-08 | 0.243 | C | T |
| rs2928576 | 0.0119763 | 0.00147962 | 5.77E-16 | 0.328 | T | C |
| rs2933547 | -0.0085741 | 0.00143586 | 2.35E-09 | 0.417 | G | A |
| rs293435 | -0.0149218 | 0.00156797 | 1.79E-21 | 0.291 | T | C |
| rs2941465 | 0.00842039 | 0.00139864 | 1.74E-09 | 0.437 | T | C |
| rs2954013 | 0.00839964 | 0.00145289 | 7.41E-09 | 0.46 | G | A |
| rs2954720 | -0.0124306 | 0.00156908 | 2.33E-15 | 0.387 | G | T |
| rs2965106 | 0.042837 | 0.00149044 | 1.17E-181 | 0.33 | G | A |
| rs2965109 | 0.045604 | 0.00145751 | 1.00E-200 | 0.36 | T | C |
| rs2965113 | 0.0838149 | 0.00219468 | 1.00E-200 | 0.118 | C | T |
| rs2965167 | 0.0163342 | 0.00146368 | 6.42E-29 | 0.493 | G | A |
| rs2965174 | 0.0259062 | 0.0013954 | 6.12E-77 | 0.473 | A | G |
| rs2970901 | -0.0096909 | 0.00140968 | 6.22E-12 | 0.459 | T | G |
| rs2972831 | -0.0169265 | 0.00224209 | 4.37E-14 | 0.11 | T | C |
| rs2980874 | 0.017374 | 0.00149622 | 3.58E-31 | 0.388 | A | G |
| rs2992647 | 0.0117576 | 0.00141303 | 8.73E-17 | 0.408 | G | A |
| rs2992752 | -0.0101005 | 0.00142901 | 1.57E-12 | 0.37 | A | C |
| rs299653 | 0.00875115 | 0.00143327 | 1.02E-09 | 0.39 | C | G |
| rs3005923 | 0.074948 | 0.00796253 | 4.84E-21 | 0.00958 | A | G |
| rs3010275 | 0.0183321 | 0.001703 | 5.06E-27 | 0.21 | G | T |
| rs307586 | -0.0184922 | 0.00224178 | 1.60E-16 | 0.111 | C | T |
| rs3093602 | 0.0308326 | 0.00373223 | 1.44E-16 | 0.0513 | A | G |
| rs3095304 | 0.0141554 | 0.00181461 | 6.15E-15 | 0.189 | T | C |
| rs3101823 | -0.0274265 | 0.00211607 | 2.03E-38 | 0.124 | G | T |
| rs3107295 | 0.00840447 | 0.00144328 | 5.77E-09 | 0.406 | G | A |
| rs3112438 | 0.0386456 | 0.00167361 | 5.68E-118 | 0.233 | G | A |
| rs3112439 | -0.0293359 | 0.00231746 | 1.00E-36 | 0.126 | G | C |
| rs312033 | 0.0445033 | 0.00149421 | 6.33E-195 | 0.318 | A | G |
| rs3124747 | -0.0169257 | 0.00158377 | 1.17E-26 | 0.326 | A | G |
| rs3124775 | 0.0136508 | 0.00163853 | 8.01E-17 | 0.434 | G | A |
| rs3130573 | -0.0087403 | 0.00150544 | 6.41E-09 | 0.338 | G | A |
| rs3132790 | 0.00811112 | 0.00142523 | 1.26E-08 | 0.426 | T | C |
| rs316036 | 0.0202821 | 0.00140303 | 2.30E-47 | 0.477 | A | G |
| rs3184504 | 0.0234144 | 0.00139369 | 2.43E-63 | 0.473 | T | C |
| rs319454 | 0.00870142 | 0.00155084 | 2.01E-08 | 0.285 | A | G |
| rs3212243 | 0.00963986 | 0.00159313 | 1.44E-09 | 0.255 | C | T |
| rs3212930 | 0.0194876 | 0.00175491 | 1.19E-28 | 0.2 | G | A |
| rs333930 | -0.0077067 | 0.00141037 | 4.65E-08 | 0.433 | A | G |
| rs337171 | 0.0116622 | 0.00209175 | 2.47E-08 | 0.14 | G | A |
| rs33967773 | 0.0103678 | 0.00144263 | 6.64E-13 | 0.356 | T | C |
| rs34006803 | -0.0138303 | 0.00228997 | 1.55E-09 | 0.1 | A | G |
| rs34006806 | 0.0190104 | 0.00200365 | 2.36E-21 | 0.143 | A | G |
| rs34046594 | 0.0148504 | 0.00189188 | 4.18E-15 | 0.163 | G | C |
| rs34054295 | -0.0117815 | 0.00147139 | 1.17E-15 | 0.383 | A | G |
| rs340615 | -0.0308602 | 0.00376295 | 2.38E-16 | 0.0387 | A | T |
| rs34061869 | 0.0193358 | 0.00277403 | 3.16E-12 | 0.0683 | A | G |
| rs34243815 | 0.0231589 | 0.00292873 | 2.63E-15 | 0.0626 | T | C |
| rs342467 | 0.0101553 | 0.00141921 | 8.33E-13 | 0.399 | T | C |
| rs34253960 | -0.0106053 | 0.00154091 | 5.88E-12 | 0.474 | G | A |
| rs34265667 | 0.0244429 | 0.00400661 | 1.06E-09 | 0.0311 | A | G |
| rs34287227 | -0.0146926 | 0.00258525 | 1.32E-08 | 0.0839 | T | C |
| rs34342452 | 0.0096502 | 0.00175924 | 4.12E-08 | 0.209 | G | C |
| rs34366352 | -0.0157009 | 0.00217591 | 5.36E-13 | 0.118 | C | T |
| rs34372369 | -0.0192832 | 0.00310689 | 5.41E-10 | 0.0508 | A | G |
| rs34381009 | -0.0130446 | 0.00193355 | 1.52E-11 | 0.153 | G | A |
| rs34438598 | -0.0137497 | 0.00249363 | 3.51E-08 | 0.092 | T | G |
| rs34448858 | -0.0500321 | 0.00579418 | 5.88E-18 | 0.0166 | A | G |
| rs34460487 | -0.0146432 | 0.0014964 | 1.30E-22 | 0.336 | A | G |
| rs34466729 | -0.0144835 | 0.00213481 | 1.17E-11 | 0.124 | G | C |
| rs34468875 | -0.0799415 | 0.00140566 | 1.00E-200 | 0.429 | T | C |
| rs34489489 | 0.0115732 | 0.00186875 | 5.90E-10 | 0.166 | C | T |
| rs34528974 | 0.022313 | 0.00384349 | 6.42E-09 | 0.0382 | A | G |
| rs34550071 | -0.0171412 | 0.00231242 | 1.24E-13 | 0.102 | C | G |
| rs34561079 | -0.0164845 | 0.0023424 | 1.96E-12 | 0.108 | A | G |
| rs34583389 | 0.0216956 | 0.00166379 | 7.26E-39 | 0.419 | T | C |
| rs34592756 | 0.0361265 | 0.00238626 | 8.90E-52 | 0.0946 | G | A |
| rs34685754 | 0.012352 | 0.00143874 | 9.06E-18 | 0.39 | A | G |
| rs34706906 | 0.0156949 | 0.0024122 | 7.69E-11 | 0.097 | T | C |
| rs34707604 | -0.0317679 | 0.00186917 | 8.83E-65 | 0.248 | C | T |
| rs34752362 | 0.00964234 | 0.00141299 | 8.85E-12 | 0.443 | A | G |
| rs34827707 | 0.2923 | 0.00488162 | 1.00E-200 | 0.0241 | A | G |
| rs34832185 | 0.0115253 | 0.00178329 | 1.03E-10 | 0.184 | C | T |
| rs34927947 | -0.0113828 | 0.00142516 | 1.38E-15 | 0.451 | G | A |
| rs34931250 | -0.0340497 | 0.00298053 | 3.17E-30 | 0.0599 | T | C |
| rs34936125 | -0.0145161 | 0.00211165 | 6.23E-12 | 0.247 | T | A |
| rs34947586 | 0.0103301 | 0.00179336 | 8.40E-09 | 0.188 | T | C |
| rs35057129 | -0.0351828 | 0.00181703 | 1.59E-83 | 0.296 | G | A |
| rs35063109 | -0.0302459 | 0.00342637 | 1.07E-18 | 0.0455 | A | C |
| rs35081008 | 0.0308607 | 0.0019307 | 1.65E-57 | 0.155 | T | C |
| rs35106682 | -0.0078202 | 0.00139458 | 2.05E-08 | 0.493 | A | C |
| rs35116854 | -0.0180351 | 0.00310543 | 6.34E-09 | 0.0519 | C | A |
| rs35122945 | 0.0370552 | 0.00297073 | 1.04E-35 | 0.0674 | C | A |
| rs35141185 | 0.0291475 | 0.00417391 | 2.88E-12 | 0.0318 | A | G |
| rs35166226 | 0.0206148 | 0.00181274 | 5.75E-30 | 0.183 | C | T |
| rs35271870 | 0.11706 | 0.00251606 | 1.00E-200 | 0.0836 | C | T |
| rs352942 | 0.0104223 | 0.00163487 | 1.83E-10 | 0.252 | A | G |
| rs35384424 | 0.061092 | 0.00340218 | 4.25E-72 | 0.0469 | T | C |
| rs35408229 | -0.0165582 | 0.00249995 | 3.51E-11 | 0.19 | T | A |
| rs35428152 | 0.0120843 | 0.00169918 | 1.15E-12 | 0.216 | T | C |
| rs35468353 | -0.0118798 | 0.00143087 | 1.02E-16 | 0.377 | G | A |
| rs35483143 | -0.0297664 | 0.00539237 | 3.39E-08 | 0.0193 | T | A |
| rs35678264 | 0.020395 | 0.00337701 | 1.55E-09 | 0.048 | A | G |
| rs35802157 | -0.0097029 | 0.00144441 | 1.85E-11 | 0.373 | T | C |
| rs35824797 | -0.0184819 | 0.00261914 | 1.71E-12 | 0.0776 | T | C |
| rs35836101 | 0.0391452 | 0.00157086 | 4.56E-137 | 0.288 | A | G |
| rs35882350 | -0.0181651 | 0.00162585 | 5.55E-29 | 0.256 | G | A |
| rs35901991 | -0.009206 | 0.00164184 | 2.06E-08 | 0.247 | T | C |
| rs35929534 | 0.178615 | 0.0081622 | 3.76E-106 | 0.00943 | C | G |
| rs36014207 | 0.0108883 | 0.00151581 | 6.81E-13 | 0.407 | T | C |
| rs36030967 | 0.0141134 | 0.00183653 | 1.53E-14 | 0.24 | G | T |
| rs36049922 | -0.0436966 | 0.0039585 | 2.49E-28 | 0.0347 | C | T |
| rs36134738 | 0.0889627 | 0.00277044 | 1.00E-200 | 0.0698 | G | A |
| rs365653 | 0.184154 | 0.00225335 | 1.00E-200 | 0.111 | G | A |
| rs367561550 | 0.0351301 | 0.00624585 | 1.86E-08 | 0.0168 | A | G |
| rs3731249 | 0.0229229 | 0.00416735 | 3.79E-08 | 0.0282 | T | C |
| rs3732356 | -0.0198072 | 0.00279879 | 1.47E-12 | 0.0678 | G | T |
| rs3732359 | -0.0148565 | 0.00164429 | 1.64E-19 | 0.23 | G | A |
| rs3735400 | 0.0153254 | 0.00214829 | 9.77E-13 | 0.117 | G | C |
| rs374459115 | -0.068345 | 0.006762 | 5.13E-24 | 0.0172 | A | G |
| rs3745706 | 0.0100563 | 0.00164815 | 1.05E-09 | 0.308 | T | C |
| rs3746778 | 0.0130201 | 0.00158782 | 2.40E-16 | 0.402 | A | G |
| rs3747207 | 0.0139089 | 0.00166675 | 7.13E-17 | 0.222 | A | G |
| rs3750321 | -0.0100829 | 0.00162876 | 6.00E-10 | 0.305 | G | T |
| rs3751973 | 0.0170964 | 0.00286213 | 2.32E-09 | 0.0651 | A | T |
| rs3754184 | -0.0088533 | 0.00147618 | 2.00E-09 | 0.373 | G | C |
| rs3754459 | 0.0175186 | 0.00319506 | 4.18E-08 | 0.0487 | C | T |
| rs3761740 | -0.0536811 | 0.00233111 | 2.44E-117 | 0.0971 | A | C |
| rs3764613 | -0.0110015 | 0.00140495 | 4.86E-15 | 0.436 | A | G |
| rs3764941 | -0.0083675 | 0.00150955 | 2.97E-08 | 0.3 | G | T |
| rs3773364 | -0.0116988 | 0.00191133 | 9.31E-10 | 0.166 | G | A |
| rs3780181 | 0.0358747 | 0.0027624 | 1.45E-38 | 0.0696 | G | A |
| rs3780190 | -0.0112615 | 0.00141489 | 1.73E-15 | 0.467 | A | G |
| rs3784924 | 0.0132798 | 0.00151895 | 2.28E-18 | 0.317 | G | A |
| rs379309 | -0.0251615 | 0.00142675 | 1.31E-69 | 0.495 | C | T |
| rs3800393 | -0.0137571 | 0.00180785 | 2.75E-14 | 0.18 | C | T |
| rs3800461 | 0.0244376 | 0.00213816 | 2.99E-30 | 0.119 | C | G |
| rs3807039 | -0.0184121 | 0.00230171 | 1.25E-15 | 0.109 | C | A |
| rs3809113 | -0.0098835 | 0.00157564 | 3.55E-10 | 0.287 | C | G |
| rs3820897 | -0.0159195 | 0.00176923 | 2.30E-19 | 0.192 | T | C |
| rs3823151 | -0.024107 | 0.00390842 | 6.92E-10 | 0.0319 | C | A |
| rs3826408 | 0.0147995 | 0.00140895 | 8.29E-26 | 0.463 | T | C |
| rs3826810 | -0.0375748 | 0.0034252 | 5.32E-28 | 0.0425 | A | G |
| rs3826994 | -0.0110289 | 0.00189152 | 5.52E-09 | 0.171 | G | T |
| rs3827503 | 0.0124539 | 0.00158672 | 4.20E-15 | 0.259 | T | A |
| rs3843763 | 0.0120459 | 0.00156617 | 1.46E-14 | 0.265 | T | C |
| rs3845301 | -0.0085249 | 0.00142739 | 2.34E-09 | 0.395 | C | A |
| rs3862606 | -0.0099099 | 0.00143578 | 5.12E-12 | 0.448 | G | A |
| rs3864294 | 0.00923477 | 0.00149339 | 6.26E-10 | 0.381 | G | A |
| rs386453 | -0.0350569 | 0.00229452 | 1.06E-52 | 0.104 | C | T |
| rs3869097 | -0.0120574 | 0.00154826 | 6.82E-15 | 0.324 | T | C |
| rs3890746 | 0.0098264 | 0.0013986 | 2.13E-12 | 0.426 | C | T |
| rs3916856 | -0.0291729 | 0.00418752 | 3.25E-12 | 0.0305 | A | G |
| rs39297 | 0.0195678 | 0.00197224 | 3.35E-23 | 0.151 | T | C |
| rs3935011 | -0.0089372 | 0.00140019 | 1.74E-10 | 0.48 | C | T |
| rs4006563 | -0.0252088 | 0.0031993 | 3.29E-15 | 0.0743 | C | T |
| rs400722 | 0.0130693 | 0.00208349 | 3.55E-10 | 0.138 | C | A |
| rs400824 | -0.0103308 | 0.00154869 | 2.55E-11 | 0.29 | T | C |
| rs402348 | 0.0111643 | 0.00174531 | 1.59E-10 | 0.205 | G | T |
| rs4044514 | 0.0170121 | 0.00172301 | 5.43E-23 | 0.304 | C | T |
| rs407258 | -0.0103803 | 0.00161054 | 1.15E-10 | 0.25 | C | T |
| rs4074793 | -0.0211877 | 0.00259359 | 3.10E-16 | 0.0751 | G | A |
| rs41264844 | 0.0332866 | 0.00429595 | 9.31E-15 | 0.0269 | T | C |
| rs41265924 | 0.0389939 | 0.00354486 | 3.82E-28 | 0.0403 | G | A |
| rs41272114 | 0.026269 | 0.00363065 | 4.64E-13 | 0.0386 | T | C |
| rs41279684 | -0.0257416 | 0.00193563 | 2.35E-40 | 0.152 | C | T |
| rs41280463 | 0.0152278 | 0.00185837 | 2.52E-16 | 0.167 | A | G |
| rs41280663 | 0.013224 | 0.00211202 | 3.82E-10 | 0.135 | T | C |
| rs41290100 | 0.0424322 | 0.00471008 | 2.08E-19 | 0.0251 | T | C |
| rs41294821 | 0.0427234 | 0.00501678 | 1.65E-17 | 0.0206 | T | C |
| rs41294825 | 0.0414044 | 0.00341298 | 7.20E-34 | 0.043 | T | A |
| rs41297885 | 0.0406301 | 0.00378858 | 7.82E-27 | 0.0358 | G | C |
| rs41299561 | 0.0421479 | 0.00665307 | 2.37E-10 | 0.0115 | C | T |
| rs41311276 | 0.0142915 | 0.00184046 | 8.15E-15 | 0.175 | C | A |
| rs4131228 | -0.0414626 | 0.00516994 | 1.06E-15 | 0.0208 | G | A |
| rs41329344 | -0.0137729 | 0.00223775 | 7.52E-10 | 0.107 | T | C |
| rs413582 | -0.0375714 | 0.00143903 | 2.89E-150 | 0.477 | T | C |
| rs413733 | -0.0125871 | 0.0016697 | 4.75E-14 | 0.218 | G | A |
| rs4142036 | -0.013088 | 0.00227838 | 9.22E-09 | 0.107 | T | C |
| rs4143973 | 0.008944 | 0.00139957 | 1.65E-10 | 0.491 | C | T |
| rs4147973 | -0.0082789 | 0.0014359 | 8.13E-09 | 0.468 | G | A |
| rs4148216 | 0.0280503 | 0.0018566 | 1.43E-51 | 0.169 | T | C |
| rs4149308 | -0.0209341 | 0.00214885 | 2.00E-22 | 0.117 | T | C |
| rs415023 | -0.0094541 | 0.00164439 | 8.96E-09 | 0.255 | A | G |
| rs42122 | -0.0124411 | 0.00190428 | 6.44E-11 | 0.165 | A | G |
| rs4216 | -0.0117487 | 0.00160176 | 2.22E-13 | 0.333 | T | C |
| rs4239702 | 0.0112221 | 0.00154047 | 3.22E-13 | 0.28 | T | C |
| rs4251841 | 0.00888753 | 0.00162479 | 4.50E-08 | 0.476 | C | T |
| rs427236 | 0.00910663 | 0.00143422 | 2.16E-10 | 0.463 | T | C |
| rs4280262 | -0.0098885 | 0.00174465 | 1.45E-08 | 0.206 | C | T |
| rs4284750 | 0.0132308 | 0.00149764 | 1.01E-18 | 0.328 | A | G |
| rs42854 | -0.0447595 | 0.00149898 | 6.52E-196 | 0.311 | G | C |
| rs4299376 | -0.0700085 | 0.00150787 | 1.00E-200 | 0.309 | G | T |
| rs4329504 | 0.00822839 | 0.00143423 | 9.63E-09 | 0.42 | G | A |
| rs4371007 | 0.0132846 | 0.00229761 | 7.39E-09 | 0.105 | T | C |
| rs4374942 | -0.0199517 | 0.00255281 | 5.47E-15 | 0.0808 | C | T |
| rs4379435 | 0.0112435 | 0.0014129 | 1.75E-15 | 0.431 | C | T |
| rs438129 | -0.0114221 | 0.00169487 | 1.59E-11 | 0.218 | T | C |
| rs438568 | 0.0135404 | 0.0014217 | 1.66E-21 | 0.392 | A | G |
| rs4390169 | -0.0119697 | 0.00138496 | 5.49E-18 | 0.485 | A | G |
| rs443081 | -0.0141533 | 0.00203271 | 3.34E-12 | 0.134 | T | C |
| rs4455790 | 0.00941949 | 0.00162039 | 6.13E-09 | 0.272 | C | G |
| rs4465730 | -0.0146047 | 0.00167914 | 3.39E-18 | 0.219 | A | G |
| rs4480845 | -0.0087604 | 0.00146995 | 2.53E-09 | 0.366 | T | C |
| rs4489379 | 0.0246447 | 0.00148254 | 4.73E-62 | 0.391 | C | T |
| rs45461994 | 0.0174505 | 0.00278526 | 3.72E-10 | 0.073 | T | C |
| rs45478293 | -0.0457001 | 0.00679429 | 1.74E-11 | 0.0108 | T | C |
| rs4551181 | 0.00889356 | 0.00161265 | 3.49E-08 | 0.254 | C | T |
| rs45580533 | 0.0370588 | 0.00490825 | 4.34E-14 | 0.0213 | G | A |
| rs45587445 | 0.0406411 | 0.00568437 | 8.70E-13 | 0.015 | T | C |
| rs45613837 | 0.00793402 | 0.00142815 | 2.77E-08 | 0.451 | A | G |
| rs4564699 | 0.0202044 | 0.00196945 | 1.08E-24 | 0.153 | A | G |
| rs4576078 | 0.0125105 | 0.00215814 | 6.76E-09 | 0.2 | T | C |
| rs4585645 | 0.0158003 | 0.00182371 | 4.56E-18 | 0.187 | T | C |
| rs4609107 | -0.009124 | 0.00158094 | 7.87E-09 | 0.265 | G | T |
| rs4621631 | -0.0135509 | 0.00144126 | 5.35E-21 | 0.378 | C | T |
| rs4632263 | 0.00918623 | 0.00161198 | 1.21E-08 | 0.272 | T | C |
| rs4639966 | -0.0147037 | 0.00161732 | 9.78E-20 | 0.241 | C | T |
| rs4640292 | -0.0129076 | 0.00148173 | 3.01E-18 | 0.326 | C | G |
| rs4645567 | -0.0141477 | 0.00171135 | 1.37E-16 | 0.214 | T | A |
| rs4646276 | 0.0245534 | 0.00256297 | 9.70E-22 | 0.0822 | A | G |
| rs4671050 | 0.0223969 | 0.0015036 | 3.52E-50 | 0.32 | T | G |
| rs4684859 | -0.0137168 | 0.00139838 | 1.03E-22 | 0.422 | A | G |
| rs4689653 | 0.0112429 | 0.00143992 | 5.81E-15 | 0.383 | T | G |
| rs4690087 | 0.019489 | 0.00337418 | 7.65E-09 | 0.0494 | A | G |
| rs469882 | 0.0100106 | 0.0017401 | 8.77E-09 | 0.199 | C | A |
| rs4713979 | -0.0124914 | 0.00166041 | 5.35E-14 | 0.249 | T | C |
| rs4719841 | 0.0199765 | 0.00141472 | 2.84E-45 | 0.4 | G | A |
| rs4719925 | -0.0133769 | 0.00237273 | 1.72E-08 | 0.092 | G | A |
| rs4722551 | -0.0361336 | 0.00186483 | 1.22E-83 | 0.163 | C | T |
| rs4738141 | -0.0087536 | 0.00159887 | 4.38E-08 | 0.254 | G | A |
| rs476490 | 0.02151 | 0.00363221 | 3.18E-09 | 0.0374 | G | A |
| rs477031 | -0.0359899 | 0.00270051 | 1.61E-40 | 0.0784 | C | T |
| rs4771674 | 0.0140486 | 0.0014485 | 3.05E-22 | 0.381 | A | G |
| rs477418 | 0.0253606 | 0.00383344 | 3.70E-11 | 0.0335 | A | C |
| rs4776342 | -0.0092998 | 0.00170413 | 4.84E-08 | 0.21 | G | A |
| rs4801771 | 0.021634 | 0.00225721 | 9.30E-22 | 0.111 | C | T |
| rs4802208 | 0.0142978 | 0.00190474 | 6.08E-14 | 0.16 | G | C |
| rs4802511 | -0.0161245 | 0.00179991 | 3.29E-19 | 0.2 | A | G |
| rs4803525 | -0.0092695 | 0.00141303 | 5.38E-11 | 0.419 | A | G |
| rs4803771 | -0.0337859 | 0.00605266 | 2.38E-08 | 0.0156 | G | C |
| rs4803791 | 0.0469245 | 0.00178681 | 5.27E-152 | 0.223 | A | G |
| rs4803818 | -0.0291478 | 0.00453397 | 1.29E-10 | 0.0254 | T | C |
| rs4803838 | -0.0450149 | 0.00530533 | 2.16E-17 | 0.0198 | T | C |
| rs4804149 | -0.0240719 | 0.0016234 | 9.65E-50 | 0.288 | C | T |
| rs4804160 | 0.0189365 | 0.00149687 | 1.11E-36 | 0.311 | A | T |
| rs4804510 | 0.0334243 | 0.00149883 | 3.67E-110 | 0.382 | A | G |
| rs4806498 | -0.0082873 | 0.00142758 | 6.43E-09 | 0.423 | T | C |
| rs4807570 | 0.011545 | 0.00171066 | 1.49E-11 | 0.21 | A | G |
| rs4808176 | -0.0110397 | 0.00195602 | 1.66E-08 | 0.158 | A | G |
| rs4808766 | -0.0142898 | 0.001587 | 2.17E-19 | 0.258 | C | G |
| rs4808884 | 0.0171341 | 0.00275893 | 5.28E-10 | 0.194 | A | G |
| rs4809412 | 0.0110992 | 0.00186905 | 2.88E-09 | 0.207 | C | A |
| rs4810296 | 0.0294726 | 0.0014442 | 1.43E-92 | 0.373 | T | C |
| rs4812445 | 0.0191936 | 0.00164256 | 1.52E-31 | 0.275 | G | A |
| rs4814666 | -0.0162053 | 0.00148374 | 9.06E-28 | 0.319 | A | G |
| rs4814674 | 0.0112522 | 0.00201815 | 2.47E-08 | 0.148 | A | G |
| rs4821108 | 0.0131355 | 0.00174933 | 5.96E-14 | 0.2 | C | G |
| rs4848460 | -0.0168897 | 0.00278902 | 1.40E-09 | 0.0742 | T | C |
| rs485742 | -0.008608 | 0.00151265 | 1.27E-08 | 0.309 | C | T |
| rs486416 | 0.0141526 | 0.00153118 | 2.40E-20 | 0.343 | G | A |
| rs4870941 | -0.04328 | 0.00164778 | 4.74E-152 | 0.232 | C | G |
| rs4920266 | 0.0220703 | 0.00391318 | 1.70E-08 | 0.0355 | A | G |
| rs4938309 | 0.0227289 | 0.00203846 | 7.16E-29 | 0.134 | T | C |
| rs4941641 | -0.0144306 | 0.00248607 | 6.45E-09 | 0.0847 | A | G |
| rs4946713 | 0.0112055 | 0.00139973 | 1.19E-15 | 0.447 | A | C |
| rs495265 | -0.009116 | 0.00142949 | 1.80E-10 | 0.396 | G | A |
| rs4963279 | 0.0123558 | 0.00222077 | 2.64E-08 | 0.114 | A | G |
| rs49675 | -0.0131875 | 0.00241881 | 4.98E-08 | 0.0912 | A | G |
| rs4970743 | -0.0111965 | 0.00143533 | 6.16E-15 | 0.37 | G | C |
| rs4970780 | -0.0123455 | 0.00221977 | 2.67E-08 | 0.414 | A | T |
| rs4970824 | 0.089795 | 0.00275232 | 1.00E-200 | 0.0695 | A | G |
| rs4977536 | 0.0137504 | 0.00156123 | 1.28E-18 | 0.27 | A | G |
| rs4980928 | 0.0137275 | 0.00181152 | 3.51E-14 | 0.188 | A | G |
| rs4984247 | 0.0126092 | 0.00201745 | 4.10E-10 | 0.135 | T | C |
| rs5015801 | -0.0626664 | 0.00568674 | 3.07E-28 | 0.0179 | C | A |
| rs5026917 | 0.00885758 | 0.00146939 | 1.66E-09 | 0.386 | C | A |
| rs515917 | -0.0106657 | 0.00152874 | 3.02E-12 | 0.3 | T | C |
| rs523118 | 0.0144134 | 0.00177081 | 3.97E-16 | 0.211 | T | G |
| rs523549 | 0.0105549 | 0.00147112 | 7.24E-13 | 0.41 | A | G |
| rs523736 | -0.0082275 | 0.00141226 | 5.68E-09 | 0.44 | G | A |
| rs530804537 | 0.255533 | 0.00718267 | 1.00E-200 | 0.0121 | A | G |
| rs533239 | -0.0107083 | 0.00172521 | 5.40E-10 | 0.22 | A | G |
| rs533452 | 0.0132522 | 0.00146424 | 1.42E-19 | 0.351 | A | G |
| rs535015446 | -0.0417126 | 0.00557633 | 7.42E-14 | 0.0179 | A | G |
| rs538857577 | 0.082361 | 0.0129445 | 1.98E-10 | 0.00351 | T | C |
| rs542387475 | -0.0870904 | 0.00756087 | 1.06E-30 | 0.0105 | C | T |
| rs546248239 | -0.0603691 | 0.00834518 | 4.69E-13 | 0.00829 | T | C |
| rs549752 | -0.0092828 | 0.00162236 | 1.05E-08 | 0.275 | G | A |
| rs553427 | 0.0403134 | 0.00140727 | 1.77E-180 | 0.473 | C | T |
| rs55638451 | -0.0210426 | 0.00341022 | 6.81E-10 | 0.0454 | T | C |
| rs55680582 | 0.0166411 | 0.0027274 | 1.05E-09 | 0.0708 | T | C |
| rs55696093 | -0.0382032 | 0.0017054 | 3.82E-111 | 0.211 | G | A |
| rs55702379 | -0.0191366 | 0.00150007 | 2.84E-37 | 0.328 | A | T |
| rs55714927 | 0.0349154 | 0.00183132 | 4.87E-81 | 0.189 | T | C |
| rs55726838 | -0.0784114 | 0.00751896 | 1.84E-25 | 0.00978 | A | G |
| rs55738493 | 0.0090706 | 0.0015043 | 1.64E-09 | 0.341 | A | G |
| rs55817205 | -0.0676289 | 0.00712427 | 2.25E-21 | 0.0116 | A | G |
| rs55874167 | 0.0130651 | 0.00228661 | 1.11E-08 | 0.103 | G | T |
| rs55890028 | -0.0078398 | 0.00143779 | 4.96E-08 | 0.416 | C | T |
| rs55921103 | 0.0126185 | 0.00147847 | 1.40E-17 | 0.359 | G | T |
| rs55924588 | -0.0176556 | 0.00318062 | 2.84E-08 | 0.0514 | C | T |
| rs55943924 | 0.0119692 | 0.00214672 | 2.47E-08 | 0.119 | A | G |
| rs55987642 | 0.0249028 | 0.00314124 | 2.23E-15 | 0.0521 | T | C |
| rs55989964 | -0.0270798 | 0.00354145 | 2.06E-14 | 0.0409 | G | C |
| rs56016492 | -0.0194897 | 0.00236186 | 1.56E-16 | 0.104 | T | C |
| rs56079255 | 0.0622631 | 0.00324875 | 7.22E-82 | 0.0497 | T | C |
| rs56109496 | -0.0210618 | 0.00314824 | 2.23E-11 | 0.0532 | A | G |
| rs56113850 | 0.0142152 | 0.00149255 | 1.66E-21 | 0.433 | T | C |
| rs56118251 | -0.0136844 | 0.00188919 | 4.37E-13 | 0.157 | G | A |
| rs56120442 | 0.0288155 | 0.00464342 | 5.45E-10 | 0.0261 | C | G |
| rs56174340 | -0.0161374 | 0.00141522 | 4.05E-30 | 0.407 | G | T |
| rs56208677 | 0.0241922 | 0.00278734 | 3.98E-18 | 0.0677 | T | C |
| rs56223081 | -0.0187347 | 0.00330254 | 1.40E-08 | 0.0492 | T | G |
| rs56225452 | -0.0171791 | 0.00180594 | 1.86E-21 | 0.18 | T | C |
| rs562260 | 0.0157117 | 0.00142734 | 3.51E-28 | 0.397 | G | C |
| rs56237989 | 0.0608271 | 0.00141635 | 1.00E-200 | 0.493 | T | G |
| rs56266464 | 0.113748 | 0.00290807 | 1.00E-200 | 0.0615 | A | G |
| rs56294298 | 0.0183291 | 0.00261275 | 2.30E-12 | 0.0847 | A | G |
| rs56299595 | -0.0182263 | 0.00211541 | 6.94E-18 | 0.125 | G | A |
| rs56305490 | 0.0140597 | 0.0024267 | 6.88E-09 | 0.0951 | A | G |
| rs56315738 | 0.111739 | 0.00888508 | 2.86E-36 | 0.00774 | T | C |
| rs56324302 | 0.0412216 | 0.00490281 | 4.18E-17 | 0.0249 | G | C |
| rs56336338 | 0.0192092 | 0.00205763 | 1.00E-20 | 0.134 | A | G |
| rs56344867 | 0.0116762 | 0.00151288 | 1.18E-14 | 0.331 | C | T |
| rs56361727 | 0.00820016 | 0.0014417 | 1.29E-08 | 0.376 | T | G |
| rs56383544 | 0.0133047 | 0.0024218 | 3.94E-08 | 0.104 | C | T |
| rs56393751 | 0.0375878 | 0.00560184 | 1.95E-11 | 0.0194 | T | C |
| rs56402945 | 0.023251 | 0.00324588 | 7.88E-13 | 0.0496 | A | G |
| rs564040572 | 0.066032 | 0.00574705 | 1.49E-30 | 0.0234 | T | C |
| rs564449 | -0.0267236 | 0.00218062 | 1.58E-34 | 0.115 | T | G |
| rs56881390 | 0.0169558 | 0.0024922 | 1.02E-11 | 0.0912 | C | T |
| rs571497 | 0.0145349 | 0.00194662 | 8.22E-14 | 0.153 | A | G |
| rs5746498 | -0.0133001 | 0.00169736 | 4.66E-15 | 0.238 | C | T |
| rs5752963 | -0.0267521 | 0.00365516 | 2.50E-13 | 0.0365 | A | G |
| rs5755688 | -0.0136768 | 0.00145715 | 6.23E-21 | 0.359 | A | G |
| rs5756079 | 0.00901334 | 0.00153205 | 4.02E-09 | 0.323 | G | C |
| rs582037 | -0.0221461 | 0.00156353 | 1.53E-45 | 0.309 | T | C |
| rs58298631 | -0.0360908 | 0.0014507 | 1.28E-136 | 0.381 | G | T |
| rs58421314 | 0.00983096 | 0.00171167 | 9.27E-09 | 0.265 | C | A |
| rs58478831 | -0.0142453 | 0.00151046 | 4.06E-21 | 0.351 | C | G |
| rs58630472 | 0.021718 | 0.00170691 | 4.37E-37 | 0.21 | A | G |
| rs58778188 | 0.012416 | 0.00207154 | 2.05E-09 | 0.166 | A | G |
| rs59264409 | 0.0183708 | 0.00225191 | 3.41E-16 | 0.111 | A | G |
| rs5930 | 0.0444469 | 0.00143298 | 1.00E-200 | 0.396 | A | G |
| rs59408219 | 0.0223639 | 0.00263422 | 2.07E-17 | 0.0762 | C | T |
| rs59488041 | -0.0116826 | 0.00199809 | 5.01E-09 | 0.139 | A | T |
| rs59669045 | 0.00950182 | 0.00159817 | 2.76E-09 | 0.253 | A | G |
| rs596901 | 0.0134122 | 0.0017465 | 1.60E-14 | 0.316 | C | T |
| rs598253 | 0.0421113 | 0.00149784 | 6.48E-174 | 0.338 | C | T |
| rs60006507 | 0.0102701 | 0.00169554 | 1.39E-09 | 0.219 | A | G |
| rs60049679 | -0.165573 | 0.00289549 | 1.00E-200 | 0.0731 | C | G |
| rs600711 | -0.0208297 | 0.00246383 | 2.81E-17 | 0.0871 | G | A |
| rs6008798 | -0.0086431 | 0.00157143 | 3.79E-08 | 0.279 | C | T |
| rs6010040 | -0.0110643 | 0.00168141 | 4.69E-11 | 0.22 | G | T |
| rs6016348 | 0.0164771 | 0.00203174 | 5.07E-16 | 0.135 | T | C |
| rs6016398 | -0.0162745 | 0.00177743 | 5.38E-20 | 0.204 | G | A |
| rs60191209 | 0.0193218 | 0.00180759 | 1.14E-26 | 0.181 | C | T |
| rs6022851 | -0.0115658 | 0.0014229 | 4.35E-16 | 0.446 | T | C |
| rs60239918 | -0.0264381 | 0.00395618 | 2.35E-11 | 0.035 | T | C |
| rs6029125 | 0.0221937 | 0.0023385 | 2.30E-21 | 0.155 | C | T |
| rs6029127 | 0.0122755 | 0.00158736 | 1.05E-14 | 0.26 | T | C |
| rs6029598 | 0.0191016 | 0.00259749 | 1.93E-13 | 0.0882 | T | C |
| rs603424 | -0.0158621 | 0.0018835 | 3.71E-17 | 0.169 | A | G |
| rs603624 | -0.01617 | 0.00227141 | 1.09E-12 | 0.134 | G | C |
| rs6044931 | 0.0162023 | 0.0018173 | 4.85E-19 | 0.185 | C | T |
| rs60484807 | 0.022374 | 0.00150193 | 3.46E-50 | 0.314 | G | A |
| rs604924 | 0.00933077 | 0.00147199 | 2.31E-10 | 0.336 | T | C |
| rs6051908 | 0.00926339 | 0.00150771 | 8.05E-10 | 0.305 | T | G |
| rs6053485 | 0.0156741 | 0.00173279 | 1.49E-19 | 0.208 | C | T |
| rs6060499 | -0.0088893 | 0.00162122 | 4.18E-08 | 0.386 | T | C |
| rs6062618 | -0.0109302 | 0.00168634 | 9.07E-11 | 0.267 | G | T |
| rs6063965 | -0.0134684 | 0.00218772 | 7.44E-10 | 0.111 | A | G |
| rs6072106 | -0.0162957 | 0.00215277 | 3.74E-14 | 0.125 | C | T |
| rs6072152 | -0.0114998 | 0.00201629 | 1.17E-08 | 0.152 | C | T |
| rs6072199 | -0.0111147 | 0.00161098 | 5.22E-12 | 0.254 | G | A |
| rs6072279 | -0.030099 | 0.00138821 | 3.04E-104 | 0.473 | A | G |
| rs6080840 | -0.0135792 | 0.0014691 | 2.39E-20 | 0.362 | C | G |
| rs6080872 | -0.0384792 | 0.00301519 | 2.68E-37 | 0.0574 | G | C |
| rs6090040 | -0.0143197 | 0.00147233 | 2.34E-22 | 0.483 | A | C |
| rs60988380 | -0.0130636 | 0.00230171 | 1.38E-08 | 0.103 | T | C |
| rs6102233 | -0.0093808 | 0.00149501 | 3.50E-10 | 0.41 | T | C |
| rs610249 | 0.0198505 | 0.00141994 | 2.07E-44 | 0.429 | G | A |
| rs61067468 | -0.0161236 | 0.00175685 | 4.41E-20 | 0.199 | A | G |
| rs61095569 | 0.0222568 | 0.00349439 | 1.90E-10 | 0.0414 | A | G |
| rs6129653 | -0.02428 | 0.00156488 | 2.72E-54 | 0.284 | T | C |
| rs6129859 | -0.0233168 | 0.00229357 | 2.81E-24 | 0.108 | A | G |
| rs614754 | -0.0574557 | 0.00637069 | 1.90E-19 | 0.0147 | C | G |
| rs61496306 | -0.0216025 | 0.00346387 | 4.47E-10 | 0.0448 | G | A |
| rs61586576 | 0.00976042 | 0.00167534 | 5.68E-09 | 0.219 | A | G |
| rs61642202 | -0.249672 | 0.00714805 | 1.00E-200 | 0.01 | C | A |
| rs61745773 | 0.0794384 | 0.00668341 | 1.40E-32 | 0.0127 | T | C |
| rs61754230 | -0.0491427 | 0.00574339 | 1.16E-17 | 0.0158 | T | C |
| rs61761209 | -0.0448956 | 0.00347822 | 4.08E-38 | 0.0506 | T | C |
| rs61775912 | 0.0397872 | 0.00616914 | 1.12E-10 | 0.015 | G | A |
| rs61797155 | 0.036303 | 0.00518784 | 2.60E-12 | 0.0196 | T | C |
| rs61824216 | -0.0098384 | 0.00178134 | 3.33E-08 | 0.187 | T | A |
| rs61836251 | -0.016605 | 0.00215505 | 1.31E-14 | 0.129 | G | T |
| rs61871243 | -0.0117123 | 0.0021431 | 4.63E-08 | 0.118 | A | G |
| rs61876729 | 0.0156721 | 0.00248946 | 3.07E-10 | 0.0881 | G | A |
| rs61882680 | 0.0279637 | 0.00410924 | 1.01E-11 | 0.0304 | T | C |
| rs61886346 | 0.0192465 | 0.00291427 | 4.00E-11 | 0.0612 | T | C |
| rs61897792 | -0.0170255 | 0.00241362 | 1.74E-12 | 0.1 | T | C |
| rs61932567 | 0.0160539 | 0.00267792 | 2.04E-09 | 0.079 | T | C |
| rs62023490 | 0.0145004 | 0.0022522 | 1.21E-10 | 0.105 | A | G |
| rs62048010 | -0.0238146 | 0.00307208 | 9.05E-15 | 0.0598 | C | T |
| rs62058275 | -0.0138641 | 0.00183259 | 3.87E-14 | 0.178 | T | C |
| rs62064334 | -0.0108522 | 0.00188884 | 9.17E-09 | 0.162 | G | C |
| rs62070652 | -0.0134136 | 0.00157535 | 1.67E-17 | 0.266 | T | C |
| rs62076103 | 0.0231653 | 0.00283678 | 3.19E-16 | 0.0651 | G | A |
| rs62076528 | -0.0079875 | 0.00142685 | 2.17E-08 | 0.403 | G | A |
| rs62078383 | -0.0097467 | 0.00145012 | 1.80E-11 | 0.409 | C | A |
| rs62082719 | -0.0415867 | 0.00227153 | 7.17E-75 | 0.109 | A | G |
| rs62104359 | 0.01818 | 0.00330309 | 3.71E-08 | 0.0472 | C | A |
| rs62118464 | -0.0289162 | 0.00256195 | 1.52E-29 | 0.1 | A | G |
| rs62118471 | 0.157338 | 0.00466912 | 1.00E-200 | 0.0266 | C | T |
| rs62119261 | 0.112605 | 0.00353352 | 1.00E-200 | 0.0414 | C | A |
| rs62120565 | 0.0811502 | 0.00262007 | 1.00E-200 | 0.0798 | C | A |
| rs62123977 | 0.0399403 | 0.00375884 | 2.26E-26 | 0.0347 | T | C |
| rs62129144 | -0.0326472 | 0.00585394 | 2.45E-08 | 0.0181 | A | G |
| rs62131083 | 0.0336714 | 0.00562635 | 2.17E-09 | 0.0183 | G | A |
| rs62131898 | -0.017987 | 0.00214858 | 5.69E-17 | 0.142 | G | A |
| rs62132778 | 0.0189496 | 0.0023264 | 3.78E-16 | 0.104 | C | G |
| rs62132800 | 0.0154216 | 0.00256377 | 1.80E-09 | 0.0866 | T | C |
| rs62134595 | 0.0343996 | 0.00606621 | 1.42E-08 | 0.0155 | C | T |
| rs62135111 | -0.0222474 | 0.00197865 | 2.49E-29 | 0.143 | A | G |
| rs62135543 | -0.0108601 | 0.00175586 | 6.21E-10 | 0.22 | G | C |
| rs62138973 | -0.0203921 | 0.00244755 | 7.97E-17 | 0.0933 | G | C |
| rs62211729 | 0.0311441 | 0.00476126 | 6.10E-11 | 0.0242 | A | G |
| rs62212773 | -0.0191917 | 0.00236441 | 4.78E-16 | 0.113 | A | C |
| rs62213195 | 0.0126781 | 0.00202926 | 4.17E-10 | 0.137 | A | C |
| rs62250794 | 0.0332957 | 0.00609649 | 4.72E-08 | 0.0176 | A | G |
| rs62255644 | -0.008405 | 0.00153292 | 4.18E-08 | 0.295 | A | G |
| rs62264113 | -0.0144742 | 0.00224904 | 1.23E-10 | 0.11 | A | G |
| rs62317502 | 0.0114809 | 0.00197297 | 5.92E-09 | 0.239 | G | A |
| rs62363367 | 0.0311531 | 0.00500228 | 4.73E-10 | 0.0204 | C | A |
| rs62390763 | -0.0109192 | 0.00196896 | 2.93E-08 | 0.144 | T | C |
| rs62394865 | -0.0183529 | 0.00314236 | 5.20E-09 | 0.0569 | A | T |
| rs62405964 | -0.0446562 | 0.00662987 | 1.63E-11 | 0.0132 | C | T |
| rs62419249 | -0.0121051 | 0.00140411 | 6.63E-18 | 0.478 | A | G |
| rs62440900 | 0.0369227 | 0.00215402 | 7.30E-66 | 0.119 | C | T |
| rs62440924 | 0.0125645 | 0.00188839 | 2.86E-11 | 0.171 | G | A |
| rs62447924 | 0.0207946 | 0.00290384 | 8.01E-13 | 0.0632 | C | T |
| rs62496173 | -0.0115131 | 0.00139528 | 1.56E-16 | 0.499 | G | T |
| rs62509311 | 0.0152619 | 0.00156162 | 1.47E-22 | 0.279 | T | A |
| rs62521040 | -0.0231841 | 0.00300752 | 1.27E-14 | 0.0571 | T | C |
| rs62521592 | 0.0274217 | 0.00352869 | 7.78E-15 | 0.0483 | T | C |
| rs62523860 | 0.0394738 | 0.00577439 | 8.14E-12 | 0.0176 | A | G |
| rs62621812 | -0.0302076 | 0.00459016 | 4.67E-11 | 0.0236 | A | G |
| rs634177 | 0.0689413 | 0.00610262 | 1.36E-29 | 0.0141 | G | A |
| rs638769 | 0.0084347 | 0.0015297 | 3.51E-08 | 0.293 | G | C |
| rs640306 | -0.0100429 | 0.00147699 | 1.05E-11 | 0.344 | T | C |
| rs6422312 | 0.00844403 | 0.00142034 | 2.76E-09 | 0.436 | G | T |
| rs6427128 | 0.0116419 | 0.00207066 | 1.88E-08 | 0.128 | A | C |
| rs642803 | -0.0101855 | 0.00140363 | 3.97E-13 | 0.456 | T | C |
| rs6431630 | -0.0241194 | 0.00225665 | 1.16E-26 | 0.105 | A | G |
| rs6441313 | -0.0125638 | 0.00141675 | 7.44E-19 | 0.463 | A | G |
| rs644492 | -0.0135814 | 0.00178557 | 2.82E-14 | 0.188 | G | A |
| rs6448432 | -0.0087429 | 0.001515 | 7.89E-09 | 0.296 | A | G |
| rs6453188 | -0.0144711 | 0.00160664 | 2.12E-19 | 0.266 | G | A |
| rs6455682 | 0.0276241 | 0.0025151 | 4.60E-28 | 0.0905 | A | G |
| rs6482072 | 0.00810361 | 0.00142876 | 1.41E-08 | 0.386 | G | A |
| rs648673 | 0.039514 | 0.00208662 | 5.67E-80 | 0.131 | C | G |
| rs6499863 | -0.0139858 | 0.0018861 | 1.21E-13 | 0.16 | A | G |
| rs6509222 | -0.0094227 | 0.0014188 | 3.11E-11 | 0.446 | T | C |
| rs651164 | 0.00980974 | 0.00153409 | 1.61E-10 | 0.305 | A | G |
| rs6511701 | 0.015696 | 0.00180862 | 4.01E-18 | 0.209 | A | C |
| rs653152 | -0.0087034 | 0.00148573 | 4.69E-09 | 0.331 | G | A |
| rs6537837 | -0.0154092 | 0.00179389 | 8.71E-18 | 0.179 | T | C |
| rs6545946 | 0.0093186 | 0.0016635 | 2.12E-08 | 0.224 | T | C |
| rs6547829 | -0.0176223 | 0.00245237 | 6.68E-13 | 0.0907 | T | C |
| rs655744 | -0.0082253 | 0.00140449 | 4.73E-09 | 0.456 | G | T |
| rs6557915 | 0.0086521 | 0.00154641 | 2.21E-08 | 0.283 | C | A |
| rs657801 | -0.0090541 | 0.00150713 | 1.88E-09 | 0.315 | C | T |
| rs6589706 | 0.00774224 | 0.00141649 | 4.61E-08 | 0.478 | A | G |
| rs6591182 | 0.00812547 | 0.00139554 | 5.80E-09 | 0.491 | G | T |
| rs6597615 | 0.0193795 | 0.00182187 | 2.00E-26 | 0.176 | C | T |
| rs6598858 | -0.0087274 | 0.00146292 | 2.44E-09 | 0.459 | T | C |
| rs660247 | 0.0101861 | 0.00139865 | 3.27E-13 | 0.469 | A | G |
| rs6602909 | -0.0211903 | 0.00151305 | 1.45E-44 | 0.33 | C | T |
| rs662138 | -0.0410048 | 0.00181103 | 1.68E-113 | 0.181 | G | C |
| rs66466742 | 0.0513864 | 0.00354454 | 1.26E-47 | 0.0388 | T | C |
| rs6660791 | 0.00953143 | 0.00158605 | 1.86E-09 | 0.264 | G | A |
| rs6671847 | 0.00756068 | 0.00138624 | 4.92E-08 | 0.493 | A | G |
| rs6674941 | 0.00874165 | 0.00148673 | 4.11E-09 | 0.363 | T | A |
| rs6682862 | 0.0137009 | 0.00188331 | 3.47E-13 | 0.164 | A | G |
| rs66836394 | -0.0416468 | 0.0067055 | 5.27E-10 | 0.0126 | G | C |
| rs6684364 | 0.0095788 | 0.00161878 | 3.27E-09 | 0.247 | A | G |
| rs66879753 | -0.0108172 | 0.00193277 | 2.18E-08 | 0.152 | A | C |
| rs6689 | -0.0389564 | 0.0018946 | 6.03E-94 | 0.203 | G | A |
| rs66952057 | -0.0094336 | 0.00164844 | 1.05E-08 | 0.325 | T | C |
| rs6707559 | -0.0082731 | 0.00142406 | 6.27E-09 | 0.402 | C | T |
| rs6709864 | -0.0159732 | 0.00284178 | 1.90E-08 | 0.0633 | G | C |
| rs6714780 | -0.0200812 | 0.00332071 | 1.47E-09 | 0.052 | A | G |
| rs6726046 | 0.00847486 | 0.00153241 | 3.19E-08 | 0.292 | A | G |
| rs67269656 | 0.0103803 | 0.00157377 | 4.23E-11 | 0.265 | T | C |
| rs6734238 | 0.00932154 | 0.00140841 | 3.63E-11 | 0.407 | G | A |
| rs6739502 | 0.0278015 | 0.00138044 | 3.32E-90 | 0.491 | G | A |
| rs6747243 | 0.00874299 | 0.00139781 | 3.98E-10 | 0.434 | C | T |
| rs67594359 | -0.0120338 | 0.00200935 | 2.11E-09 | 0.165 | A | G |
| rs6764634 | 0.0195552 | 0.00340631 | 9.42E-09 | 0.0434 | C | T |
| rs6768823 | -0.0106588 | 0.00147352 | 4.70E-13 | 0.345 | A | G |
| rs67734975 | -0.0524637 | 0.00330436 | 9.13E-57 | 0.0451 | G | C |
| rs67740167 | 0.0167461 | 0.00152909 | 6.52E-28 | 0.292 | C | G |
| rs677929 | 0.0193154 | 0.00280495 | 5.73E-12 | 0.0697 | A | G |
| rs6785233 | -0.0145473 | 0.00258571 | 1.84E-08 | 0.0785 | G | T |
| rs67890964 | 0.0182605 | 0.0014752 | 3.42E-35 | 0.381 | C | T |
| rs67896106 | -0.0114338 | 0.00202879 | 1.74E-08 | 0.133 | A | C |
| rs6792725 | -0.015304 | 0.00156248 | 1.19E-22 | 0.319 | A | G |
| rs6794370 | -0.0108697 | 0.00186807 | 5.93E-09 | 0.205 | A | C |
| rs679582 | -0.009832 | 0.0014262 | 5.43E-12 | 0.381 | G | A |
| rs68013139 | 0.0108536 | 0.00164348 | 4.00E-11 | 0.232 | C | T |
| rs6806351 | 0.0103696 | 0.0017322 | 2.15E-09 | 0.219 | T | C |
| rs681343 | -0.0292543 | 0.00139286 | 6.15E-98 | 0.494 | T | C |
| rs68137036 | 0.00948941 | 0.00154094 | 7.36E-10 | 0.282 | G | A |
| rs683800 | 0.0136811 | 0.00140267 | 1.78E-22 | 0.433 | C | T |
| rs6850707 | -0.011756 | 0.00159916 | 1.96E-13 | 0.262 | A | G |
| rs6861546 | -0.0169496 | 0.00159647 | 2.49E-26 | 0.253 | T | C |
| rs6869845 | 0.0167463 | 0.00139999 | 5.64E-33 | 0.451 | C | T |
| rs6872094 | -0.0151427 | 0.00267401 | 1.49E-08 | 0.0727 | C | A |
| rs6874435 | -0.0179406 | 0.00213293 | 4.06E-17 | 0.125 | G | A |
| rs687914 | -0.0093628 | 0.0016606 | 1.72E-08 | 0.242 | T | G |
| rs6889859 | -0.0100618 | 0.00183038 | 3.86E-08 | 0.175 | G | C |
| rs6893262 | -0.0137506 | 0.00235044 | 4.91E-09 | 0.0991 | G | A |
| rs6905073 | 0.0277466 | 0.0014583 | 1.03E-80 | 0.351 | T | G |
| rs6924805 | -0.0086737 | 0.00141808 | 9.56E-10 | 0.408 | G | T |
| rs6930797 | 0.0148768 | 0.00220014 | 1.36E-11 | 0.112 | T | C |
| rs6931044 | -0.015808 | 0.0015587 | 3.60E-24 | 0.426 | T | G |
| rs6932167 | 0.0240472 | 0.00317253 | 3.46E-14 | 0.108 | A | G |
| rs6935921 | 0.0343237 | 0.00153954 | 4.15E-110 | 0.302 | C | T |
| rs6939741 | -0.0175516 | 0.00140953 | 1.36E-35 | 0.46 | G | A |
| rs6940342 | 0.00925897 | 0.00143165 | 9.97E-11 | 0.375 | T | C |
| rs6960950 | 0.0128142 | 0.00200027 | 1.49E-10 | 0.165 | G | T |
| rs6967728 | 0.0179411 | 0.00183651 | 1.53E-22 | 0.183 | A | G |
| rs6992869 | -0.0102426 | 0.00143561 | 9.70E-13 | 0.375 | C | T |
| rs6993155 | 0.0419033 | 0.00411326 | 2.26E-24 | 0.0315 | G | A |
| rs6999569 | 0.0537463 | 0.00140371 | 1.00E-200 | 0.472 | G | A |
| rs701078 | 0.0122116 | 0.00212486 | 9.08E-09 | 0.126 | T | C |
| rs7012814 | -0.0323145 | 0.00143179 | 8.68E-113 | 0.472 | A | G |
| rs7017840 | 0.0103219 | 0.00171532 | 1.77E-09 | 0.206 | G | T |
| rs7025486 | -0.009415 | 0.00157012 | 2.02E-09 | 0.263 | A | G |
| rs704 | -0.018865 | 0.00138898 | 5.13E-42 | 0.477 | A | G |
| rs7070793 | 0.00834316 | 0.00142073 | 4.29E-09 | 0.395 | A | G |
| rs7081482 | -0.0083167 | 0.0014154 | 4.21E-09 | 0.409 | G | T |
| rs708686 | -0.0110129 | 0.00155525 | 1.43E-12 | 0.278 | T | C |
| rs7124487 | 0.011576 | 0.00182058 | 2.04E-10 | 0.186 | T | C |
| rs71311871 | 0.0379602 | 0.00252076 | 3.01E-51 | 0.0814 | G | A |
| rs7133378 | 0.0102106 | 0.00149206 | 7.74E-12 | 0.32 | A | G |
| rs71352239 | -0.0735781 | 0.00166399 | 1.00E-200 | 0.315 | T | C |
| rs71367414 | 0.0130014 | 0.00184322 | 1.74E-12 | 0.204 | T | C |
| rs71420066 | -0.0114126 | 0.0018511 | 7.03E-10 | 0.178 | T | C |
| rs7142965 | -0.0138261 | 0.00140441 | 7.22E-23 | 0.475 | T | C |
| rs71435595 | -0.0290081 | 0.00374283 | 9.17E-15 | 0.0378 | C | A |
| rs71441185 | -0.0253949 | 0.00411003 | 6.46E-10 | 0.0305 | G | A |
| rs71480307 | -0.0170486 | 0.00270307 | 2.84E-10 | 0.0729 | A | G |
| rs714948 | -0.0399903 | 0.00230282 | 1.50E-67 | 0.111 | A | C |
| rs71526371 | 0.0221247 | 0.00303154 | 2.92E-13 | 0.0595 | G | T |
| rs7157399 | 0.0253853 | 0.00200506 | 9.77E-37 | 0.14 | T | C |
| rs71607360 | 0.0150016 | 0.00250119 | 2.00E-09 | 0.0863 | C | A |
| rs7203984 | -0.0269922 | 0.00176116 | 5.10E-53 | 0.193 | C | A |
| rs7214022 | 0.0123443 | 0.00160245 | 1.33E-14 | 0.254 | G | T |
| rs7214761 | -0.0130194 | 0.00164803 | 2.79E-15 | 0.237 | A | G |
| rs7216105 | 0.0277789 | 0.0024712 | 2.56E-29 | 0.0851 | T | C |
| rs721795 | 0.0363305 | 0.00508365 | 8.90E-13 | 0.0188 | T | C |
| rs7229377 | -0.0114288 | 0.0017546 | 7.34E-11 | 0.202 | T | C |
| rs7246666 | -0.0426558 | 0.0023439 | 5.29E-74 | 0.131 | A | G |
| rs7247937 | 0.0166358 | 0.00155829 | 1.32E-26 | 0.283 | C | G |
| rs7251161 | 0.0286581 | 0.00169183 | 2.32E-64 | 0.297 | A | G |
| rs7252216 | -0.0203405 | 0.00266547 | 2.33E-14 | 0.0738 | G | C |
| rs7255 | 0.0156884 | 0.00139496 | 2.41E-29 | 0.457 | T | C |
| rs7255130 | 0.00896571 | 0.00157407 | 1.23E-08 | 0.262 | C | T |
| rs7256743 | -0.020727 | 0.0027347 | 3.48E-14 | 0.0802 | C | G |
| rs7257072 | 0.0129518 | 0.00138472 | 8.49E-21 | 0.488 | T | C |
| rs7258215 | -0.009217 | 0.00156315 | 3.71E-09 | 0.401 | C | T |
| rs7259930 | 0.0102134 | 0.00187166 | 4.85E-08 | 0.188 | A | G |
| rs7260450 | -0.0105932 | 0.00147364 | 6.55E-13 | 0.38 | T | G |
| rs7260871 | 0.0277173 | 0.00394486 | 2.12E-12 | 0.0347 | G | A |
| rs72609847 | -0.0165914 | 0.00270954 | 9.16E-10 | 0.0798 | T | C |
| rs72616903 | -0.0205511 | 0.0018913 | 1.67E-27 | 0.164 | C | T |
| rs7261820 | 0.0304061 | 0.00201064 | 1.15E-51 | 0.137 | A | G |
| rs72647321 | -0.0300863 | 0.00514657 | 5.04E-09 | 0.0211 | T | C |
| rs72647336 | -0.0344097 | 0.00344185 | 1.56E-23 | 0.0492 | A | G |
| rs72647819 | -0.0322466 | 0.00491935 | 5.56E-11 | 0.0211 | T | C |
| rs72654445 | -0.0743777 | 0.00682861 | 1.26E-27 | 0.0121 | A | G |
| rs72654472 | 0.0622542 | 0.00470522 | 5.82E-40 | 0.0234 | T | G |
| rs72655677 | 0.0502999 | 0.00329399 | 1.21E-52 | 0.0486 | A | G |
| rs72660548 | -0.0704297 | 0.00495026 | 6.19E-46 | 0.0202 | G | C |
| rs72663045 | -0.0403007 | 0.00503784 | 1.25E-15 | 0.0196 | G | T |
| rs72675550 | -0.0317938 | 0.00537403 | 3.29E-09 | 0.0178 | A | G |
| rs72695404 | -0.0215017 | 0.0034841 | 6.77E-10 | 0.0458 | T | C |
| rs72695692 | 0.0269046 | 0.00487649 | 3.44E-08 | 0.0211 | A | C |
| rs72705225 | -0.0297979 | 0.0036085 | 1.49E-16 | 0.0415 | A | G |
| rs72749499 | -0.0287142 | 0.00278043 | 5.31E-25 | 0.0704 | G | A |
| rs72749998 | 0.0255776 | 0.00398674 | 1.40E-10 | 0.0343 | C | A |
| rs72752243 | 0.0189296 | 0.00339961 | 2.57E-08 | 0.049 | T | C |
| rs72766599 | 0.0248593 | 0.00319297 | 6.94E-15 | 0.0499 | G | A |
| rs72768359 | 0.0343939 | 0.00241114 | 3.64E-46 | 0.109 | G | T |
| rs72772042 | 0.0123084 | 0.00195634 | 3.14E-10 | 0.146 | A | C |
| rs72775410 | 0.0222218 | 0.00354926 | 3.83E-10 | 0.0462 | A | G |
| rs72780178 | -0.0608072 | 0.0043391 | 1.28E-44 | 0.027 | C | A |
| rs72782175 | 0.095524 | 0.00604369 | 2.85E-56 | 0.0144 | C | T |
| rs72783088 | 0.0199122 | 0.00357538 | 2.56E-08 | 0.0401 | T | C |
| rs72784396 | -0.0351719 | 0.00347025 | 3.85E-24 | 0.0445 | T | C |
| rs72786781 | 0.0271023 | 0.00462516 | 4.64E-09 | 0.0261 | A | T |
| rs72787084 | -0.0557625 | 0.00401637 | 7.94E-44 | 0.0319 | T | G |
| rs72791871 | -0.044733 | 0.00691957 | 1.01E-10 | 0.0129 | A | C |
| rs72796715 | -0.0353901 | 0.00597556 | 3.17E-09 | 0.0143 | A | T |
| rs72800939 | 0.0853859 | 0.00601014 | 8.29E-46 | 0.0149 | T | A |
| rs72804857 | -0.0147946 | 0.00198245 | 8.47E-14 | 0.14 | C | G |
| rs72807600 | 0.032236 | 0.00380936 | 2.62E-17 | 0.0367 | G | T |
| rs72808908 | 0.0167397 | 0.00210926 | 2.08E-15 | 0.139 | T | C |
| rs72823020 | 0.0149316 | 0.00212243 | 1.99E-12 | 0.123 | A | T |
| rs72826970 | -0.0197314 | 0.00347483 | 1.36E-08 | 0.0457 | G | A |
| rs72832324 | 0.0141764 | 0.00222434 | 1.85E-10 | 0.111 | G | A |
| rs72832352 | 0.033507 | 0.00442541 | 3.69E-14 | 0.0279 | T | A |
| rs72836561 | 0.0297626 | 0.0040428 | 1.81E-13 | 0.0304 | T | C |
| rs72842819 | 0.0146983 | 0.00225018 | 6.49E-11 | 0.111 | C | A |
| rs72846154 | 0.0487131 | 0.00655133 | 1.04E-13 | 0.0141 | T | C |
| rs72870502 | 0.0112453 | 0.0019122 | 4.08E-09 | 0.169 | T | C |
| rs72902579 | 0.0974863 | 0.0036918 | 1.16E-153 | 0.0379 | C | T |
| rs72926966 | 0.019722 | 0.00151532 | 1.00E-38 | 0.301 | A | C |
| rs72927451 | -0.028137 | 0.00371909 | 3.86E-14 | 0.0472 | T | C |
| rs72929768 | -0.0158022 | 0.0018034 | 1.91E-18 | 0.194 | T | C |
| rs72964564 | 0.0100897 | 0.00164414 | 8.42E-10 | 0.235 | C | A |
| rs72969958 | -0.0619782 | 0.00781519 | 2.18E-15 | 0.00892 | T | G |
| rs72974722 | -0.0211309 | 0.00184449 | 2.19E-30 | 0.177 | C | A |
| rs7300192 | -0.0134682 | 0.00146867 | 4.72E-20 | 0.365 | A | G |
| rs73007510 | 0.0491233 | 0.00579456 | 2.30E-17 | 0.0171 | T | C |
| rs73009557 | 0.0696026 | 0.00455244 | 9.04E-53 | 0.0243 | A | G |
| rs73013176 | 0.214147 | 0.00676303 | 1.00E-200 | 0.0118 | C | T |
| rs73015030 | 0.13344 | 0.00402798 | 1.00E-200 | 0.0318 | A | G |
| rs73015195 | 0.0485031 | 0.0066867 | 4.06E-13 | 0.0117 | A | G |
| rs73017418 | -0.0159877 | 0.00220666 | 4.32E-13 | 0.121 | G | A |
| rs73019211 | 0.0987683 | 0.00796575 | 2.64E-35 | 0.00815 | A | G |
| rs73019326 | -0.0203257 | 0.0025727 | 2.78E-15 | 0.0787 | G | T |
| rs73021677 | -0.0384518 | 0.00450507 | 1.40E-17 | 0.0249 | T | C |
| rs73025532 | 0.035174 | 0.00242417 | 1.05E-47 | 0.0935 | T | C |
| rs73027814 | 0.0138788 | 0.00226842 | 9.46E-10 | 0.117 | C | T |
| rs73034498 | -0.0306336 | 0.00443459 | 4.92E-12 | 0.0288 | T | C |
| rs73034885 | -0.0238804 | 0.00402511 | 2.98E-09 | 0.0336 | T | C |
| rs73036542 | 0.0187557 | 0.00180035 | 2.06E-25 | 0.205 | G | A |
| rs73048234 | 0.0252657 | 0.0032353 | 5.75E-15 | 0.0573 | A | G |
| rs73048351 | 0.254968 | 0.00740551 | 1.00E-200 | 0.00968 | A | C |
| rs73048605 | 0.0476603 | 0.00872236 | 4.65E-08 | 0.007 | T | C |
| rs73051677 | 0.0415236 | 0.00386107 | 5.65E-27 | 0.0364 | A | G |
| rs73075609 | -0.0479201 | 0.00475232 | 6.53E-24 | 0.0239 | T | C |
| rs7310350 | 0.00801946 | 0.00146036 | 3.99E-08 | 0.387 | T | C |
| rs73114543 | -0.0175189 | 0.00315039 | 2.68E-08 | 0.0552 | G | A |
| rs73121275 | 0.0179849 | 0.00317058 | 1.41E-08 | 0.0556 | T | C |
| rs73124945 | 0.0261148 | 0.00206033 | 8.13E-37 | 0.169 | C | G |
| rs73147887 | -0.0158654 | 0.00178832 | 7.21E-19 | 0.212 | G | C |
| rs7316678 | 0.00882428 | 0.00141086 | 3.99E-10 | 0.413 | C | T |
| rs7317982 | -0.0086852 | 0.0013902 | 4.17E-10 | 0.47 | T | C |
| rs73201772 | -0.0140542 | 0.0022076 | 1.94E-10 | 0.114 | T | C |
| rs73205552 | 0.00958517 | 0.00174797 | 4.17E-08 | 0.197 | G | A |
| rs73214164 | 0.0158347 | 0.00177944 | 5.65E-19 | 0.191 | C | T |
| rs73230015 | -0.0215529 | 0.00326338 | 3.99E-11 | 0.048 | A | G |
| rs73238159 | 0.0127723 | 0.00205673 | 5.30E-10 | 0.129 | T | C |
| rs73239113 | 0.014576 | 0.00211516 | 5.53E-12 | 0.129 | C | T |
| rs73267833 | 0.0162106 | 0.00180747 | 3.00E-19 | 0.198 | A | G |
| rs7327867 | -0.0198561 | 0.00139678 | 7.33E-46 | 0.474 | G | A |
| rs7330899 | -0.0143148 | 0.00161093 | 6.33E-19 | 0.244 | A | G |
| rs73429745 | -0.01062 | 0.00173618 | 9.54E-10 | 0.201 | T | C |
| rs7343130 | 0.0988824 | 0.0015664 | 1.00E-200 | 0.424 | A | G |
| rs73487492 | 0.0293821 | 0.00287776 | 1.79E-24 | 0.0621 | G | A |
| rs73526734 | 0.0344916 | 0.00596628 | 7.42E-09 | 0.0167 | A | G |
| rs73568038 | -0.0255869 | 0.00310678 | 1.78E-16 | 0.059 | C | G |
| rs73568948 | 0.0197468 | 0.00235693 | 5.37E-17 | 0.0965 | C | T |
| rs73664374 | -0.0273287 | 0.00263672 | 3.59E-25 | 0.0774 | G | C |
| rs7366900 | -0.0092772 | 0.00167421 | 3.00E-08 | 0.256 | C | T |
| rs73672461 | -0.0185311 | 0.00309821 | 2.21E-09 | 0.0545 | A | G |
| rs73898203 | -0.0196899 | 0.00300146 | 5.38E-11 | 0.0561 | C | T |
| rs73936968 | -0.0510401 | 0.00534584 | 1.33E-21 | 0.0188 | A | G |
| rs740006 | -0.0151526 | 0.00231314 | 5.73E-11 | 0.102 | C | T |
| rs74019283 | 0.0208027 | 0.00375364 | 2.99E-08 | 0.0385 | T | G |
| rs74035509 | -0.0232369 | 0.00275116 | 3.01E-17 | 0.0745 | T | C |
| rs7404072 | 0.0102936 | 0.00155156 | 3.26E-11 | 0.286 | C | T |
| rs7404577 | -0.0118478 | 0.00153385 | 1.13E-14 | 0.443 | C | T |
| rs740505 | -0.0108953 | 0.0019389 | 1.92E-08 | 0.154 | C | T |
| rs740516 | 0.0494536 | 0.00194807 | 3.61E-142 | 0.155 | G | C |
| rs74090765 | -0.0144654 | 0.00183467 | 3.16E-15 | 0.173 | G | T |
| rs7415034 | 0.0188309 | 0.00245136 | 1.57E-14 | 0.0933 | T | C |
| rs742067 | 0.00844064 | 0.00146611 | 8.55E-09 | 0.374 | G | A |
| rs74341202 | 0.0425579 | 0.00322656 | 1.00E-39 | 0.0497 | A | G |
| rs74398580 | 0.0313204 | 0.00490741 | 1.74E-10 | 0.0247 | T | C |
| rs74576624 | -0.036901 | 0.00614494 | 1.91E-09 | 0.0138 | A | T |
| rs74607435 | 0.0613283 | 0.00324129 | 7.67E-80 | 0.0494 | C | T |
| rs74629672 | -0.0461633 | 0.00367704 | 3.76E-36 | 0.0441 | A | T |
| rs74637757 | -0.0317 | 0.0026868 | 3.98E-32 | 0.0765 | A | G |
| rs74695562 | 0.0369214 | 0.00369832 | 1.80E-23 | 0.0417 | G | T |
| rs74717300 | -0.0608654 | 0.00322223 | 1.40E-79 | 0.0536 | T | G |
| rs74747585 | 0.109328 | 0.00507731 | 7.70E-103 | 0.0202 | C | T |
| rs74827760 | 0.0435997 | 0.00387904 | 2.60E-29 | 0.0349 | T | C |
| rs748336 | -0.0092432 | 0.00145509 | 2.12E-10 | 0.355 | T | C |
| rs74833757 | 0.0159845 | 0.00251799 | 2.18E-10 | 0.0842 | C | T |
| rs74852793 | 0.0222924 | 0.00325987 | 8.01E-12 | 0.0507 | G | A |
| rs74887247 | -0.0318732 | 0.0050761 | 3.41E-10 | 0.0195 | G | T |
| rs748897 | 0.00952218 | 0.00158792 | 2.01E-09 | 0.272 | G | C |
| rs74974644 | 0.0230797 | 0.00265225 | 3.26E-18 | 0.0789 | A | G |
| rs75014601 | -0.0494839 | 0.00842574 | 4.28E-09 | 0.00649 | A | G |
| rs7501528 | 0.0112358 | 0.00186249 | 1.61E-09 | 0.199 | G | C |
| rs75050571 | -0.0282488 | 0.00498383 | 1.44E-08 | 0.0236 | T | A |
| rs7512010 | 0.0182638 | 0.00179491 | 2.56E-24 | 0.193 | A | T |
| rs75120785 | -0.0183037 | 0.00243565 | 5.69E-14 | 0.0908 | G | T |
| rs75144964 | 0.0403005 | 0.00598945 | 1.71E-11 | 0.0163 | G | C |
| rs7514842 | 0.0121441 | 0.00156996 | 1.03E-14 | 0.301 | T | G |
| rs75164422 | 0.0405068 | 0.00406376 | 2.11E-23 | 0.0299 | A | G |
| rs75166914 | -0.0121731 | 0.00217754 | 2.27E-08 | 0.124 | A | T |
| rs75178253 | 0.03185 | 0.00268296 | 1.67E-32 | 0.0781 | G | A |
| rs75193300 | 0.0230849 | 0.00362675 | 1.95E-10 | 0.0374 | T | A |
| rs7519734 | 0.0111937 | 0.00167938 | 2.64E-11 | 0.223 | C | T |
| rs7520254 | 0.0176207 | 0.00244066 | 5.21E-13 | 0.0924 | C | A |
| rs75211012 | -0.0357711 | 0.00308546 | 4.45E-31 | 0.0626 | A | C |
| rs7525503 | -0.0712849 | 0.00498832 | 2.51E-46 | 0.0229 | T | G |
| rs75259112 | 0.0479585 | 0.00699706 | 7.18E-12 | 0.0113 | T | C |
| rs7527776 | 0.00950538 | 0.00144496 | 4.76E-11 | 0.383 | T | C |
| rs7528078 | 0.00975238 | 0.00140209 | 3.51E-12 | 0.484 | C | T |
| rs75307154 | 0.0262359 | 0.00253062 | 3.49E-25 | 0.0994 | T | C |
| rs7542853 | 0.0112332 | 0.00170258 | 4.17E-11 | 0.217 | T | C |
| rs75463276 | -0.0498676 | 0.00621041 | 9.77E-16 | 0.0161 | G | C |
| rs75482932 | 0.0448022 | 0.00751301 | 2.47E-09 | 0.0115 | C | T |
| rs75501158 | -0.0133959 | 0.002305 | 6.19E-09 | 0.111 | T | A |
| rs7550711 | 0.0348682 | 0.00406074 | 8.95E-18 | 0.0295 | T | C |
| rs7551124 | 0.0168091 | 0.002095 | 1.03E-15 | 0.126 | C | T |
| rs7556152 | 0.0162554 | 0.00281877 | 8.08E-09 | 0.0677 | G | T |
| rs7556983 | 0.015424 | 0.00228012 | 1.34E-11 | 0.104 | A | G |
| rs7557742 | 0.0120066 | 0.00206954 | 6.57E-09 | 0.138 | G | C |
| rs75588192 | -0.0164726 | 0.0021227 | 8.48E-15 | 0.14 | A | G |
| rs7560093 | 0.0201978 | 0.00330822 | 1.03E-09 | 0.0493 | A | C |
| rs7564456 | -0.0151491 | 0.00222104 | 9.06E-12 | 0.29 | A | T |
| rs75667995 | 0.0291431 | 0.00273128 | 1.40E-26 | 0.0682 | C | T |
| rs75679231 | -0.0525064 | 0.00451792 | 3.19E-31 | 0.0275 | C | A |
| rs75679663 | 0.0609391 | 0.00742965 | 2.36E-16 | 0.00881 | A | C |
| rs7569721 | 0.0123882 | 0.00179044 | 4.55E-12 | 0.19 | C | T |
| rs75734873 | 0.0111849 | 0.00200981 | 2.62E-08 | 0.141 | A | G |
| rs757360 | 0.00910028 | 0.00143751 | 2.44E-10 | 0.451 | G | A |
| rs7573769 | 0.0181467 | 0.00183119 | 3.77E-23 | 0.176 | G | A |
| rs75750038 | 0.0384784 | 0.00426798 | 1.96E-19 | 0.0307 | A | G |
| rs75786411 | 0.0283238 | 0.00277361 | 1.75E-24 | 0.0741 | A | G |
| rs75841075 | -0.031002 | 0.00462931 | 2.13E-11 | 0.0233 | A | G |
| rs75907879 | -0.0195729 | 0.0021867 | 3.53E-19 | 0.125 | T | C |
| rs75919952 | 0.0181845 | 0.0031774 | 1.05E-08 | 0.0515 | T | C |
| rs75925552 | 0.0194437 | 0.00298872 | 7.73E-11 | 0.0622 | G | C |
| rs7595144 | 0.0133433 | 0.00140534 | 2.21E-21 | 0.426 | A | G |
| rs75954052 | -0.0138748 | 0.00253733 | 4.54E-08 | 0.0829 | T | C |
| rs75980706 | 0.0359561 | 0.00528501 | 1.02E-11 | 0.0188 | T | G |
| rs75991907 | -0.0839107 | 0.00678228 | 3.70E-35 | 0.0119 | A | T |
| rs7601153 | 0.0131458 | 0.00142061 | 2.17E-20 | 0.387 | G | C |
| rs76041277 | -0.0465039 | 0.00437797 | 2.35E-26 | 0.028 | T | C |
| rs76064688 | -0.0134238 | 0.00207453 | 9.75E-11 | 0.129 | C | T |
| rs760719 | -0.0096611 | 0.00142207 | 1.09E-11 | 0.428 | T | C |
| rs7608713 | -0.013282 | 0.00160931 | 1.54E-16 | 0.248 | A | G |
| rs76143792 | -0.0258034 | 0.00347947 | 1.21E-13 | 0.0464 | T | G |
| rs761713 | -0.0094081 | 0.00161964 | 6.29E-09 | 0.241 | A | G |
| rs76218671 | -0.0320752 | 0.00394167 | 4.04E-16 | 0.032 | C | T |
| rs76233241 | -0.0237804 | 0.00264568 | 2.51E-19 | 0.0763 | A | C |
| rs76288880 | -0.0259615 | 0.00248178 | 1.31E-25 | 0.0906 | G | A |
| rs76351056 | -0.0286927 | 0.00331377 | 4.77E-18 | 0.0471 | C | T |
| rs7637250 | -0.0089391 | 0.00150183 | 2.65E-09 | 0.343 | G | C |
| rs76382038 | 0.0426662 | 0.00572676 | 9.31E-14 | 0.0159 | A | C |
| rs76428106 | 0.0401116 | 0.00700005 | 1.00E-08 | 0.0118 | C | T |
| rs76435567 | -0.0228884 | 0.00333604 | 6.84E-12 | 0.0482 | G | T |
| rs76453754 | 0.019173 | 0.00310464 | 6.59E-10 | 0.0521 | C | T |
| rs76463328 | -0.0189547 | 0.00286612 | 3.76E-11 | 0.0658 | T | C |
| rs76537328 | 0.0120154 | 0.0020506 | 4.64E-09 | 0.13 | G | C |
| rs76633616 | -0.0179065 | 0.00288822 | 5.65E-10 | 0.0608 | C | T |
| rs76760132 | -0.0232986 | 0.00196569 | 2.09E-32 | 0.161 | G | C |
| rs76763527 | -0.06823 | 0.00604931 | 1.67E-29 | 0.0135 | G | A |
| rs76831627 | 0.0183263 | 0.00240074 | 2.28E-14 | 0.0934 | T | G |
| rs76851192 | -0.0423861 | 0.00700014 | 1.40E-09 | 0.0112 | C | T |
| rs76856627 | -0.064158 | 0.00443331 | 1.83E-47 | 0.0286 | G | A |
| rs76895963 | 0.0544743 | 0.00562823 | 3.71E-22 | 0.018 | G | T |
| rs76900682 | -0.0129041 | 0.00196794 | 5.48E-11 | 0.154 | A | G |
| rs76918953 | 0.0402381 | 0.00424322 | 2.47E-21 | 0.0354 | T | C |
| rs7692092 | 0.00955017 | 0.00166221 | 9.17E-09 | 0.225 | T | C |
| rs76995491 | -0.0210056 | 0.00238027 | 1.10E-18 | 0.101 | G | A |
| rs7700965 | -0.0453918 | 0.00146732 | 1.00E-200 | 0.353 | C | T |
| rs77011887 | -0.0459218 | 0.00570361 | 8.19E-16 | 0.017 | T | C |
| rs77023463 | 0.0250323 | 0.00417546 | 2.03E-09 | 0.0305 | T | C |
| rs77042711 | 0.0135803 | 0.00185342 | 2.35E-13 | 0.173 | T | C |
| rs77049332 | 0.036663 | 0.00351924 | 2.05E-25 | 0.0445 | G | A |
| rs77181305 | 0.0327635 | 0.00577553 | 1.40E-08 | 0.0149 | A | G |
| rs77214878 | 0.0337054 | 0.0036609 | 3.36E-20 | 0.0465 | T | C |
| rs77241309 | -0.0291097 | 0.00239293 | 4.78E-34 | 0.0973 | C | G |
| rs77303550 | 0.0497639 | 0.00178613 | 7.86E-171 | 0.19 | T | C |
| rs77321430 | -0.041432 | 0.00214619 | 4.88E-83 | 0.129 | G | A |
| rs77348347 | -0.0211214 | 0.00286949 | 1.83E-13 | 0.0699 | T | A |
| rs77348583 | -0.0264986 | 0.00287494 | 3.05E-20 | 0.0625 | C | T |
| rs77360068 | 0.0354896 | 0.00335364 | 3.60E-26 | 0.0473 | C | T |
| rs77370158 | -0.0298349 | 0.00268623 | 1.17E-28 | 0.0767 | G | A |
| rs77380569 | -0.089721 | 0.00854836 | 9.04E-26 | 0.00856 | A | G |
| rs77384690 | -0.0615513 | 0.0102206 | 1.72E-09 | 0.00487 | C | T |
| rs7738525 | 0.0186877 | 0.00149585 | 8.15E-36 | 0.31 | G | A |
| rs77436593 | 0.0321675 | 0.00248689 | 2.86E-38 | 0.0859 | A | G |
| rs77442683 | 0.0122458 | 0.00221712 | 3.33E-08 | 0.112 | T | C |
| rs7750288 | -0.0253682 | 0.00154727 | 2.06E-60 | 0.279 | G | A |
| rs77524918 | 0.0326423 | 0.00383324 | 1.66E-17 | 0.037 | C | T |
| rs77542162 | -0.18313 | 0.00508432 | 1.00E-200 | 0.0196 | G | A |
| rs7758766 | -0.0230854 | 0.00190378 | 7.68E-34 | 0.16 | T | G |
| rs7761293 | -0.0136224 | 0.0013943 | 1.51E-22 | 0.471 | A | G |
| rs7762959 | -0.0139692 | 0.00214146 | 6.88E-11 | 0.118 | T | C |
| rs77631946 | 0.0187374 | 0.00246206 | 2.73E-14 | 0.0931 | A | C |
| rs77705851 | -0.0274678 | 0.00303825 | 1.56E-19 | 0.0574 | T | C |
| rs7773004 | -0.0116329 | 0.00140269 | 1.10E-16 | 0.49 | G | A |
| rs7777679 | 0.0213869 | 0.00356762 | 2.04E-09 | 0.0412 | G | A |
| rs7778388 | -0.010533 | 0.0014288 | 1.68E-13 | 0.381 | G | A |
| rs7778954 | -0.009334 | 0.00152253 | 8.76E-10 | 0.425 | G | T |
| rs77797599 | 0.0470693 | 0.00748557 | 3.22E-10 | 0.0106 | T | C |
| rs77871695 | 0.0116351 | 0.00201806 | 8.14E-09 | 0.138 | A | C |
| rs77875082 | -0.0538427 | 0.00421969 | 2.75E-37 | 0.0305 | A | G |
| rs77895261 | -0.0212674 | 0.00347012 | 8.86E-10 | 0.0446 | A | G |
| rs77926044 | 0.0191205 | 0.00251511 | 2.91E-14 | 0.0846 | T | C |
| rs77928629 | 0.0630001 | 0.00337191 | 6.71E-78 | 0.0452 | A | C |
| rs77960347 | -0.0699399 | 0.00619973 | 1.63E-29 | 0.0125 | G | A |
| rs7797047 | -0.0132325 | 0.00168925 | 4.75E-15 | 0.221 | G | T |
| rs77987064 | -0.0261044 | 0.0033938 | 1.45E-14 | 0.0499 | T | C |
| rs7805498 | 0.0132813 | 0.00201446 | 4.31E-11 | 0.147 | A | C |
| rs78058190 | -0.0239644 | 0.00360947 | 3.15E-11 | 0.0524 | A | G |
| rs78071106 | -0.048866 | 0.00802592 | 1.14E-09 | 0.00859 | A | G |
| rs78073763 | 0.0326534 | 0.00266614 | 1.73E-34 | 0.0782 | G | T |
| rs7807404 | 0.0202101 | 0.00248906 | 4.68E-16 | 0.0829 | C | T |
| rs7808613 | -0.011275 | 0.00163494 | 5.34E-12 | 0.245 | G | C |
| rs78152104 | -0.0290292 | 0.00505827 | 9.53E-09 | 0.0217 | G | A |
| rs78152371 | 0.0208279 | 0.00323747 | 1.25E-10 | 0.0525 | A | C |
| rs78155240 | -0.0539427 | 0.00836441 | 1.13E-10 | 0.0102 | T | G |
| rs78207142 | -0.0171787 | 0.00231154 | 1.07E-13 | 0.108 | T | C |
| rs78219104 | -0.0338964 | 0.00253507 | 8.93E-41 | 0.103 | T | C |
| rs78221564 | 0.0497155 | 0.0070289 | 1.52E-12 | 0.0112 | T | C |
| rs7827437 | 0.0094449 | 0.00153245 | 7.13E-10 | 0.308 | T | C |
| rs783175 | -0.0210921 | 0.00176359 | 5.77E-33 | 0.195 | C | G |
| rs78330067 | 0.0187323 | 0.00307569 | 1.13E-09 | 0.056 | T | A |
| rs78421006 | -0.0418455 | 0.0050457 | 1.10E-16 | 0.0201 | G | A |
| rs78590292 | -0.016369 | 0.00298172 | 4.02E-08 | 0.0592 | T | C |
| rs786416 | 0.0195673 | 0.00243497 | 9.29E-16 | 0.0911 | G | T |
| rs78643851 | -0.0348153 | 0.00538983 | 1.05E-10 | 0.0213 | T | G |
| rs7864568 | 0.0155516 | 0.00155864 | 1.91E-23 | 0.316 | A | G |
| rs78755089 | -0.0157749 | 0.00260673 | 1.43E-09 | 0.0786 | T | G |
| rs7875786 | -0.0371631 | 0.00267754 | 8.42E-44 | 0.0826 | A | G |
| rs78766179 | -0.0239845 | 0.00384884 | 4.62E-10 | 0.0367 | C | A |
| rs78824368 | 0.0170438 | 0.00277679 | 8.36E-10 | 0.0678 | A | G |
| rs7903259 | -0.0163158 | 0.00142246 | 1.86E-30 | 0.416 | G | C |
| rs7904973 | 0.0209679 | 0.00140615 | 2.77E-50 | 0.424 | G | T |
| rs7908745 | -0.0089724 | 0.00148956 | 1.71E-09 | 0.313 | G | A |
| rs79106033 | 0.0299508 | 0.00443797 | 1.49E-11 | 0.0263 | T | C |
| rs79152165 | -0.021731 | 0.00374955 | 6.81E-09 | 0.0407 | A | G |
| rs79158370 | -0.034595 | 0.0023863 | 1.26E-47 | 0.0933 | A | G |
| rs79214153 | 0.0894952 | 0.00698116 | 1.27E-37 | 0.0103 | A | G |
| rs79232531 | -0.0282259 | 0.00331291 | 1.60E-17 | 0.0501 | T | C |
| rs79242960 | -0.0206255 | 0.00358366 | 8.64E-09 | 0.0417 | T | C |
| rs7925256 | -0.0226415 | 0.00247916 | 6.68E-20 | 0.0901 | T | C |
| rs79279183 | 0.0180442 | 0.00149229 | 1.17E-33 | 0.498 | C | A |
| rs79281791 | -0.0661169 | 0.00217896 | 1.00E-200 | 0.113 | G | A |
| rs7928919 | -0.0109895 | 0.00144845 | 3.27E-14 | 0.371 | T | C |
| rs79322367 | 0.0245378 | 0.00199146 | 6.94E-35 | 0.142 | T | C |
| rs79336077 | 0.0468063 | 0.006027 | 8.09E-15 | 0.0148 | A | G |
| rs79351558 | -0.0374094 | 0.00475866 | 3.80E-15 | 0.0228 | G | A |
| rs79385701 | -0.0202363 | 0.00366617 | 3.40E-08 | 0.0382 | C | A |
| rs793900 | -0.0088241 | 0.00140878 | 3.76E-10 | 0.41 | T | C |
| rs79391862 | 0.0639367 | 0.00532487 | 3.26E-33 | 0.0202 | C | A |
| rs79409145 | 0.00992039 | 0.0017377 | 1.14E-08 | 0.227 | G | T |
| rs79429216 | -0.10433 | 0.00723806 | 4.22E-47 | 0.00999 | A | G |
| rs79614070 | 0.0328378 | 0.00377346 | 3.25E-18 | 0.036 | G | A |
| rs79623641 | 0.0179333 | 0.00282587 | 2.21E-10 | 0.0677 | A | G |
| rs7962448 | -0.0084225 | 0.00143094 | 3.96E-09 | 0.383 | A | G |
| rs79638252 | 0.152686 | 0.00562265 | 2.18E-162 | 0.0183 | A | G |
| rs79669031 | 0.036298 | 0.00538759 | 1.61E-11 | 0.0182 | A | G |
| rs79702288 | 0.0647042 | 0.00244964 | 9.52E-154 | 0.0881 | C | A |
| rs7976245 | -0.0078432 | 0.00143351 | 4.47E-08 | 0.423 | A | G |
| rs797974 | -0.0102593 | 0.00142642 | 6.37E-13 | 0.432 | C | T |
| rs79836087 | 0.0215277 | 0.0034364 | 3.74E-10 | 0.0454 | A | G |
| rs79850813 | 0.0197619 | 0.00286917 | 5.67E-12 | 0.069 | G | C |
| rs79854399 | 0.0390172 | 0.00522443 | 8.13E-14 | 0.0189 | T | C |
| rs79860339 | 0.0373705 | 0.00402871 | 1.76E-20 | 0.0322 | C | G |
| rs79868705 | 0.109229 | 0.00354924 | 1.00E-200 | 0.0398 | A | G |
| rs799157 | -0.0317878 | 0.00387352 | 2.28E-16 | 0.0363 | T | C |
| rs80085738 | 0.0407151 | 0.00683942 | 2.63E-09 | 0.0109 | A | G |
| rs80098465 | 0.0363822 | 0.00389864 | 1.04E-20 | 0.0337 | A | G |
| rs8025068 | 0.0105962 | 0.00179076 | 3.28E-09 | 0.192 | T | G |
| rs80295797 | -0.0112675 | 0.0015056 | 7.22E-14 | 0.323 | T | C |
| rs8029797 | 0.00949676 | 0.00149208 | 1.96E-10 | 0.313 | A | T |
| rs803073 | 0.00845557 | 0.00140281 | 1.66E-09 | 0.472 | A | G |
| rs80350583 | -0.0421808 | 0.00579139 | 3.26E-13 | 0.0159 | A | G |
| rs8038512 | 0.00924136 | 0.00148063 | 4.33E-10 | 0.356 | T | G |
| rs8041391 | -0.0120609 | 0.00199109 | 1.38E-09 | 0.153 | T | C |
| rs8051691 | 0.00885158 | 0.00155442 | 1.24E-08 | 0.278 | T | G |
| rs8053037 | -0.0520414 | 0.00685897 | 3.27E-14 | 0.0163 | C | T |
| rs8059738 | 0.0279534 | 0.00302165 | 2.22E-20 | 0.0627 | G | A |
| rs8062453 | -0.0119927 | 0.0014511 | 1.40E-16 | 0.431 | T | G |
| rs8076992 | 0.0235712 | 0.00280367 | 4.20E-17 | 0.0644 | G | T |
| rs808994 | 0.0132759 | 0.00174531 | 2.81E-14 | 0.197 | T | C |
| rs8090922 | -0.0093483 | 0.00148577 | 3.14E-10 | 0.32 | G | T |
| rs8093891 | 0.00951967 | 0.001406 | 1.28E-11 | 0.449 | T | C |
| rs8100828 | -0.0574975 | 0.00320484 | 5.66E-72 | 0.0508 | T | C |
| rs8102912 | 0.104184 | 0.00172082 | 1.00E-200 | 0.217 | A | G |
| rs8106228 | -0.009743 | 0.00176997 | 3.70E-08 | 0.197 | A | G |
| rs8106605 | 0.0115533 | 0.00198922 | 6.32E-09 | 0.148 | G | T |
| rs8110479 | 0.124497 | 0.00363328 | 1.00E-200 | 0.0424 | T | C |
| rs811053 | 0.0138785 | 0.00165871 | 5.91E-17 | 0.243 | T | A |
| rs8112559 | 0.0281466 | 0.00213315 | 9.39E-40 | 0.119 | G | C |
| rs8113274 | 0.0125317 | 0.00166794 | 5.77E-14 | 0.222 | A | T |
| rs8113311 | 0.0721755 | 0.0015791 | 1.00E-200 | 0.277 | T | G |
| rs8191713 | -0.0503031 | 0.00469159 | 8.03E-27 | 0.0225 | A | G |
| rs846848 | 0.0219099 | 0.00201462 | 1.51E-27 | 0.14 | G | T |
| rs872668 | -0.0125922 | 0.00194975 | 1.06E-10 | 0.151 | A | G |
| rs873870 | 0.0167108 | 0.00139326 | 3.82E-33 | 0.475 | A | G |
| rs886336 | -0.0083414 | 0.00142649 | 4.99E-09 | 0.416 | G | A |
| rs887829 | 0.0137912 | 0.00147776 | 1.03E-20 | 0.324 | T | C |
| rs890576 | 0.0152847 | 0.00174823 | 2.27E-18 | 0.202 | G | C |
| rs891202 | -0.00937 | 0.00145903 | 1.34E-10 | 0.367 | T | C |
| rs896311 | 0.0165774 | 0.00152682 | 1.84E-27 | 0.3 | G | A |
| rs896738 | 0.0287077 | 0.00422243 | 1.05E-11 | 0.0302 | G | A |
| rs900776 | 0.0195692 | 0.00187636 | 1.82E-25 | 0.17 | C | A |
| rs913499 | -0.011274 | 0.00138898 | 4.79E-16 | 0.482 | A | G |
| rs921679 | -0.0085183 | 0.00139111 | 9.16E-10 | 0.45 | C | A |
| rs921719 | 0.00912448 | 0.00141425 | 1.11E-10 | 0.492 | G | T |
| rs9258405 | -0.0156749 | 0.00273691 | 1.02E-08 | 0.075 | T | C |
| rs9264803 | -0.0087081 | 0.00152445 | 1.11E-08 | 0.332 | T | C |
| rs9269111 | 0.0144419 | 0.00180468 | 1.22E-15 | 0.214 | G | A |
| rs9271788 | -0.0162429 | 0.00167304 | 2.77E-22 | 0.449 | G | A |
| rs9286381 | -0.0357499 | 0.00393644 | 1.07E-19 | 0.0353 | C | T |
| rs928911 | -0.0274881 | 0.00342181 | 9.50E-16 | 0.0449 | T | C |
| rs9297994 | -0.0355062 | 0.00146485 | 8.68E-130 | 0.338 | G | A |
| rs9298506 | -0.0245553 | 0.00170664 | 6.15E-47 | 0.208 | G | A |
| rs9302635 | 0.0422921 | 0.00179635 | 1.47E-122 | 0.184 | C | T |
| rs9311677 | 0.0132028 | 0.00145451 | 1.11E-19 | 0.353 | C | G |
| rs9315148 | 0.0105079 | 0.00160371 | 5.67E-11 | 0.25 | T | C |
| rs9332408 | -0.0123167 | 0.00139865 | 1.30E-18 | 0.472 | C | T |
| rs9347330 | -0.0104853 | 0.0015075 | 3.52E-12 | 0.337 | A | T |
| rs9349646 | -0.010191 | 0.00142412 | 8.31E-13 | 0.473 | C | T |
| rs9368188 | 0.00914981 | 0.00147238 | 5.16E-10 | 0.336 | A | G |
| rs9370135 | 0.0140488 | 0.00145536 | 4.77E-22 | 0.349 | T | A |
| rs9373125 | -0.0156511 | 0.00165403 | 3.01E-21 | 0.227 | C | T |
| rs9398803 | 0.00957246 | 0.00139876 | 7.73E-12 | 0.474 | G | A |
| rs9398815 | 0.010412 | 0.00142557 | 2.80E-13 | 0.457 | C | T |
| rs9399137 | 0.0255597 | 0.00158355 | 1.32E-58 | 0.262 | C | T |
| rs9405990 | 0.00845261 | 0.00138618 | 1.08E-09 | 0.497 | A | G |
| rs9410207 | 0.0200359 | 0.00282853 | 1.41E-12 | 0.0656 | C | T |
| rs941796 | -0.012507 | 0.00141788 | 1.14E-18 | 0.399 | A | G |
| rs943643 | 0.0192489 | 0.00145041 | 3.39E-40 | 0.397 | T | C |
| rs9438624 | -0.0100677 | 0.00180421 | 2.40E-08 | 0.194 | C | T |
| rs9438862 | -0.0086319 | 0.00157914 | 4.60E-08 | 0.274 | A | G |
| rs9442228 | 0.00881424 | 0.00153762 | 9.90E-09 | 0.306 | T | G |
| rs9456496 | -0.0330605 | 0.00223769 | 2.14E-49 | 0.11 | G | A |
| rs9472125 | 0.0155888 | 0.00238159 | 5.93E-11 | 0.111 | T | C |
| rs9480534 | 0.0169154 | 0.00242107 | 2.81E-12 | 0.0918 | G | A |
| rs9496567 | 0.0209744 | 0.00164372 | 2.73E-37 | 0.236 | A | G |
| rs9500877 | -0.0187933 | 0.00184757 | 2.65E-24 | 0.179 | C | T |
| rs9508741 | -0.0105233 | 0.00191558 | 3.94E-08 | 0.24 | T | A |
| rs9526495 | 0.0292843 | 0.00449318 | 7.15E-11 | 0.0286 | G | C |
| rs9535755 | -0.0114717 | 0.00183435 | 4.01E-10 | 0.172 | G | C |
| rs9543965 | 0.00998888 | 0.00180422 | 3.09E-08 | 0.187 | A | G |
| rs954680 | 0.0123572 | 0.0015382 | 9.47E-16 | 0.298 | C | G |
| rs958225 | 0.0227752 | 0.00308953 | 1.68E-13 | 0.0541 | A | T |
| rs9592980 | 0.0095033 | 0.00142143 | 2.30E-11 | 0.415 | G | A |
| rs9597 | 0.0210568 | 0.00379106 | 2.79E-08 | 0.0364 | G | C |
| rs9616822 | -0.0113696 | 0.00144701 | 3.92E-15 | 0.359 | A | G |
| rs964184 | -0.0552411 | 0.00201933 | 9.12E-165 | 0.135 | G | C |
| rs9653945 | 0.012963 | 0.00145387 | 4.83E-19 | 0.347 | A | G |
| rs9749225 | -0.0114873 | 0.00163993 | 2.47E-12 | 0.251 | T | A |
| rs978458 | -0.0119449 | 0.00158367 | 4.61E-14 | 0.263 | T | C |
| rs9828317 | 0.00817202 | 0.00148585 | 3.80E-08 | 0.335 | T | G |
| rs9837149 | -0.0188877 | 0.0019215 | 8.39E-23 | 0.153 | C | G |
| rs9837622 | 0.0370846 | 0.00270523 | 9.03E-43 | 0.073 | A | T |
| rs9862203 | 0.0131919 | 0.00163034 | 5.89E-16 | 0.24 | A | G |
| rs9893349 | -0.0141802 | 0.00189262 | 6.76E-14 | 0.168 | G | A |
| rs9904189 | 0.00949941 | 0.00151425 | 3.53E-10 | 0.401 | T | A |
| rs9909417 | -0.0093007 | 0.00155538 | 2.24E-09 | 0.284 | A | G |
| rs9911983 | -0.0078342 | 0.0014162 | 3.17E-08 | 0.436 | C | T |
| rs9915487 | 0.00895924 | 0.00160872 | 2.56E-08 | 0.277 | T | C |
| rs9921494 | -0.0154808 | 0.00228866 | 1.34E-11 | 0.138 | A | C |
| rs9921780 | -0.0087057 | 0.00140753 | 6.21E-10 | 0.427 | G | A |
| rs9937165 | 0.0110235 | 0.00160754 | 7.01E-12 | 0.252 | G | T |
| rs9938539 | 0.0188436 | 0.00149265 | 1.55E-36 | 0.36 | C | T |
| rs994286 | 0.0354843 | 0.00492204 | 5.63E-13 | 0.0227 | A | G |
| rs9960523 | -0.0091878 | 0.0014683 | 3.91E-10 | 0.342 | T | C |
| rs9987289 | 0.0598584 | 0.00239036 | 2.16E-138 | 0.0918 | A | G |

**Table S2.** Uncorrelated (r2<0.1) instrumental variants at p<5x10^-8 for 1-SD lower LDL in the PCSK9 gene region. EAF = Effect allele frequency; Beta = Coefficient of effect allele; SE = Standard error

| **SNP** | **Beta** | **SE** | **P-value** | **EAF** | **Effect allele** | **Other allele** |
| --- | --- | --- | --- | --- | --- | --- |
| rs10493176 | 0.0710446 | 0.002514 | 1.09E-175 | 0.086 | G | T |
| rs11206510 | 0.0609559 | 0.00180723 | 1.00E-200 | 0.182 | C | T |
| rs11206517 | -0.0888589 | 0.00371072 | 1.00E-126 | 0.0371 | G | T |
| rs11591147 | 0.433696 | 0.005426 | 1.00E-200 | 0.0163 | T | G |
| rs137886411 | -0.052263 | 0.00746464 | 2.53E-12 | 0.01 | A | G |
| rs142116310 | -0.0554039 | 0.00723134 | 1.84E-14 | 0.0107 | A | G |
| rs142236283 | 0.0526683 | 0.00456022 | 7.42E-31 | 0.0258 | A | G |
| rs17111503 | -0.0487264 | 0.00160687 | 1.00E-200 | 0.256 | G | A |
| rs17192725 | -0.0379882 | 0.00253634 | 1.03E-50 | 0.0876 | A | G |
| rs2479408 | 0.0445077 | 0.00180457 | 2.61E-134 | 0.199 | G | C |
| rs2495477 | 0.0524078 | 0.00149171 | 1.00E-200 | 0.397 | G | A |
| rs2495494 | -0.0234762 | 0.00320077 | 2.22E-13 | 0.0562 | C | G |
| rs2495497 | -0.022648 | 0.00211201 | 7.90E-27 | 0.131 | T | C |
| rs28385715 | -0.0455686 | 0.00489729 | 1.34E-20 | 0.0229 | G | T |
| rs41294821 | 0.0427234 | 0.00501678 | 1.65E-17 | 0.0206 | T | C |
| rs41294825 | 0.0414044 | 0.00341298 | 7.20E-34 | 0.043 | T | A |
| rs41297885 | 0.0406301 | 0.00378858 | 7.82E-27 | 0.0358 | G | C |
| rs74700387 | 0.0416223 | 0.00554408 | 6.03E-14 | 0.0161 | T | C |
| rs75050571 | -0.0282488 | 0.00498383 | 1.44E-08 | 0.0236 | T | A |
| rs7525503 | -0.0712849 | 0.00498832 | 2.51E-46 | 0.0229 | T | G |
| rs77011887 | -0.0459218 | 0.00570361 | 8.19E-16 | 0.017 | T | C |
| rs79844613 | 0.0305776 | 0.0044842 | 9.17E-12 | 0.0259 | A | G |
| rs80085738 | 0.0407151 | 0.00683942 | 2.63E-09 | 0.0109 | A | G |

**Table S3.** Uncorrelated (r2<0.1) instrumental variants at p<5x10^-8 for lower normalized circulating PCSK9 protein levels. EAF = Effect allele frequency; Beta = Coefficient of effect allele; SE = Standard error

| **SNP** | **Beta** | **SE** | **P-value** | **EAF** | **Effect allele** | **Other allele** |
| --- | --- | --- | --- | --- | --- | --- |
| rs10493176 | 0.0551083 | 0.00727671 | 3.54E-14 | 0.207279 | G | T |
| rs11206510 | 0.0477757 | 0.00559559 | 1.40E-17 | 0.159935 | C | T |
| rs11591147 | 0.29342 | 0.0200121 | 9.20E-49 | 0.0359517 | T | G |
| rs17111503 | -0.0427734 | 0.00550264 | 7.55E-15 | 0.200558 | G | A |
| rs2495477 | 0.0457846 | 0.00457753 | 1.40E-23 | 0.435254 | G | A |

**Table S4.** Uncorrelated (r2<0.1) instrumental variants at p<5x10^-8 for lower PCSK9 gene expression in the liver. EAF = Effect allele frequency; Beta = Coefficient of effect allele; SE = Standard error

| **SNP** | **Beta** | **SE** | **P-value** | **EAF** | **Effect allele** | **Other allele** |
| --- | --- | --- | --- | --- | --- | --- |
| rs693668 | -0.179232 | 0.0260331 | 1.79E-11 | 0.577172 | A | G |

**Table S5.** Proportion of variance in phenotype explained by instrumental variants, number of instrumental variants, number of participants in genome-wide association study, and combined F-statistics for instruments. R2 = instrumental variants; N_SNP_ = number of instrumental variants; N_CASE_ = number of participants in genome-wide association study; F-statistic = combined F-statistics for instruments

| **Phenotype** | **R2** | **N_SNP_** | **N_CASE_** | **F-statistic** |
| --- | --- | --- | --- | --- |
| **LDL** | 0.310163004 | 2014 | 1320016 | 294.2386949 |
| **LDL via PCSK9** | 0.012905317 | 23 | 1320016 | 750.3317835 |
| **PCSK9 eQTL (Liver)** | 0.015679424 | 1 | 226 | 3.568137201 |
| **PCSK9 pQTL (Whole Blood)** | 0.009196568 | 5 | 10186 | 18.89800935 |

**Table S6.** Posterior probabilities for the PCSK9 gene region obtained via Bayesian tests for genetic colocalization. PP = posterior probability; PP.H0= Posterior probability for Hypothesis 0 (no causal variants) in the colocalization analysis; PP.H1= Posterior probability for Hypothesis 1 (causal variant for low-density lipoprotein cholesterol, LDL) in the colocalization analysis; PP.H2= Posterior probability for Hypothesis 2 (causal variant for congenital malformation) in the colocalization analysis; PP.H3= Posterior probability for Hypothesis 3 (distinct causal variants) in the colocalization analysis; PP.H4= Posterior probability for Hypothesis 4 (shared causal variants) in the colocalization analysis.

| **Outcome** | **PPH0** | **PPH1** | **PPH2** | **PPH3** | **PPH4** |
| --- | --- | --- | --- | --- | --- |
| **Congenital malformations of cardiac septa** | 0.00% | 91.28% | 0.00% | 2.14% | 6.58% |
| **Congenital malformations of the circulatory system** | 0.00% | 89.62% | 0.00% | 2.02% | 8.35% |
| **Congenital malformations of the musculoskeletal system** | 0.00% | 94.34% | 0.00% | 1.68% | 3.98% |
| **Congenital malformations of eye, ear, face and neck** | 0.00% | 93.86% | 0.00% | 1.99% | 4.16% |
| **Congenital obstructive defects of renal pelvis and ureter** | 0.00% | 84.17% | 0.00% | 4.60% | 11.23% |
| **Congenital malformations of the digestive system** | 0.00% | 92.65% | 0.00% | 2.32% | 5.03% |
| **Congenital malformations of skin** | 0.00% | 42.94% | 0.00% | 1.32% | 55.75% |
| **Congenital malformations affecting multiple systems** | 0.00% | 79.71% | 0.00% | 2.54% | 17.74% |
| **Vertebral, anorectal, cardiovascular, tracheo-esophageal, renal and limb anomaly** | 0.00% | 79.32% | 0.00% | 5.97% | 14.71% |

**Table S7.** Phenome-wide scan demonstrating the phenotypic traits associated with the genetic variants used as instrumental variables proxying lipid lowering via PCSK9 and low-density lipoprotein overall and PCSK9 pQTL and eQTLs. eQTL = Expression quantitative trait loci, pQTL = Protein quantitative trait loci, SNP = Single nucleotide polymorphism, PCSK9 = Proprotein convertase subtilisin–kexin type 9.
